# Stable Meta-Networks, Noise, and Artifacts in the Human Connectome: Low- to High-Dimensional Independent Components Analysis as a Hierarchy of Intrinsic Connectivity Networks

**DOI:** 10.3389/fnins.2021.625737

**Published:** 2021-05-06

**Authors:** Korey P. Wylie, Eugene Kronberg, Kristina T. Legget, Brianne Sutton, Jason R. Tregellas

**Affiliations:** ^1^Department of Psychiatry, University of Colorado School of Medicine, Aurora, CO, United States; ^2^Department of Neurology, University of Colorado School of Medicine, Aurora, CO, United States; ^3^Research Service, Rocky Mountain Regional VA Medical Center, Aurora, CO, United States

**Keywords:** ICA, model order, connectivity, resting-state, multi-scale, multi-resolution, multivariate analysis

## Abstract

Connectivity within the human connectome occurs between multiple neuronal systems—at small to very large spatial scales. Independent component analysis (ICA) is potentially a powerful tool to facilitate multi-scale analyses. However, ICA has yet to be fully evaluated at very low (10 or fewer) and ultra-high dimensionalities (200 or greater). The current investigation used data from the Human Connectome Project (HCP) to determine the following: (1) if larger networks, or meta-networks, are present at low dimensionality, (2) if nuisance sources increase with dimensionality, and (3) if ICA is prone to overfitting. Using bootstrap ICA, results suggested that, at very low dimensionality, ICA spatial maps consisted of Visual/Attention and Default/Control meta-networks. At fewer than 10 components, well-known networks such as the Somatomotor Network were absent from results. At high dimensionality, nuisance sources were present even in denoised high-quality data but were identifiable by correlation with tissue probability maps. Artifactual overfitting occurred to a minor degree at high dimensionalities. Basic summary statistics on spatial maps (maximum cluster size, maximum component weight, and average weight outside of maximum cluster) quickly and easily separated artifacts from gray matter sources. Lastly, by using weighted averages of bootstrap stability, even ultra-high dimensional ICA resulted in highly reproducible spatial maps. These results demonstrate how ICA can be applied in multi-scale analyses, reliably and accurately reproducing the hierarchy of meta-networks, large-scale networks, and subnetworks, thereby characterizing cortical connectivity across multiple spatial scales.

## Introduction

Network models of neural processing used in neuroimaging are continually evolving and becoming increasingly sophisticated. Analytic advances in the past decade have identified major canonical large-scale networks involved in cognition (Raichle et al., 2001; Greicius et al., 2003; Vincent et al., 2008). More recent work has identified subnetworks within these larger networks (Smith et al., 2009; Andrews-Hanna et al., 2010; Shirer et al., 2012) and even smaller regional parcellations (Glasser et al., 2016). This network topology, encompassing multiple spatial scales, has long been hypothesized as a fundamental architecture of cognitive neuroscience (Churchland and Sejnowski, 1988) and is now the focus of active investigation (Park and Friston, 2013; Sporns, 2015; Betzel and Bassett, 2017; Eickhoff et al., 2018). However, analytic tools needed to investigate multi-scale processing have yet to be fully developed for independent component analysis (ICA), a powerful and widely used tool in network analyses.

As an example of multi-scale processing, consider the following thought experiment. When is a visual stimulus only processed within the primary visual cortex—on a relatively small spatial scale? When is it processed widely throughout the entire visual system—on a larger spatial scale? Under what circumstances will processing extend beyond the visual system, perhaps to encompass attention systems as well? While a simple line displayed on a screen may result in processing localized to a single circumscribed region, a complex visual recognition task may result in widespread engagement of occipitotemporal and occipitoparietal pathways (Kandel et al., 2000).

This simplified thought experiment suggests that the visual system processes information across a wide range of scales—from the small to the large. Similarly, other systems feature a multi-scale organization. Processing can be localized to a small region, distributed across multiple large-scale networks, or at an intermediate scale, depending on the properties of the stimuli (Churchland and Sejnowski, 1988; Sporns, 2015; Betzel and Bassett, 2017; Eickhoff et al., 2018). In practice, however, most current analyses investigate neural processing at a single scale by applying a single region-of-interest atlas or network parcellation. In these analyses, processing and connectivity occurring at smaller scales are obscured due to regional averaging. Processing and connectivity occurring over larger scales may be hidden or missed by the narrow analytic focus. Additional techniques are needed to investigate the full range of multi-scale processing.

Multi-scale processing can be investigated using multiple techniques, such as hierarchical clustering (Doucet et al., 2011; Yeo et al., 2011; Thirion et al., 2014; Gotts et al., 2020), hierarchical modularity (Meunier et al., 2009), fuzzy-c-means clustering (Lee et al., 2012), multi-level k-means clustering (Bellec et al., 2010), gradient-weighted Markov random field models (Schaefer et al., 2018), non-negative matrix factorization (Li et al., 2018), or multi-scale ICA (Iraji et al., 2021). Similarly, multi-granularity analyses segment the brain into interrelated spatial scales by applying multiple gray matter atlases (Arslan et al., 2018; Gong et al., 2018). These results have shown that the brain is organized as multiple large-scale networks, or intrinsic connectivity networks (ICNs). ICNs typically encompass regions from multiple cortical lobes, with smaller subnetworks nested within in a hierarchical structure. The two largest networks encompass task-negative regions and task-positive regions. Within each are nested subnetworks encompassing canonical large-scale networks such as the default mode network (DMN), frontoparietal control network (FPCN), and visual network. The large task-negative network encompasses the DMN as well as the FPCN (Meunier et al., 2009; Doucet et al., 2011; Lee et al., 2012; Gotts et al., 2020), while the task-positive network encompasses visual as well as other primary sensory and attention systems.

Multi-model order ICA (Abou−Elseoud et al., 2010) may represent an alternative method for multi-scale analyses. In fact, although ICA does not impose a hierarchy upon the data, ICA networks nevertheless appear to correspond to a nested hierarchy of networks. This hierarchical structure is broadly similar to many network features observed in other analyses (Kiviniemi et al., 2009; Abou−Elseoud et al., 2010). Multi-model order ICA was recently extended to investigate dynamic interactions between multiple scales (Iraji et al., 2021). Additionally, high-dimensional ICA can reliably estimate neural processing and connectivity across a wide range of spatial scales, well beyond the limits of other hierarchical methods. ICA is capable of reliably and reproducibly estimating 70 networks in a single analysis (Abou−Elseoud et al., 2010). Even more networks may be examined using ultra-high-dimensional ICA in analyses involving 700 networks or more (Iraji et al., 2019). However, ICA is not without its own limitations, including potential instability at a very high dimensionality of 100 or more networks.

The overall goal of the current study is to evaluate the potential of multi-model order ICA as a method for multi-scale analysis and to address its potential limitations. In the current investigation, the ICA model order was sequentially adjusted across a wide range, effectively treating the parameter as a tuning knob (Betzel and Bassett, 2017). This simple yet effective approach demonstrates how this widely used network analysis tool can, without modification, be applied in a multi-scale analysis.

The specific goals of the current study are to explore the challenges presented by this approach, including potential limitations of ICA. The first goal is to determine if low-dimensional ICA results are in close agreement with the large meta-networks commonly found using hierarchical methods. Specifically, the initial partition into task-positive and task-negative meta-networks has yet to be reported using low-dimensional ICA. If confirmed, this will establish that the major features of multi-scale networks are not dependent on analytic techniques.

The second goal is to investigate the stability of ICA results at very high dimensionality, including potential contamination by noise and nuisance artifacts. These artifacts may be present even in denoised data due to limitations of current denoising strategies (Parkes et al., 2018). Furthermore, noise and nuisance artifacts may be increasingly prominent at high dimensionality due to increasing degrees of freedom in the analysis. ICA model orders greater than 70 have been shown to be unstable and unlikely to replicate (Abou−Elseoud et al., 2010). The increasing instability with model order may be due to the increasing prominence of artifacts in higher-dimensional ICA. Currently, noise and nuisance artifacts are identified using subjective criteria, such as by visual inspection of ICA spatial maps. However, visual inspection is time-consuming and infeasible at high dimensions (De Martino et al., 2007; Sochat et al., 2014). Easily automated quantitative criteria that measure the same qualitative attributes used in visual inspection and that identify the same artifactual signals are necessary to facilitate independent component analyses at a very high dimensionality. This would establish that ICA can be utilized to investigate the fine-grained spatial scales of the hierarchy of networks, in addition to the coarser spatial scales observable with hierarchical techniques.

We hypothesized that, at a very low dimensionality of fewer than five components, ICA would result in aggregations of two or more canonical ICNs as a network of networks, or meta-network, similar to the task-positive and task-negative observed using hierarchical methods (Meunier et al., 2009; Doucet et al., 2011; Lee et al., 2012; Gotts et al., 2020). We also hypothesized that components representing nuisance signals in the white matter (WM) or cerebrospinal fluid (CSF) or from artifactual overfitting [“spikes and bumps” (Hyvärinen et al., 1999; Särelä and Vigário, 2003)] would be readily identifiable with relatively simple quantitative criteria based on the qualitative criteria used in visual inspection. Lastly, we hypothesized that when these potential artifactual spatial maps are identified and removed from the analysis, the remaining ICA components would be stable and replicable with bootstrap stability analysis. If confirmed, these hypotheses would suggest that ICA can be used to investigate the full richness of multi-scale processing.

## Materials and Methods

### Subjects

Data used in the preparation of this work were obtained from the Human Connectome Project (HCP) database^1^. The HCP project (principal investigators: Bruce Rosen, MD, Ph.D. Martinos Center at Massachusetts General Hospital; Arthur W. Toga, Ph.D., University of Southern California; Van J. Weeden, MD, Martinos Center at Massachusetts General Hospital) is supported by the National Institute of Dental and Craniofacial Research (NIDCR), the National Institute of Mental Health (NIMH), and the National Institute of Neurological Disorders and Stroke (NINDS). Collectively, the HCP is the result of efforts of co-investigators from the University of Southern California, Martinos Center for Biomedical Imaging at Massachusetts General Hospital (MGH), Washington University, and the University of Minnesota.

Data from healthy participants in the HCP S1200 release^2^ were included in the current investigation, with “healthy” defined broadly by the HCP in order to recruit a sample representative of the general population (Van Essen et al., 2013). Specifically, subjects were excluded for severe neurodevelopmental disorders (e.g., autism), neuropsychiatric disorders (e.g., schizophrenia), neurologic disorders (e.g., Parkinson’s disease), and vascular illness that may negatively impact data quality (e.g., diabetes or hypertension). Only subjects with available rs-fMRI scans were included. With these criteria, the sample size for the current investigation was 1,084 (males = 497, females = 587, mean age = 28.8 ± 3.7 years). All subjects provided informed consent (Van Essen et al., 2013).

### Imaging Acquisition and Preprocessing

Individual resting-state fMRI scans were acquired and preprocessed by the HCP. Data were acquired on a Siemens 3T Skyra scanner with a 32-channel head coil. MRI acquisition parameters [published previously (Smith et al., 2013)] were as follows: total scan rs-fMRI duration of 15 min with a multiband acceleration factor of eight and a TR of 720 ms, resulting in 1,200 volumes with isotropic 2.0-mm voxels. Preprocessing included gradient distortion correction, head motion correction, bias field removal, T1-weighted image registration, intensity normalization, and weak high-pass filtering (>2,000 s FWHM). Common noise sources, corresponding to head-motion and cardiac artifacts, as well as signals from CSF and WM sources were identified and removed using ICA-FIX by the HCP (Smith et al., 2013; Salimi-Khorshidi et al., 2014).

Independent component analysis was implemented using the GIFT toolbox v3.0b^3^. Resting-state scans were masked by the whole brain template provided by HCP. Data were first normalized to voxel variances. Two data reduction steps, subject and group levels, were applied. Subject-level principal component analysis (PCA) was estimated with singular value decomposition, with a final dimension of 300. Group-level PCA was estimated with Multi Power Iteration [MPOWIT (Rachakonda et al., 2016)], with a final dimension determined by the ICA model order. Group ICA was then estimated using the Infomax algorithm (Bell and Sejnowski, 1995). Following ICA, whole-brain spatial maps were back-reconstructed for each ICA component (IC) and each individual subject using GICA3 (Erhardt et al., 2011). IC weights within each spatial map were centered and scaled by subtracting the volume mean and dividing by the volume standard deviation.

### Independent Component Analysis Model

Spatial ICA represents the data as a linear combination of statistically independent source signals, with the generative model (Hyvarinen et al., 2001):

(1) $$X=\sum_{k=1}^{K}a_{k}\,s_{k}^{T}$$

where **X** is an *N* × *V* matrix containing fMRI data for *N* time points from *V* voxels, s*_*k*_* is the spatial map of the *k*th independent source as a *V* × 1 vector containing the IC weights, a*_*k*_* is a *N* × 1 vector containing temporal dynamics for the *k*th independent component, *K* is the number of source signals (i.e., ICA model order), and uppercase superscript *T* (e.g., **X***^*T*^*) indicates matrix transposition. If the vectors **a***_*k*_* are concatenated into the mixing matrix **A**, and similarly with the **s***_*k*_* into the source matrix S, Equation 1 then becomes the more succinct notation *X* = AS.

When applied to fMRI, group ICA is performed by temporally concatenating voxel time series across all subjects (Calhoun et al., 2001). For any single ICA model, *K* is a fixed parameter, while **a***_*k*_* and **s***_*k*_* are estimated simultaneously for all components using the principle of non-Gaussianity (Hyvarinen et al., 2001). For the current analysis, separate ICA models were fitted to the data, with varying model order parameter *K* in Equation 1. For low-dimensional ICA, model orders of *K* = 2, 3, 4, 5, 6, 7, 8, 9, and 10 components were analyzed. Subsequently, model orders of *K* ranging from 10 to 300 in steps of 10 were analyzed (i.e., *K* = 10, 20,…, 290, 300 components). To facilitate comparison between different model orders, ICA*_*K*_* will denote Equation 1 with model order *K*.

### ICASSO and Bootstrap Reproducibility

ICASSO is a bootstrap resampling method for ICA (Himberg et al., 2004). ICASSO elegantly resolves the sign, scale, and permutation ambiguities inherent in Equation 1 that would otherwise prevent bootstrap resampling. Additionally, ICASSO provides a powerful measure of IC reproducibility, the bootstrap stability index *I*_*q*_. Since *I*_*q*_ is a key technique used in the current analysis, an overview of ICASSO and the terms used in the calculation of *I*_*q*_ will be provided in this section.

At each bootstrap replicate, PCA-reduced data from a random subset of subjects are chosen with replacement (i.e., duplicate data from the same subject is allowed). All subjects are used to select the bootstrap sample. The bootstrap sample size is equal to the original sample size (i.e., *n* = 1,084). The resulting bootstrapped dataset is then temporally concatenated and entered into an ICA with model order *K* as above. Since ICA components are unordered, it is initially unclear which bootstrap spatial maps correspond to which spatial maps in the original data. ICASSO resolves this ambiguity using hierarchical clustering (Hastie et al., 2009) to group bootstrap replicates based on their similarity with spatial maps in the original data. A dissimilarity measure, based on the absolute value of the correlation between spatial maps, is used to construct a dendrogram. The dendrogram is then cut at a level corresponding to the ICA model order *K*. This procedure parcellates the set of all spatial maps into disjoint clusters, with spatial maps within each cluster corresponding to bootstrap replicates of an original spatial map.

If an IC spatial map is highly reproducible and stable, its bootstrap replicates will be nearly identical. Consequently, correlations between these bootstrap replicate spatial maps will be nearly perfect. Conversely, if an IC spatial map is unstable, its bootstrap replicates will be highly variable and correlations between them will be low. This intuition can be formalized using the bootstrap stability index metric.

The reproducibility of a spatial map under bootstrap resampling is measured by the stability index, *I*_*q*_. Let *B*_*k*_ denote the set of all bootstrap replicates corresponding to the original spatial map **s***_*k*_*, and let #{*B*} denote the cardinality of set *B*, i.e., the number of elements it contains. The bootstrap stability index for component *k* is then as follows:

(2) $$I_{q}\,\left(B_{k}\right)=\frac{1}{a_{k}^{2}}\,\sum_{i,j\in B_{k}}\left|r_{i\,j}\right|-\frac{1}{a_{k}\,b_{k}}\,\sum_{i\in B_{k}}\sum_{j\notin B_{k}}\left|r_{i\,j}\right|,$$

$$a_{k}=\#\,\left\{B_{k}\right\},b_{k}=\sum_{l\neq k}\#\,\left\{B_{l}\right\}.$$

where *r*_*ij*_ is the correlation coefficient between spatial maps **s***_*i*_* and **s***_*j*_*, and | *r*| denotes the absolute value of *r*. Descriptively, the first term in this equation is the average similarity between bootstrap replicates of the same spatial map, while the second term is the average similarity with bootstrap replicates of different spatial maps. *I*_*q*_(*B*_*k*_) is equal to one for an ideal cluster with perfect replication and decreases as the bootstrap replicates of a spatial map become unstable.

Bootstrap stability index *I*_*q*_ was evaluated using sample quantiles (Shao, 2003) for each ICA model order. Bootstrap convergence for each quantile was confirmed by fixing the ICA model order at 20 and varying the number of bootstrap replicates from 10 to 100. All quantiles converged with as few as 20 replicates, with the exception of the minimum *I*_*q*_ (Supplementary Figure 1). To confirm that this was not affected by ICA model order, bootstrap convergence was then repeated at a higher model order of 70 and the number of bootstrap replicates similarly varied. Again, all quantiles converged quickly with as few as 20 replicates, with the exception of the minimum *I*_*q*_ (Supplementary Figure 1). Based on this analysis, 50 bootstrap replicates were chosen for all ICASSO runs in order to ensure convergence of all but the minimum *I*_*q*_ across the varying conditions of the analysis. Statistical inference on *I*_*q*_ quantiles was carried out by permuting cluster membership 1,000 times per ICA model order.

### Analysis of Component Spatial Maps

The aim of the current investigation was to quantitatively describe ICA spatial maps across a wide range of model orders. In this section, the quantitative tools used in the current analysis will be introduced. In the section “Voxel Inclusion Probabilities and Volumes”, novel analytic tools, Voxel Inclusion Probabilities, and resulting Voxel Inclusion Probability Volumes will be introduced. Voxel Inclusion Probability Volumes are used to display quantitative measures as a whole-brain statistical map in subsequent sections. Sections “Independent Component Analysis Multi-Scale Network Topology” through “Network Subdividing and Bootstrap Instability” describe the quantitative measures that will be used in the analysis. Each measure will be introduced by a motivating research question, followed by a summary of its interpretation.

#### Voxel Inclusion Probabilities and Volumes

**FIGURE 1:**
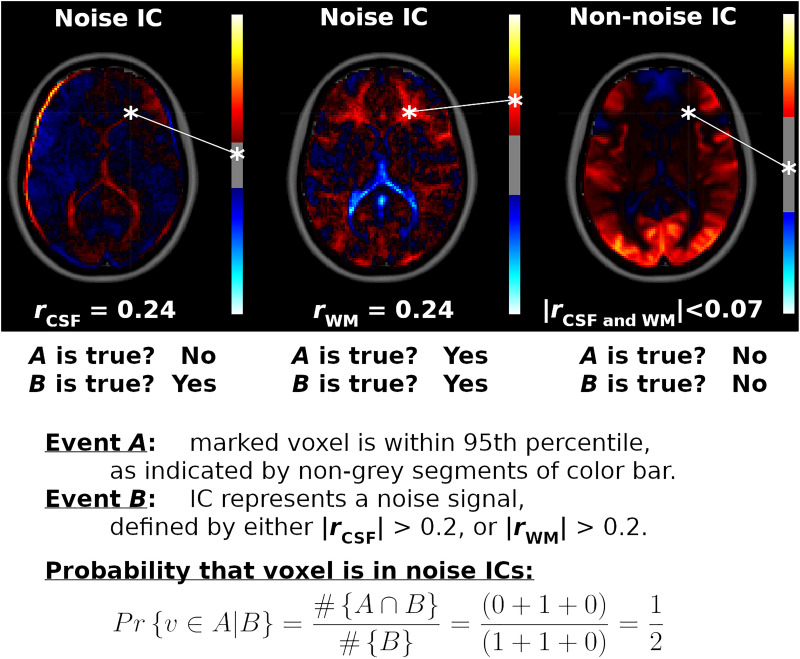
Example Voxel Inclusion Probability Calculation. Shown are spatial maps for three independent component analysis (ICA) components (ICs), each with a color bar thresholded at the 95th percentile and correlations with cerebrospinal fluid (CSF) and white matter (WM) tissue probability maps. From left to right, displayed are spatial maps for component number 36 from ICA model order 60 (ICA_60,36_), ICA_270,81_, and ICA_2,1_, respectively. ICA_60,36_ and ICA_270,81_ correlated strongly with CSF and WM, respectively, while ICA_2,1_ did not correlate substantially with either noise source (*r*_*CSF*_ = 0.07, *r*_*WM*_ = –0.03). ICA weights for a voxel marked by an asterisk are mapped onto each color bar. For the first IC, this voxel is less than the cutoff for the 95th percentile (IC weight = 0.09, | cutoff| = 0.26, range = ± 1.6). For the second IC, the voxel is within the 95th percentile (IC weight = 0.41, | cutoff| = 0.22, range = ± 1.1). For the third IC, the voxel is outside of the 95th percentile (IC weight = 0.02, | cutoff| = 1.22, range = ± 3.8). Therefore, for this voxel, event *A* is true for the second IC, while event *B* is true for the first and second ICs. The marked voxel then has a probability of ½ of being strongly weighted by a noise component, given the three ICs shown. Repeating this calculation for all voxels and all ICs in the analysis yields the Voxel Inclusion Probability Volumes displayed in Figures 4, 5 and Supplementary Figure 6.

Voxel Inclusion Probabilities localize quantitative network-level traits, shared by many ICs, to individual voxels. For example, displayed in Figure 1 are spatial maps for three ICs. Two of the ICs represent nuisance sources in the WM and CSF, while the third spatial map is an ICN centered on visual cortices. The goal of Voxel Inclusion Probabilities is to capture the shared information (informally speaking) of the noise ICs located at the voxel marked by an asterisk. This is more challenging than may be apparent due to the sign and scale indeterminacies in Equation 1, as well as the inclusion of both noise and non-noise ICs in the analysis. The simple procedure, developed formally below, consists of three steps. First, the number of times the voxel is strongly weighted in a spatial map is determined (event *A*, indicated by red or blue segments of the color bars in Figure 1). Second, the number of spatial maps classified as noise is determined (event *B*, based on correlations *r*_*CSF*_ and *r*_*WM*_ shown underneath the spatial maps). Third, conditional probability is applied to quantify what proportion of noise ICs strongly weight the marked voxel (calculated as ½ in this example, as shown on the bottom of the figure). Finally, when this procedure is repeated for all voxels, the result is a spatial map showing the shared features of the noise ICs, such as the prominent third ventricle in the example (Figure 1). The result is a flexible and powerful visualization method that can be used to display arbitrary sets of ICs.

Voxel Inclusion Probabilities are calculated using conditional probability. From basic probability theory, the conditional probability of an event *A* given event *B* is the joint probability of events *A* and *B*, divided by the unconditional probability of event *B*, or Pr{*A*| *B*} = Pr{*A* and *B*}/Pr{*B*}. Formally, let *v* denote a voxel with IC weight *s* in component spatial map **s***_*k*_*, and let P_95_ denote the 95th percentile cutoff from | **s***_*k*_*|. Let event *A* denote the event {| s| > *P*_95_}, indicating that voxel *v* is in the 95th percentile of a spatial map. Event *B* is a criterion or set of criteria on the spatial map, such as a correlation exceeding a cutoff criterion. The Voxel Inclusion Probability for voxel *v* is the conditional probability that *v* is in the 95th percentile of a spatial map, given that the spatial map satisfies criteria *B*, which is calculated as follows:

(3) $$P_{r}\,\left\{\upsilon \,\epsilon \,A|B\right\}=\frac{\#\,\left\{A\cap B\right\}}{\#\,\left\{B\right\}}$$

Equation 3 is calculated for every voxel within the brain. The results can then be displayed as whole-brain spatial maps showing the distribution of heavily weighted voxels satisfying criteria *B*, termed a Voxel Inclusion Probability Volume. Equation 3 is zero for voxels with weak IC weights in all spatial maps meeting criteria *B* and obtains a maximum of one for voxels with large IC weights for every spatial map in *B*.

#### Independent Component Analysis Multi-Scale Network Topology

To compare network topology from ICA to network topology observed using other methods, results from ICA model orders of 2–10 were classified by correlating all IC spatial maps with existing low-dimensional ICN templates (Yeo et al., 2011). Additionally, ICNs were compared to networks resulting from other analytic methods with a similarly low model order, such as hierarchical clustering (Doucet et al., 2011; Gotts et al., 2020) or modularity (Meunier et al., 2009). A specific ICN was considered present in the ICA model if any IC correlated with the appropriate template at *p* < 0.001, with Family-Wise Error (FWE) Rate (FWER) controlled using Bonferroni’s method. Differences in network topology across ICA model orders were assessed by evaluating changes in the maximum correlation with the ICN templates.

To demonstrate that a canonical ICN is contained within the group PCA space, but absent from ICA results at a specific model order, a Snowball ICA (Hu et al., 2020) was performed. In this procedure, at each iteration, the most stable IC was identified using bootstrap stability index *I*_*q*_, then respective back-reconstructed spatial maps were subtracted from subjects’ data. ICA was then repeated as before, including data reduction with PCA, on the subtracted data. Subsequent iterations then identified and removed the most stable IC from the data and repeated ICA. This procedure reliably extracts more ICNs than are initially present in ICA results for a single model order (Hu et al., 2020).

#### Identifying Non-neuronal Source Signals

Despite the use of ICA-FIX to remove non-neuronal signals from the HCP data, residual signals from WM and CSF may remain. To investigate this possibility, all component spatial maps were correlated with WM and CSF tissue probability maps from SPM12^4^ (Ashburner and Friston, 2005). Resulting distributions of correlation coefficients, *r*_*WM*_ and *r*_*CSF*_, were displayed as histograms. To identify components with spatial maps primarily located in the WM or CSF, extremum values for *r*_*WM*_ and *r*_*CSF*_ were chosen based on the tails of each histogram.

To ensure that resulting extremum cutoff values (| *r*_*CSF*_| > 0.2 or | *r*_*WM*_| > 0.2) identified components primarily located in the CSF or WM without inadvertently including gray matter voxels, Voxel Inclusion Probabilities were calculated using Equation 3, with event *A* defined as {| *s*| > *P*_95_}, indicating that the voxel is in the 95th percentile of a spatial map, and event *B* as {| *r*_*CSF*_| > 0.2 or | *r*_*WM*_| > 0.2}. Ideally, the Voxel Inclusion Probability Volumes would encompass primarily CSF and WM voxels with minimal extension into the gray matter, as verified by overlaying onto T1 structural volumes.

#### Identifying Artifactual Source Signals

At high model orders, ICA is potentially prone to artifactual overfitting. The resulting output takes the form of “spikes and bumps,” where the spatial map is composed entirely of single, intense foci limited to a small cluster of voxels and IC weights near zero outside of this cluster (Hyvärinen et al., 1999; Särelä and Vigário, 2003). Overfitting artifacts represent potentially significant confounders in high-dimensional (*K* > 70; Abou−Elseoud et al., 2010) or ultrahigh dimensional ICA (*K* > 200; Iraji et al., 2019), thus impeding analyses at the fine-grained scales of the brain’s multi-scale network topology. Importantly, overfitting ICs are hypothesized to be quantitatively and qualitatively distinct from the focal gray matter ICs resulting from ultrahigh dimensional ICA (Iraji et al., 2019).

To identify potential overfitting artifacts, the use of simple summary statistics on spatial maps was investigated. The maximum IC weight *s*_*max*_ from spatial map **s***_*k*_* was calculated:

(4) $$S_{m\,a\,x}=m\,a\,x_{s\,\epsilon \,s_{k}}\,|S|$$

The size of all clusters was calculated as the number of voxels contained within each cluster above a cluster-determining threshold, the top 95th percentile of the absolute value of all IC weights, denoted *P*_95_. Let Clu denote the set of all voxels encompassed by a single cluster, and let *v* denote a voxel with IC weight *s* in spatial map **s***_*k*_*. Then, the size of each cluster was calculated:

(5) $$C\,l\,u_{s\,i\,z\,e}=\#\,\left\{\upsilon :|s|\geq P_{95},\upsilon \,\epsilon \,C\,l\,u\right\},$$

with the maximum cluster size, denoted *Clu*_*max*_, as the maximum of Equation 5. The mean magnitude of voxels outside of the largest cluster (i.e., the complement of *Clu*_*max*_) was calculated:

(6) $$\mu^{c}=\frac{1}{\#\left\{\upsilon \notin Clu_{m\,a\,x}\right\}}\,\sum_{\upsilon \notin C\,l\,u_{m\,a\,x}}|s|$$

Using these measures, components resulting from overfitting were indicated by a high *s*_*max*_, small *Clu*_*max*_, and μ*^*c*^* near zero. Statistical inference on these measures was carried out using (in order, respectively) order statistics (Shao, 2003), random field theory (Worsley et al., 1996), and permuting voxel values 100 times per IC across all model orders.

Given the large number of subjects and long scanning time in the HCP dataset, we hypothesized that overfitting would be rare for low to intermediate ICA model orders, as reflected by the above measures. We further hypothesized that, when these measures were plotted as scatterplots for all components, resulting clusters of overfitting components would be distinct from known ICNs and could be quickly and easily identified using a simple set of criteria: high *s*_*max*_, small *Clu*_*max*_, and μ*^*c*^* near zero. Visual inspection was used to confirm that ICs meeting these criteria were distinct from focal ICNs reported at high-dimensional or ultra-high-dimensional ICA (Iraji et al., 2019). Lastly, Voxel Inclusion Probabilities were calculated using Equation 3, event *A* as {| *s*| > *P*_95_}, and event *B* as {*s*_*max*_ > 6, and *Clu*_*max*_ < 5,000 voxels, and μ*^*c*^* < 0.035}. The resulting Voxel Inclusion Probability Volume displayed the spatial distribution of components meeting these criteria.

#### Network Subdividing and Bootstrap Instability

As the ICA model order increases, ICA algorithms subdivide larger ICNs, such as the Visual Network that encompasses the primary visual cortex and associated occipitotemporal and occipitoparietal cortices, into their constituent subnetworks, such as the primary and secondary visual subregions. This phenomenon has been referred to as “network splitting” (Abou−Elseoud et al., 2010). However, this term is largely used informally and inaccurately conflates a temporal multi-scale process, that of “splitting,” with a spatial multi-scale topology. To better reflect the underlying spatial multi-scale biology, the neutral term “network subdividing” will subsequently be adopted for the current analysis.

Network subdividing may arise when the number of independent sources in the data is greater than the model order *K*. In this case, ICA can only estimate *K* components, less than the true number of sources (Hyvarinen et al., 2001). The ICA algorithm then necessarily either discards sources or bundles together independent sources into a single component. As *K* is increased, fewer sources are bundled together. Consequently, when spatial maps from different values of *K* are compared, the higher-order model will appear to subdivide a bundled component in the lower-order model (e.g., the Visual Network) into independent sources (e.g., the primary and secondary visual regions).

**FIGURE 2:**
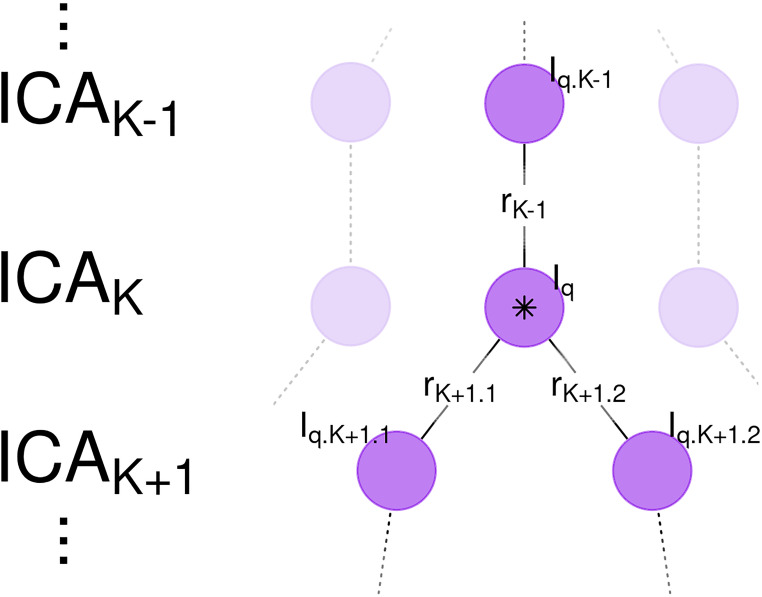
Weighted Average Bootstrap Stability Index and Network Subdividing. Shown are a sequence of independent component analysis (ICA) models with model orders *K*–*1*, *K*, and *K*+*1*. A component in ICA*_*K*_* (central purple circle, marked by star) is highly correlated with a spatial map in ICA*_*K*_*_–_*_1_* and subsequently subdivides into two spatial maps in ICA*_*K*_*_+_*_1_*. Due to network subdividing, the bootstrap stability index *I*_*q*_ for this node is low. However, the nearly identical component in ICA*_*K*_*_–_*_1_* is not affected by network subdividing and is consequently highly stable. Edge weights are correlations between spatial maps *r*_*K*__–_*_1_*, *r*_*K*__+_*_1.1_*, and *r*_*K*__+_*_1.2_*. The Weighted Average Bootstrap Stability Index ($\bar{I_{q}}$) for the marked node is then an average of the bootstrap stability indices for this network, with weights proportional to the correlations (*r*_*K*__–_*_1_*, *r*_*K*_, *r*_*K*__+_*_1.1_*, and *r*_*K*__+_*_1.2_*). $\bar{I_{q}}$ is thus better able to estimate the true bootstrap stability of this component, independent of the effects of network subdividing.

To investigate the effect of network subdividing on bootstrap stability, a weighted average of *I*_*q*_ was calculated. Weights were based on correlation coefficients to the best matching components from previous and subsequent model orders. To support the use of a weighted average, consider the ideal case of a component whose spatial map is unchanged between ICA*_*K*_*_–_*_1_* and ICA*_*K*_*, then is evenly divided into two subnetworks in ICA*_*K*_*_+_*_1_* (Figure 2). In this case, averaging together all of the bootstrap stability indices *I*_*q*_, from each instance of the component from each ICA model, would provide a better estimate of the stability of this component, without the confounding effects of component subdividing on the stability index. The weighted average bootstrap stability index $\bar{I_{q}}$ generalizes this concept to non-ideal cases by using correlation coefficients between associated spatial maps as weights.

For a component from ICA*_*K*_* with bootstrap stability index *I*_*q*_, correlations were calculated between all spatial maps from the previous and subsequent models, denoted ICA*_*K*_*_–_*_1_* and ICA*_*K*_*_+_*_1_*. Let *r*_*K*__–_*_1_* denote the highest magnitude correlation coefficient from ICA*_*K*_*_–_*_1_*, with associated bootstrap stability metric *I*_*q.K*__–_*_1_*. Since ICNs potentially subdivide into two subnetworks, let *r*_*K*__+_*_1.1_* and *r*_*K*__+_*_1.2_* denote the two highest magnitudes from ICA*_*K*_*_+_*_1_* with associated bootstrap stabilities *I*_*q.K*__+_*_1.1_* and *I*_*q.K*__+_*_1.2_*. The weighted average bootstrap stability index for a component is then calculated:

(7) $$\bar{I_{q}}=\frac{\begin{array}{c} |r_{K-1}|*I_{q.K-1}+1*I_{q}+|r_{K+1.1}|*I_{q.K+1.1}\\ +|r_{K+1.2}|*I_{q.K+1.2} \end{array}}{|r_{K-1}|+1+|r_{K+1.1}|+|r_{K+1.2}|}.$$

(See Figure 2 for a graphical display of this calculation and the relationship between each variable.) Each term in the numerator is an unweighted bootstrap stability index *I*_*q*_ calculated with Equation 2, weighted by the correlation between the spatial maps. In order in Equation 7, these are the best matching IC from the previous model ICA*_*K*_*_–_*_1_*, the current component (with a correlation coefficient of one since a random variable always correlates perfectly with itself), and the two best matches from the subsequent model ICA*_*K*_*_+_*_1_*. The denominator is a normalization factor to ensure the weights sum to one. Compared to unweighted *I*_*q*_, weighted average $\bar{I_{q}}$ is expected to minimize the effects of network subdividing on bootstrap stability, since an unstable component with a low unweighted *I*_*q*_ will correlate highly with more stable spatial maps from preceding and subsequent models ICA*_*K*_*_–_*_1_* and ICA*_*K*_*_+_*_1_*. Statistical inference on $\bar{I_{q}}$ was carried out by permuting cluster membership 1,000 times per ICA model order.

## Results

### Independent Component Analysis Network Topology at Low Model Order

At ICA model order *K* less than 10, component spatial maps closely matched previously reported ICNs (Yeo et al., 2011). Spatial maps closely matched templates for the FPCN, DMN, Somatomotor Network (SMN), Dorsal Attention Network (DAN), Ventral Attention Network (VAN), and Visual Network, as indicated by a high maximum correlation with templates for each of these ICNs (Table 1). No spatial map correlated with the Limbic Network template, at any low model order examined (| *r*| < 0.08, *p* > 0.001 uncorrected in all cases). As *K* increased, many networks were subdivided into subnetworks. At ICA_5_, the Visual Network is subdivided into Central and Peripheral subnetworks. At ICA_8_, the FPCN is subdivided into Right and Left Executive Control Networks (RECN and LECN, respectively). At ICA_10_, the SMN subdivided into ventral and dorsal subnetworks, corresponding to face and body subdivisions of the somatomotor system, respectively (Yeo et al., 2011).

**TABLE 1:** Maximum Correlations Between Intrinsic Connectivity Network (ICN) Templates and independent component analysis (ICA) Spatial Maps by Model Order.

ICA model order (*K*):	DMN	DAN	FPCN	Limbic	SMN	VAN	Visual
2	0.08	0.37**	0.48**	−0.08	0.12	0.15*	0.47**
3	0.42**	0.41**	0.47**	0	−0.09	0.17*	0.74**
4	0.47**	0.41**	0.47**	−0.02	0.56**	0.17*	0.76**
5	0.53**	0.36**	0.5**	−0.03	0.62**	0.16*	0.72**
6	0.52**	0.55**	0.44**	−0.02	0.62**	0.25**	0.5**
7	0.4**	0.53**	0.41**	0	0.65**	0.3**	0.54**
8	0.42**	0.54**	0.43**	0	0.66**	0.45**	0.5**
9	0.45**	0.47**	0.36**	0.03	0.66**	0.47**	0.43**
10	0.47**	0.43**	0.35**	0.02	0.37**	0.41**	0.43**

All spatial maps from a given ICA model were correlated with a set of seven ICN templates (Yeo et al., 2011), including the Default Mode Network (DMN), Dorsal Attention Network (DAN), Frontoparietal Control Network (FPCN), Limbic Network, Somatomotor Network (SMN), Ventral Attention Network (VAN), and Visual Network. For each ICN, the maximum correlation is displayed for each ICA*_*K*_*. Different ICA*_*K*_* show a different set of ICNs, and a sudden increase of the maximum correlation for an ICN template indicates the appearance of that network within the ICA model. FWE, Family-Wise Error. *p < 0.001, FWE-corrected. **p < 10^–5^, FWE-corrected.

**FIGURE 3:**
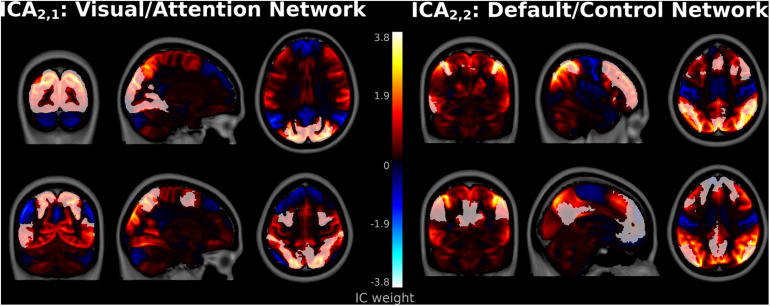
Spatial Maps at independent component analysis (ICA)_2_. Component ICA_2,1_ encompassed Visual and Dorsal Attention Network regions (left top and bottom, respectively). Component ICA_2,2_ encompassed Frontoparietal Control Network regions (top right), as well as regions more associated with the Default Mode Network such as the lateral inferior parietal lobes, precuneus, and posterior cingulate cortices (bottom right). Frontoparietal Control and Default Mode Network templates from Yeo et al. (2011).

Several spatial maps notably differed from previously reported ICA networks, consistent with hypothesized meta-networks. At ICA_2_, a spatial map, denoted ICA_2,1_, encompassed both the occipital lobe visual system as well as frontoparietal association regions (Figure 3). This network correlated highly with both Visual Network (*r* = 0.47, *t*_1,082_ = 17.5, *p* < 10^–5^ FWE-corrected) and DAN templates (*r* = 0.37, *t*_1,082_ = 13.1, *p* < 10^–5^ FWE-corrected). Correlations between ICA_2,1_ and the DMN template were strongly negative (*r* = −0.38, *t*_1,082_ = −13.5, *p* < 10^–5^ FWE-corrected), consistent with anti-correlations previously observed between DMN and DAN (Fox et al., 2005).

The second spatial map in ICA_2_, denoted ICA_2,2_, primarily encompassed dorsolateral prefrontal and parietal control regions and strongly matched the FPCN template (*r* = 0.48, *t*_1,082_ = 18.0, *p* < 10^–5^). Interestingly, major positive clusters in ICA2,2 encompassed adjacent regions more commonly associated with the DMN (Raichle et al., 2001; Greicius et al., 2003), such as the lateral inferior parietal lobes, precuneus, and posterior cingulate cortices (Figure 3). However, the correlation between ICA_2,2_ and the DMN template was weak (*r* = 0.08, *t*_1,082_ = 2.4, *p* = 0.012 uncorrected, *p* > 0.05 FWE-corrected). This suggests that this network is more accurately classified primarily as an FPCN that partially encompasses the DMN.

Similar extensive networks that encompass more than one canonical ICN template have not been widely reported in ICA. However, these results are consistent with the task-positive and -negative networks from hierarchical clustering analyses (Meunier et al., 2009; Doucet et al., 2011; Lee et al., 2012; Gotts et al., 2020). At the highest levels of the connectivity dendrogram, hierarchical clustering initially separates the brain into Visual/Attention and Default/Control meta-networks. The spatial maps for ICA_2,1_ and ICA_2,2_ suggest that these more extensive networks are not artifactual or unique to hierarchical clustering but are reproducible with ICA.

In contrast to hierarchical clustering analyses, ICA spatial maps were not always neatly subdivided into nested subnetworks as *K* increased. Instead, many prominent networks were initially absent from lower-order models, then appeared seemingly *de novo* as *K* increased. For example, at ICA_2_ and ICA_3_, no spatial map correlated substantially with the SMN template (Table 1; *r* < 0.15, *p* > 0.05 FWE-corrected, in all cases). Subsequently, at ICA_4_, a spatial map encompassing the bilateral primary somatosensory cortices strongly matched this template (*r* = 0.56, *t*_1,082_ = 22.2, *p* < 10^–5^ FWE-corrected). Other networks present in ICA_4_, the FPCN, Visual Network, and DMN, were all relatively unchanged from lower model orders, as indicated by a relatively constant maximum correlation in Table 1 (Fischer’s *z* < 1.06, *p* > 0.05 uncorrected). In this case, the SMN was entirely absent from *K* less than 4, appeared fully formed at ICA_4_, *de novo*, and appeared without precedent at lower orders. This result suggests that a canonical ICN may be present within the data, yet may not appear in the results. Since the number of ICs estimated is determined by ICA model order, this is likely a consequence of limitations of the ICA algorithm.

To demonstrate that the SMN is contained within the group PCA space at low dimensionality, a snowball ICA was performed (Hu et al., 2020). At each iteration, the most stable IC was subtracted from all subjects’ data. ICA_2_ was repeated as before, including data reduction with PCA, and two more components extracted. After three iterations, this resulted in an IC strongly resembling the SMN (Supplementary Figure 2). This IC was centered on the bilateral pre-central gyri and strongly correlated with the SMN template (*r* = 0.51, *t*_1,082_ = 19.5, *p* < 10^–5^ FWE-corrected). This suggests that the absence of SMN at low dimensions results from the limitations of the ICA algorithm, specifically the maximum number of components extracted.

Alternatively, the absence of the SMN at ICA_2_ and ICA_3_ may have been due to the limited span of the group PCA space at these model orders (dimensions 2 and 3, respectively). Increasing the dimension of the group PCA space to 30 resulted in an entirely new set of components, unrelated to results from other ICA models (Supplementary Figure 2). The resulting spatial maps were centered on CSF and parenchyma, rather than gray matter, and did not include the SMN. This suggests that different sets of components can be estimated by the same ICA model order, depending on the parameters of the model. Furthermore, this suggests that the absence of the SMN at low model orders was not related to the dimension of the PCA space.

Network subdividing did not always lead to binary parcellations of a network into disjoint subnetworks. For example, a Central Visual Network appeared at ICA_5_ [*r* = 0.51 with a template from 17-network parcellation of Yeo et al. (2011), *t*_1,082_ = 19.5, *p* < 10^–5^ FWE-corrected], partially overlapping with the more extensive Visual Network whose spatial map was unchanged from ICA_4_. These networks coexisted over several ICA model orders, with a gradual rearrangement of the Visual Network into a distinctly Peripheral Visual Network by ICA_9_ (Supplementary Figure 3).

Surprisingly, network subdividing may result in a subnetwork being temporarily absent from subsequent ICA models, even while other subnetworks remain. As an example, right and left dorsolateral prefrontal cortices were encompassed within the bilateral FPCN at low *K*. At higher *K*, this network was subdivided into lateralized RECN and LECN. In the current analysis, ICA_7_ included a symmetric and bilateral spatial map corresponding to the FPCN. At ICA_8_, this network subdivided into subnetworks, with only the RECN appearing in ICA_8_. Left-lateralized frontoparietal control regions were conspicuously absent from the component spatial maps of ICA_8_ (Supplementary Figure 3). Although a network matching the left-lateralized language regions of the DMN template was present at ICA_8_, these regions did not overlap with the adjacent regions encompassed by the FPCN template. Subsequently, at ICA_9_, a spatial map corresponding to the LECN then reappeared in the ICA model (Supplementary Figure 3). In this example, an entire canonical ICN, the LECN, was briefly and unexpectedly absent from the sequence of ICA models and temporarily disappeared from the results.

Spatial maps at the most widely used ICA model order, ICA_20_ (Smith et al., 2009), largely matched commonly reported ICNs and subnetworks (Supplementary Figure 4; Yeo et al., 2011; Shirer et al., 2012). The DMN was subdivided into anterior and posterior ICs, matching previous reports (Abou−Elseoud et al., 2010; Allen et al., 2011). The SMN was subdivided into dorsal and ventral subnetworks, while the Primary Visual Network was subdivided into central and peripheral subnetworks (Yeo et al., 2011). RECN and LECN subdivisions of the FPCN coexisted with an IC encompassing dorsolateral prefrontal cortices bilaterally. While a non-template ICN encompassed the cerebellum, no other IC substantially encompassed other subcortical regions in the basal ganglia or cerebellum.

### Identifying Nuisance Sources

**FIGURE 4:**
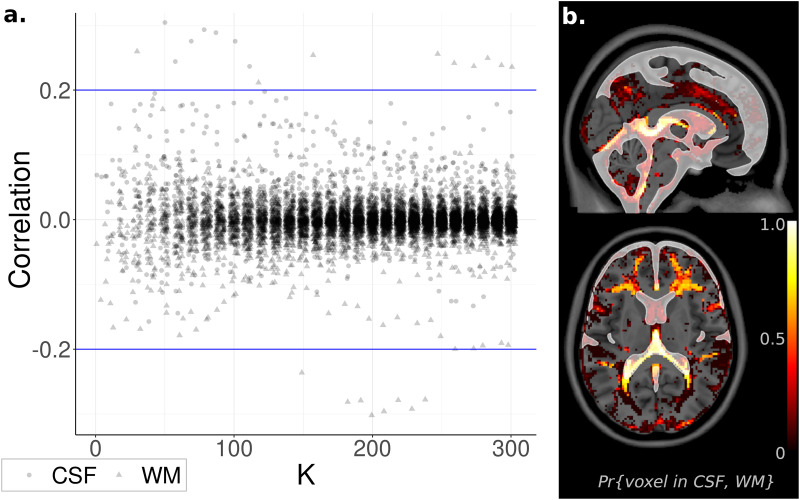
Relationship to Noise Sources. **(a)** Component spatial maps correlated with white matter (WM) and cerebrospinal fluid (CSF) tissue probability maps and displayed with independent component analysis (ICA) model order *K*. Almost all correlations were near zero and unrelated to *K*. **(b).** Voxel Inclusion Probability Volumes (see Equation 3) for spatial maps with either correlation coefficient | *r*_*WM*_| or | *r*_*CSF*_| greater than 0.2. Spatial maps exceeding this cutoff were predominantly located in WM or CSF, minimally overlapping with gray matter.

Nuisance signals located within the CSF and WM were removed using ICA-FIX (Smith et al., 2013). Despite this precaution, residual noise sources may remain in the data and become prominent as *K* increases. Indeed, at *K* as low as ICA_30_, a component featuring a substantial correlation with the WM tissue probability map (*r* = 0.26, *t*_1,082_ = 8.9, *p* < 10^–5^ FWE-corrected) was observed, with a similar result for the CSF at *K* as low as ICA_50_ (*r* = 0.30, *t*_1,082_ = 10.3, *p* < 10^–5^ FWE-corrected). However, almost all ICs were uncorrelated with CSF and WM (| *r*| < 0.1, | *t*_1082_| < 3.3, *p* > 0.001 uncorrected), with no appreciable change as *K* increased (Figure 4). Based on these results, WM and CSF components appear to be rare, but still present, even after removal of noise signals with ICA-FIX.

At lower ICA model orders (*K* < 30), WM and CSF components can be identified by manual inspection. However, at higher model orders, manual inspection becomes infeasible due to the increasingly large number of components. To facilitate the use of high-order ICA, a method of automatically identifying noise signals is essential. Based on the above results, a correlation of | *r*| > 0.2 is a promising candidate guideline, corresponding to the 0.1st and 99.9th sample percentiles across all values of *K*. The spatial distribution within the brain of ICs exceeding this cutoff, displayed as a Voxel Inclusion Probability Volume (see section “Materials and Methods”), indicated that this guideline captured spatial maps that were heavily weighted toward WM and CSF, with minimal to no overlap with gray matter (Figure 4). These results suggest that these nuisance signals can be automatically identified and removed from high-order ICA, without inadvertently discarding neuronal sources.

### Identifying Source Components Resulting From Model Overfitting

**FIGURE 5:**
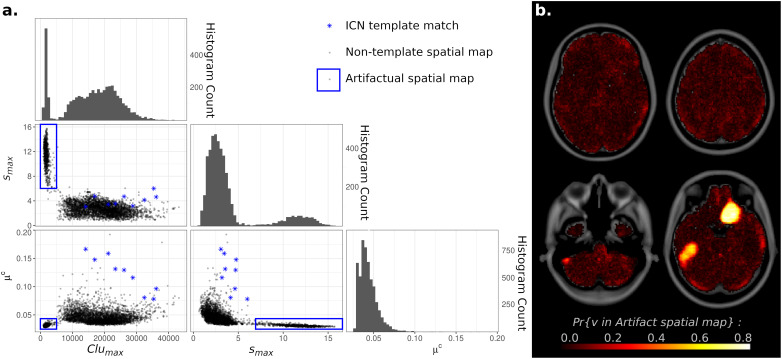
**(A)** Histograms and Scatterplots of Maximum Intensity (*s*_*max*_), Largest Cluster Size (*Clu*_*max*_), and Average independent component analysis (ICA) Component (IC) Weight Outside of the Largest Cluster (μ*^*c*^*) for all components. Histograms of all three measures showed bimodal distributions. Scatterplots demonstrated that components cluster into two disjoint clusters. Components matching known Intrinsic Connectivity Networks [ICNs (Yeo et al., 2011)] were all located within one cluster (blue stars), while components with overfitting “spike and bump” characteristics (high *s*_*max*_, small *Clu*_*max*_, and μ*^*c*^* near zero) were located in the other scatterplot cluster (blue circle). **(B)** Voxel Inclusion Probability Volume (see Equation 3) showing the spatial distribution of artifactual spatial maps (blue square on left). Components resulting from artifactual overfitting were largely spread diffusely throughout all tissue types within the brain, consistent with a non-neuronal origin. Two prominent supratentorial spatial clusters were observed in the right olfactory and left inferior temporal cortices.

At high model orders, ICA may overfit the data as well, leading to artifactual “spike and bump” components (Hyvärinen et al., 1999; Särelä and Vigário, 2003). These non-neuronal source signals consist of intensely focal spatial maps, with high IC weights localized to a relatively small cluster of voxels, and IC weights near zero for all voxels outside this cluster. To investigate this possibility, metrics were developed to measure maximum intensity, largest cluster size, and average weight outside of the largest cluster (denoted *s*_*max*_, *Clu*_*max*_, and μ*^*c*^*, respectively) and applied to all spatial maps across all ICA model orders. For each metric, histograms showed a clear bimodal distribution (Figure 5). Scatterplots of these measures separated components into two disjoint clusters, with spatial maps corresponding to known ICNs all associated with one cluster and spatial maps with “spike and bump” characteristics (high *s*_*max*_, small *Clu*_*max*_, and μ*^*c*^* near zero) associated with the second scatterplot cluster. Based on Figure 5, “spike and bump” components can be automatically identified using cutoff criteria of *s*_*max*_ > 6, *Clu*_*max*_ < 5,000 voxels (or 40 cm^3^ by volume), and μ*^*c*^* < 0.035.

Individually, spatial maps satisfying any single one of the above criteria may occur by chance (*p* = 0.00051 for *s*_*max*_ > 6, *p* = 0.951 for *Clu*_*max*_ < 5,000 voxels, *p* = 0.093 for μ*^*c*^* < 0.035). However, the combination of all three criteria co-occurring in a spatial map is highly unlikely (*p* = 4.45 × 10_–_^5^ for the intersection of all three events). Visual inspection of spatial maps flagged with these criteria strongly suggested non-neuronal, artifactual sources (see Supplementary Figure 5 for examples). All spatial maps flagged by these criteria consisted of isolated unilateral, narrow, elongated hyperfocal spikes. Foci were rarely more than three voxels in width, extending for approximately 10 voxels laterally, in the direction of slice acquisition. The “spikes and bumps” characterization is thus accurate.

The overall concentrated spatial distributions of “spike and bump” components within the brain were investigated by calculating Voxel Inclusion Probabilities for the above criteria. Results were then displayed as a whole-brain spatial map. Components satisfying these criteria were largely spread diffusely throughout all tissue types within the brain, consistent with a non-neuronal origin (Figure 5). However, two prominent supratentorial spatial clusters were observed, in the right olfactory and left inferior temporal cortices. Given the susceptibility of fMRI to magnetic field inhomogeneities in supratentorial regions (Huettel et al., 2009), these two spatial clusters likely represent the artifactual signal, although either from a different origin or intermixed with the hypothesized “spikes and bumps” overfitting-related artifacts.

Lastly, the specificity of the above cutoff guidelines was investigated. The spatial distribution of all non-nuisance and non-artifactual components that did not meet the above criteria was displayed as a Voxel Inclusion Probability volume. As expected, the remaining non-nuisance and non-artifactual signals were almost exclusively located within the gray matter, encompassing the entirety of the cortex and subcortical regions (Supplementary Figure 6).

Summarizing the above results, nuisance and artifactual signals were identified from ICA spatial maps using a set of simple criteria: (1) a correlation magnitude with a nuisance tissue probability map > 0.2 and (2) a maximum IC weight > 6, and a maximum cluster size > 5,000 voxels with isotropic 2-mm sides, corresponding to 40 cm^3^ total volume, and an average of IC weights of all voxels outside the largest cluster < 0.035. In the current analysis, criterion 1 was used to identify WM and CSF components, while criterion 2 was used to identify overfitting artifacts, with all likely overfitting spatial maps required to satisfy all three individual conditions. After discarding components meeting these exclusion criteria, the remaining components should largely represent non-artifactual sources that capture signals located within the gray matter.

### Bootstrap Stability Across Independent Component Analysis Model Orders

Bootstrap resampling is a powerful method of estimating reproducibility by recalculating a statistic using a subset of subjects and comparing the distribution of the resampled statistic to the statistic calculated using the full set of subjects (Shao, 2003). In ICA, bootstrap resampling of component spatial maps is known as ICASSO (Himberg et al., 2004) and the component stability index measure is denoted *I*_*q*_. When applied to fMRI, an *I*_*q*_ averaged over all components in a single ICA*_*K*_* greater than 0.9 is generally considered an acceptable level of reproducibility. Previous applications of ICASSO, using data sets with fewer subjects and shorter scan lengths, have suggested an ICA model order of ICA_70_ or less for general use (Kiviniemi et al., 2009; Abou−Elseoud et al., 2010).

The large number of subjects (*n* > 1,000) and time points (*t* = 1,200) in the HCP database may facilitate higher-order ICA models with an average *I*_*q*_ greater than 0.9 for a given model order *K*. To investigate this possibility, *I*_*q*_ was compared to the full range of *K* in the current investigation, from 2 to 300. Furthermore, since statistics such as sample average may be influenced by atypical outliers, and thus not reflective of the stability of most components, sample quantiles for each *K* were used as a summary statistic.

**FIGURE 6:**
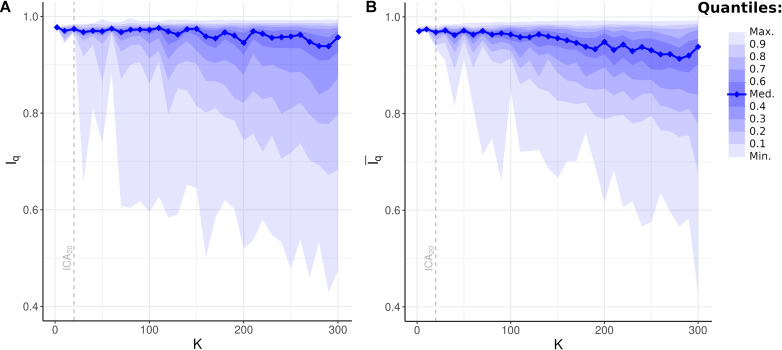
Bootstrap Stability Indices *I*_*q*_ and independent component analysis (ICA) Model Order *K*. **(A)** Unweighted bootstrap stability index *I*_*q*_, plotted as sample quantiles in increments of 10 (e.g., bottom lightest shade encompasses minimum to top 10th quantile of *I*_*q*_ at given model order), with the solid blue line showing median *I*_*q*_. Upper quantiles remained highly stable even at high *K*, while lower quantiles gradually decreased. Dashed line: quantiles at *K* = 20 (i.e., ICA_20_). **(B)** Weighted average bootstrap stability index $\bar{I_{q}}$ plotted as sample quantiles and median. Compared to unweighted *I*_*q*_, weighted average $\bar{I_{q}}$ demonstrated fewer outliers in the lowest quantiles resulting from network subdividing. Dashed line: quantiles at ICA_20_.

All *I*_*q*_ values for all ICs across all model orders were statistically significant (*p* < 0.001, FWE-corrected). Surprisingly, median *I*_*q*_ and the upper sample quantiles remained virtually unchanged even as model order increased to ICA_300_ (Figure 6; median *I*_*q*_ > 0.95 for *K* = 2, 10,…,300). Even at ultra-high-dimensional ICA, defined as ICA_200_ or greater, the majority of components were highly stable under bootstrap resampling. Also unexpected was the appearance of unstable components with *K* as low as ICA_30_ (minimum *I*_*q*_ = 0.66). Thus, even low-order ICA on a large, high-quality dataset may include unstable components in the analysis. A slow and gradual decrease in the lower quantiles of *I*_*q*_ was notable, beginning at approximately ICA_100_. When averaged across all components in a given model, mean *I*_*q*_ was above 0.9 up until ICA_270_ and remained at or above 0.88 until at least ICA_300_. These results suggest that unstable components are likely unavoidable, even at the low ICA model orders commonly used in fMRI. Furthermore, and despite this, these results also suggest that the majority of components in ultra-high-dimensional ICA, with a model order up to 300, are highly stable and that the average *I*_*q*_ remains very near the accepted standard of reproducibility.

### Bootstrap Stability and Network Subdividing

Network subdividing refers to when a canonical ICN, such as the FPCN, appears as a single network at lower *K* and subsequently subdivides into subnetworks, such as the LECN and RECN in the case of the FPCN, as *K* increases. This concept is equivalent to hierarchical modularity in graph theory, where smaller submodules are nested inside larger networks across multiple scales (Meunier et al., 2009). Although this phenomenon argues against the perhaps more intuitive view of ICNs as indivisible fundamental processing entities within the brain, it is a commonly observed feature in network analyses and may represent a fundamental topology of complex networks in general and neurobiology specifically (Churchland and Sejnowski, 1988; Meunier et al., 2009).

Network subdividing is dependent upon data quality, including the number of subjects. The lowest bootstrap stability indices for a given *K* in Figure 6A may thus represent components that are on the verge of being subdivided into subnetworks. In this case, both the larger network and its subnetworks appear in the bootstrap replicates of a spatial map, thus obfuscating their similarity and decreasing the measured bootstrap stability index for a single ICA*_*K*_*. To investigate this possibility, weighted average bootstrap stability indices $\bar{I_{q}}$ were calculated for all components in all model orders. For each spatial map, the likely incidence of this component in the previous and subsequent models was determined based on the highest correlation values. $\bar{I_{q}}$ is then the weighted average of the associated bootstrap stability indices for the incidence of the same components, weighted by the correlation coefficients. Components likely resulting from overfitting (see above) were excluded from the calculation.

All values of $\bar{I_{q}}$ for all ICs across all model orders were statistically significant (*p* < 0.001, FWE-corrected). Compared to unweighted bootstrap stability indices (Figure 6A), $\bar{I_{q}}$ increases measured stability indices of the lower sample quantiles, especially the minimum $\bar{I_{q}}$, with minimal effect on upper quantiles (Figure 6B). These results suggest that network subdividing, occurring in specific components at select ICA*_*K*_*, contributes to measured bootstrap stability measures. However, the continued presence of low values of $\bar{I_{q}}$ suggests additional sources of instability in the ICA model, beyond the ability of this statistic to correct for.

## Discussion

In the current investigation, ICA, when applied to a high-quality dataset such as the HCP database, was found to be capable of reliably and reproducibly identifying the full range of network topology necessary for multi-scale processing analyses. At the lowest model order, ICA_2_, results corroborated the combined Visual/Attention and Default/Control meta-networks observed in hierarchical clustering analyses (Meunier et al., 2009; Doucet et al., 2011; Gotts et al., 2020). These large-scale networks are a combination of two canonical ICNs (Yeo et al., 2011): the Visual and Dorsal Attention Networks and the DMN and FPCN. They represent the organization of connectivity within the brain at the largest known scales and have not previously been reported using ICA. Consistent with the task-positive meta-network in hierarchical clustering, the Visual/Attention Network in ICA was a direct combination of the major features of both Visual and Dorsal Attention Networks. However, the Default/Control Network in ICA differed in minor respects from previous results. In hierarchical clustering analyses, the task-negative meta-network is a direct combination of the major features of its constituent subnetworks, encompassing both to an equal extent. However, in the current ICA, this network appeared to be largely an executive control network with atypical features, such as clusters in the precuneus and posterior cingulate cortex, more strongly associated with the DMN.

Artifactual and nuisance sources were still present in some ICA models, despite denoising with ICA-FIX (Smith et al., 2013). This finding is consistent with previously observed limitations of current fMRI denoising strategies (Parkes et al., 2018). Application of ICA-FIX reduced, but did not eliminate, these sources in the data. At all model orders investigated, artifactual and nuisance components comprised a minority of ICA components. Importantly, the overfitting artifacts identified by the criteria given above are distinct from the focal, locally compact gray matter ICNs resulting in ultra-high-dimensional ICA (Iraji et al., 2019). The identification criteria detailed above can be used to automatically identify and potentially remove these non-neuronal sources, without requiring manually-intensive visual inspection of all components at high-dimensional and ultra-high-dimensional ICA. These criteria are likely overly conservative when used to identify noise and artifactual components but are specific to non-neuronal signals and are unlikely to inadvertently misclassify gray matter sources as artifactual or noise signals.

Lastly, the reproducibility of all ICA models was high, even at ultrahigh dimensionality, as demonstrated by the bootstrap stability indices. Indeed, the very high and unchanging median bootstrap stability across all model orders was unexpected. In contrast to ICA, in clustering algorithms, bootstrap instability was evident at a very low model order of *K* = 4 and rapidly increased thereafter (Yeo et al., 2011). Network subdividing likely decreased measured bootstrap stability indices of lower quantiles, as shown by the comparison of unweighted and weighted average stability indices (Figures 6A,B, respectively). However, this likely reflected the instability of the exact ICA*_*K*_* where the subdivision occurred, rather than instability in the resulting spatial maps or underlying biology.

Network subdividing is arguably an inevitable consequence of multi-scale processing, perhaps even its cardinal feature (Simon, 1962; Meunier et al., 2009). Under this network topology, the resulting covariance structure within the brain will vary across multiple scales, resulting in a varying network structure detected by ICA. In this view, the apparent instability of reported ICA networks, commonly resulting from analyzing a single ICA*_*K*_*, reflects the inadequacy of any single ICA model to fully capture the richness of processing occurring within the brain.

Importantly and reassuringly, the most widely used ICA model order, ICA_20_ (Smith et al., 2009), favored very well in the above analysis. Unlike at ICA_10_ or less, all major ICNs or their known subnetworks were present in ICA_20_ spatial maps. Furthermore, bootstrap stability at this model order was high for all components, even for the minimum bootstrap stability index. In contrast, at subsequent model orders, at least one IC spatial map was unstable, as indicated by the sharp decrease in minimum *I*_*q*_ evident in Figure 6A starting at ICA_30_. However, in the broader context of multi-scale network topology, the above results suggest that ICA_20_ is one of many possible scales at which to investigate connectivity. In this model of neural processing, no ICA model order is inherently superior or inferior to any other, provided care is taken to identify non-neuronal sources, but a deeper understanding of the brain’s network organization can be gained by focusing on more than a single scale of inherently multi-scale network topology (Betzel and Bassett, 2017).

The current approach can be implemented by analyzing more than one ICA decomposition of the data. This can be performed by systematically and sequentially increasing ICA across a range of model orders, as in the current study, or by analyzing multiple ICA decompositions of the HCP dataset (Van Essen et al., 2013). The HCP S1200 release^5^ contains ICA decompositions with model orders of 15, 25, 50, 100, 200, and 300 components. However, this set of ICA results is incomplete. The current approach complemented and extended the HCP S1200 release, by showing the meta-networks present in low-dimensional ICA beyond those included in the S1200 ICA decompositions. Additionally, the above results suggested that noise and nuisance signals are present in the S1200 data in high dimensions, even after denoising with ICA-FIX. However, these nuisance and artifactual sources can be identified and removed by applying the quantitative criteria outlined above to the IC spatial maps, without inadvertently discarding signals located within gray matter.

This analysis featured limitations. The term “multi-scale” does not have a single definition (Betzel and Bassett, 2017). It can refer to multiple topological scales, where network nodes are analyzed in the context of a hierarchical topology (Doucet et al., 2011; Betzel and Bassett, 2017; Iraji et al., 2021). This type of multi-scale organization can be investigated with a single experimental modality capable of investigating the levels of a hierarchy, such as fMRI. Alternatively, multi-scale organization can refer to the interrelated domains of cognitive neuroscience (van den Heuvel et al., 2019). This type of multi-scale analysis encompasses microscopic (e.g., genes and cells), mesoscopic (cytoarchitecture), and macroscopic (connectivity, systems neuroscience) scales. This type of multi-scale analysis is beyond the scope of the current investigation. Importantly, both definitions are equally valid, and both share a very similar conceptual approach to understanding the brain (Park and Friston, 2013).

The spatial resolution of fMRI prevents the investigation of microscopic scales. The current analysis was limited to features larger than a millimeter. Notably, networks encompassed by this scale range in size from millimeters (voxels), centimeters (specialized cortical regions), and decimeters (large-scale networks). In addition to these relatively large scales, neural networks exist at smaller scales. These include mesoscopic and microscopic scales not observable with fMRI (van den Heuvel et al., 2019). The inclusion of network information from these scales would generate a fuller, richer model of network topology.

The weighted bootstrap stability index calculation in Equation 7 assumes that an IC will subdivide into two subnetworks, rather than three or more. Furthermore, it only uses stability information from the immediately preceding and subsequent ICA models. These *a priori* assumptions were consistent with known examples of network subdivision, such as the FPCN into RECN and LECN. However, the analysis suggests that network subdividing can result in ICNs that are temporarily absent from the sequence of ICA models, such as the LECN. Future investigations will extend the weighted bootstrap stability index, including more ICs from each level and more levels of the hierarchy.

In conclusion, ICA results were very stable even at high and ultrahigh dimensionalities. While nuisance and artifact sources were present even in the high-quality dataset used here, these sources represented a small minority of all component spatial maps at any ICA model order. They were easily identified using relatively simple criteria calculated from spatial maps. At very low dimensionalities, ICA resulted in spatial maps consisting of meta-networks, such as the Visual/Attention and Default/Control Networks. Lastly, the above results are consistent with a multi-scale network topology, where the brain processes information in networks ranging from the very small to the very large. By varying the model order, ICA may be able to reliably identify the richness of neural processing known to occur across multiple spatial scales.

## Data Availability Statement

Publicly available datasets were analyzed in this study. This data can be found here: Data from healthy participants in the HCP S1200 release (https://www.humanconnectome.org) were included in the current investigation. Individual resting state scans from the HCP minimal pre-processing pipeline were downloaded *via* Amazon Web Services.

## Ethics Statement

The studies involving human participants were reviewed and approved by Colorado Multiple Institutional Review Board. The patients/participants provided their written informed consent to participate in this study.

## Author Contributions

KW designed and carried out analysis and developed novel applied techniques (Voxel Inclusion Probabilities and Weighted Average Bootstrap Stability). EK mathematical advisor for analysis, feedback, and editing of manuscript. KL worked extensively on revising manuscript, editing, and feedback of manuscript. BS gave good feedback on improving manuscript. JT gave good feedback on improving manuscript and supervised all work. All authors contributed to the article and approved the submitted version.

## Conflict of Interest

The authors declare that the research was conducted in the absence of any commercial or financial relationships that could be construed as a potential conflict of interest.

## Footnotes

1 https://ida.loni.usc.edu/login.jsp

2 https://www.humanconnectome.org

3 https://trendscenter.org/software/gift/

4 https://www.fil.ion.ucl.ac.uk/spm/software/spm12

5 https://www.humanconnectome.org/study/hcp-young-adult
