# Supplementary figures and images for: Stable Meta-Networks, Noise, and Artifacts in the Human Connectome: Low- to High-Dimensional Independent Components Analysis as a Hierarchy of Intrinsic Connectivity Networks
Source: Front Neurosci. 2021 May 6;15:625737. doi: 10.3389/fnins.2021.625737 (PMC8134552; doi:10.3389/fnins.2021.625737)

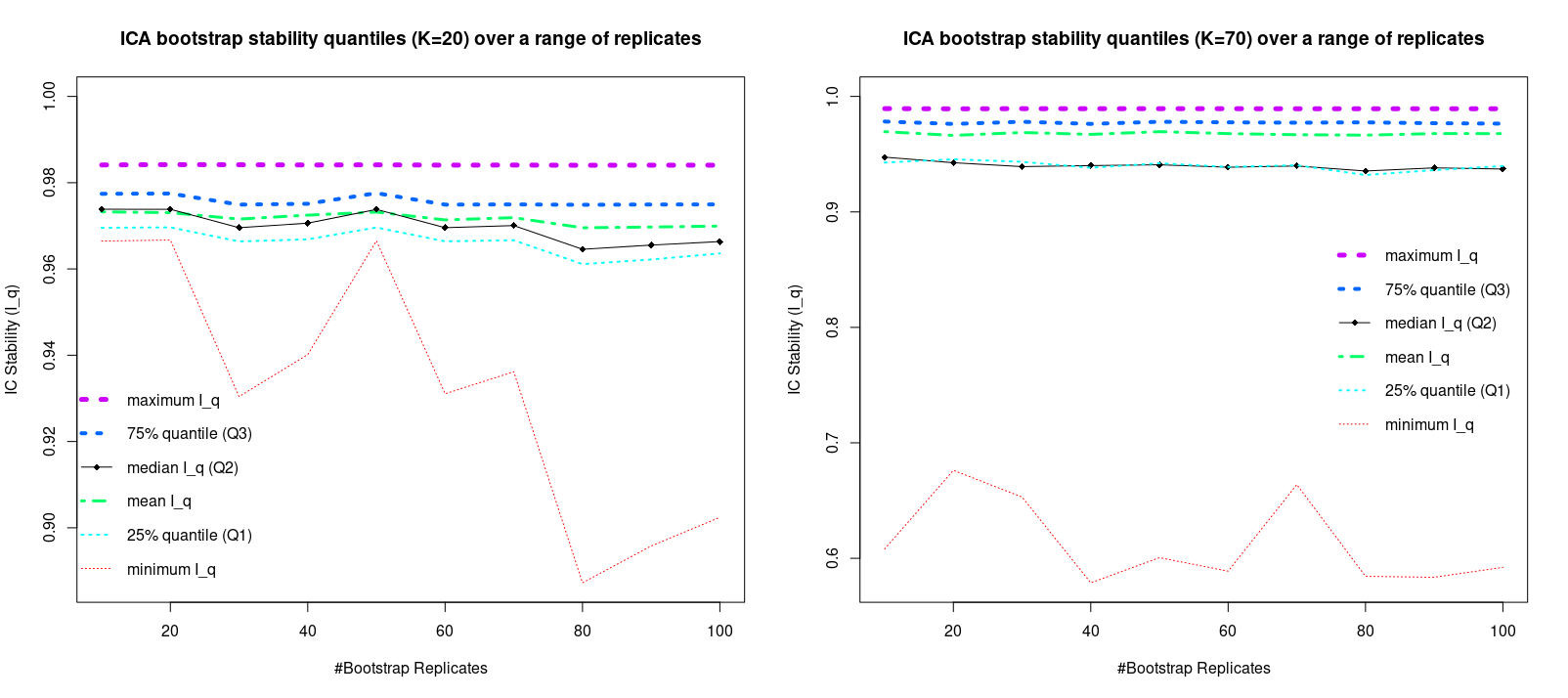

Supplement: Supplementary file 1 [file Image_1.TIFF]

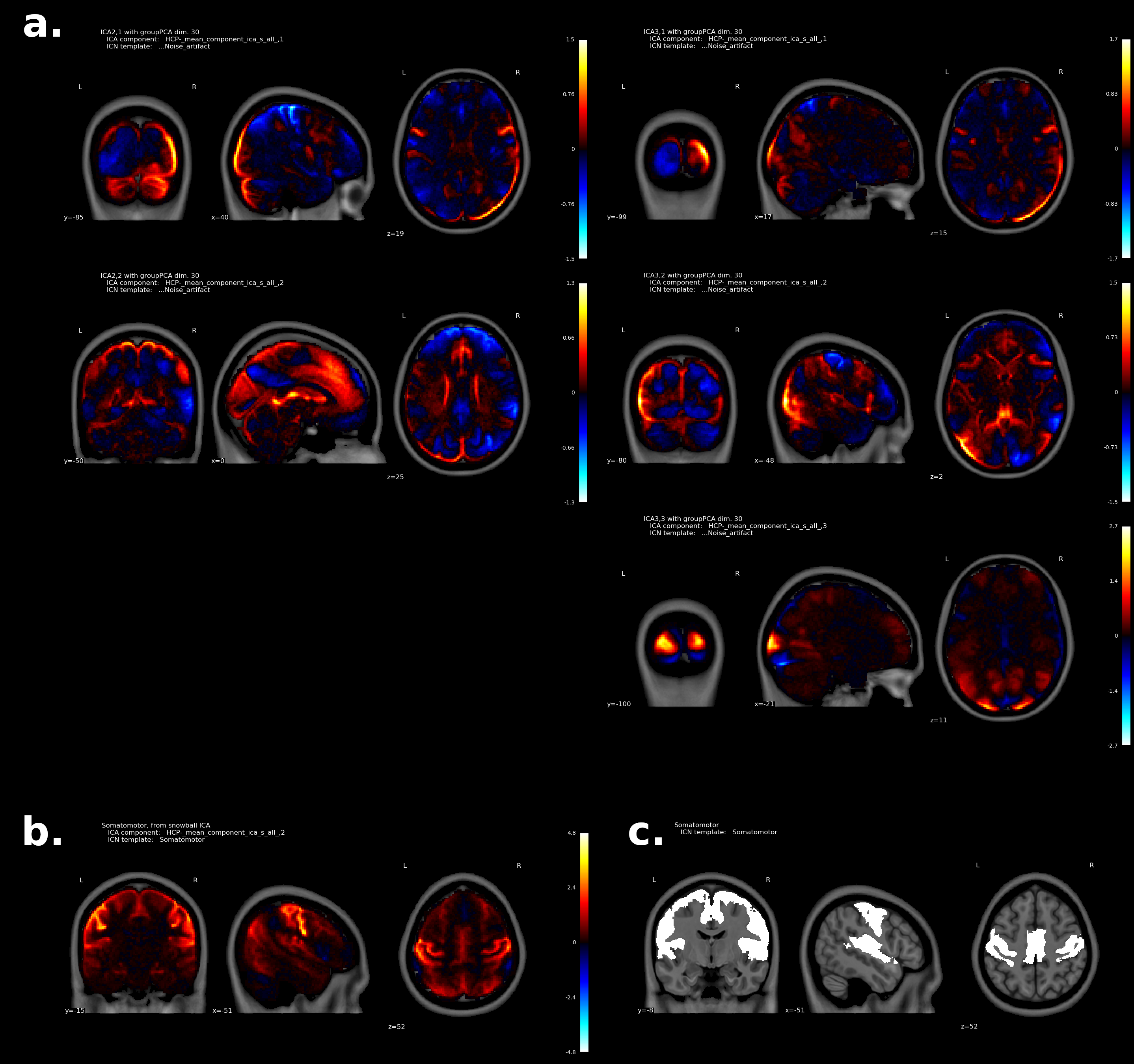

Supplement: Supplementary file 2 [file Image_2.TIFF]

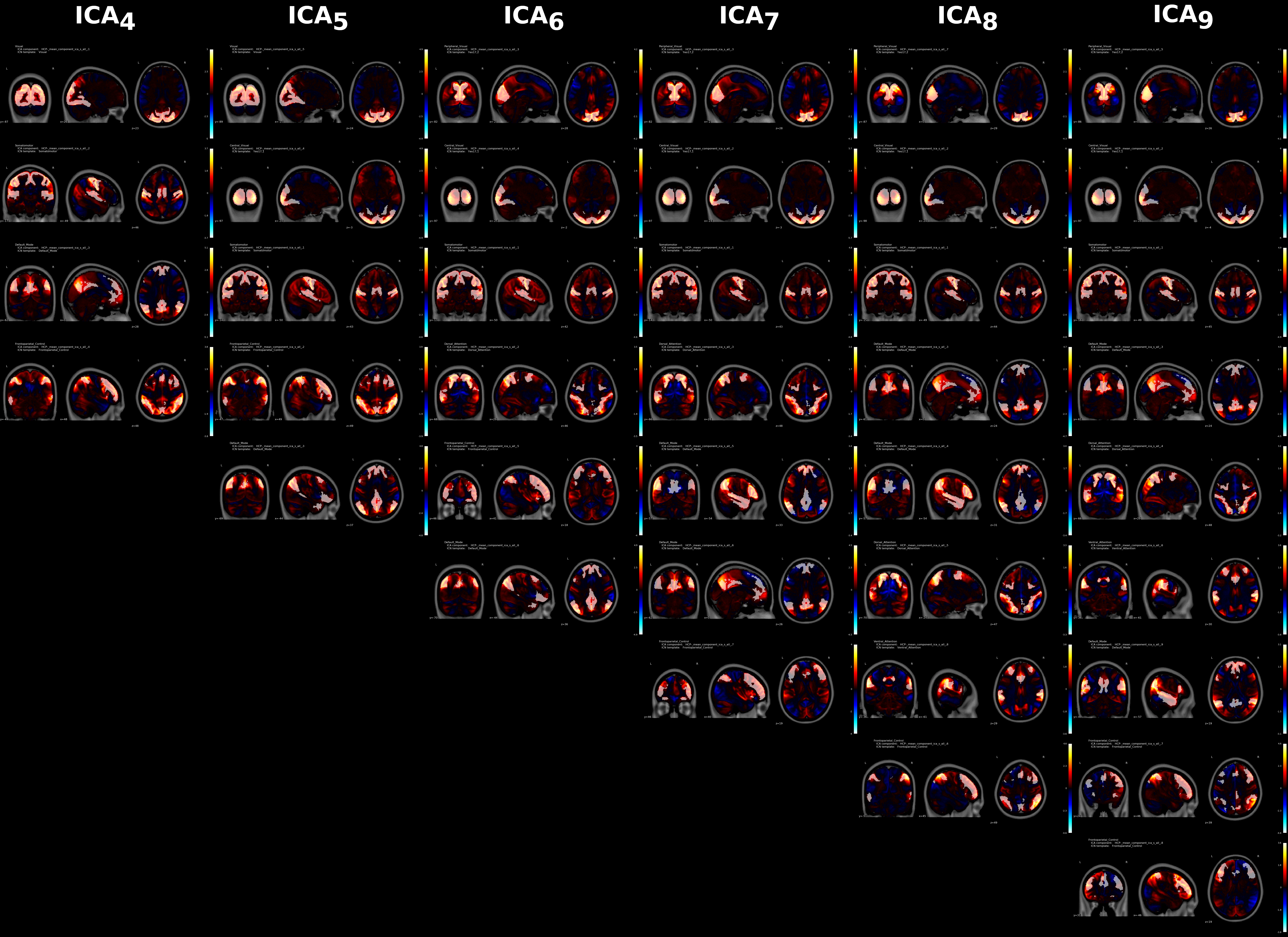

Supplement: Supplementary file 3 [file Image_3.TIFF]

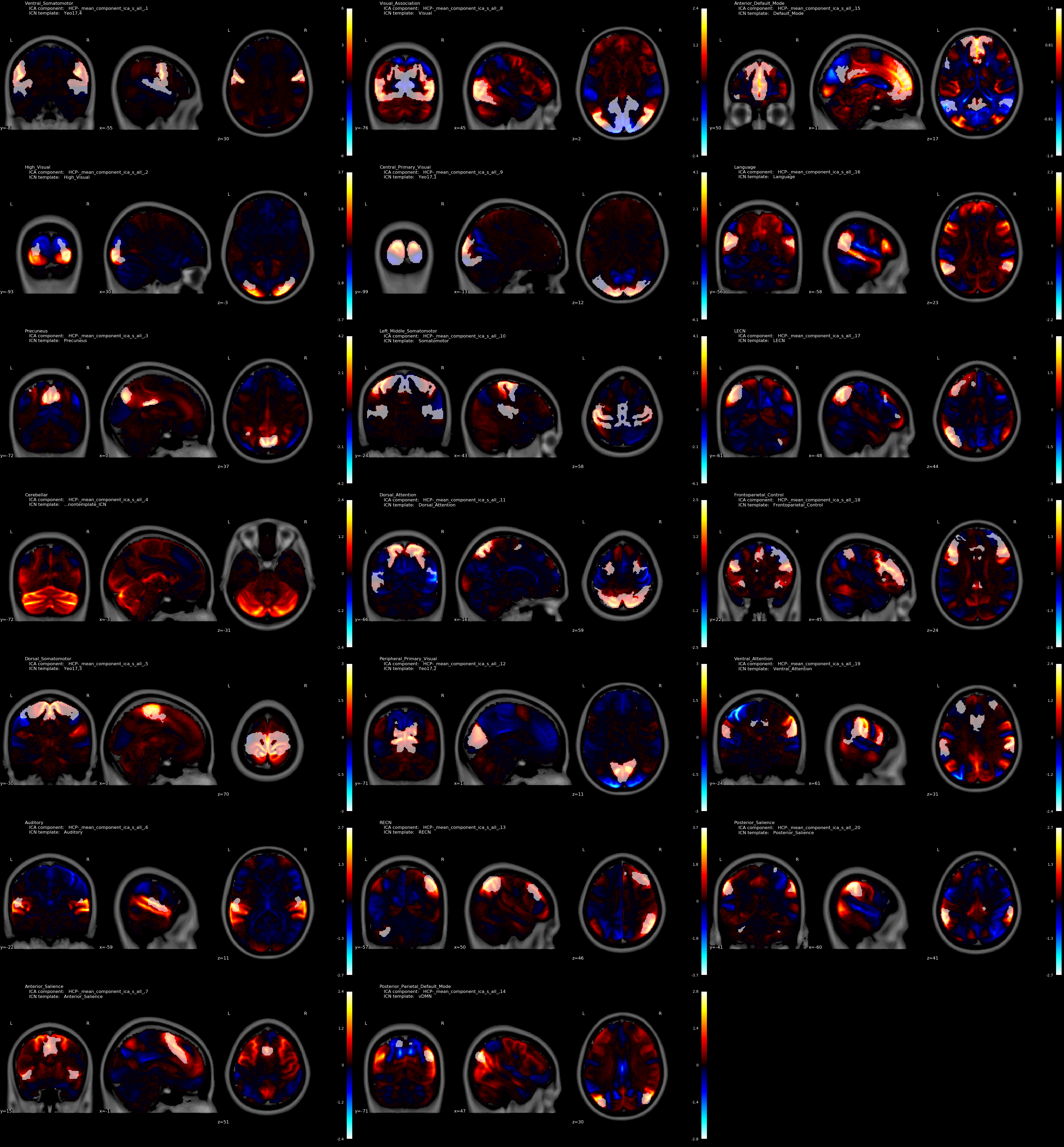

Supplement: Supplementary file 4 [file Image_4.TIFF]

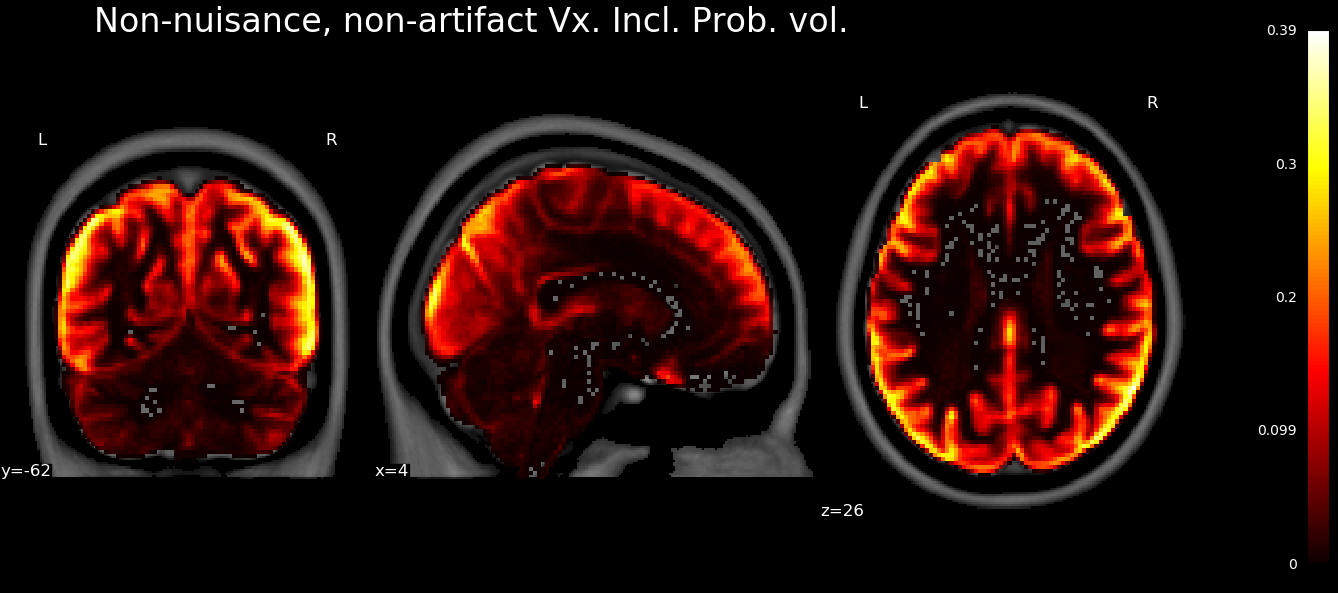

Supplement: Supplementary file 6 [file Image_6.TIFF]
