# Supplementary material for: Stable Meta-Networks, Noise, and Artifacts in the Human Connectome: Low- to High-Dimensional Independent Components Analysis as a Hierarchy of Intrinsic Connectivity Networks
Source: Front Neurosci. 2021 May 6;15:625737. doi: 10.3389/fnins.2021.625737 (PMC8134552; doi:10.3389/fnins.2021.625737)
Supplement: Supplementary file 5 [file Image_5.PDF]

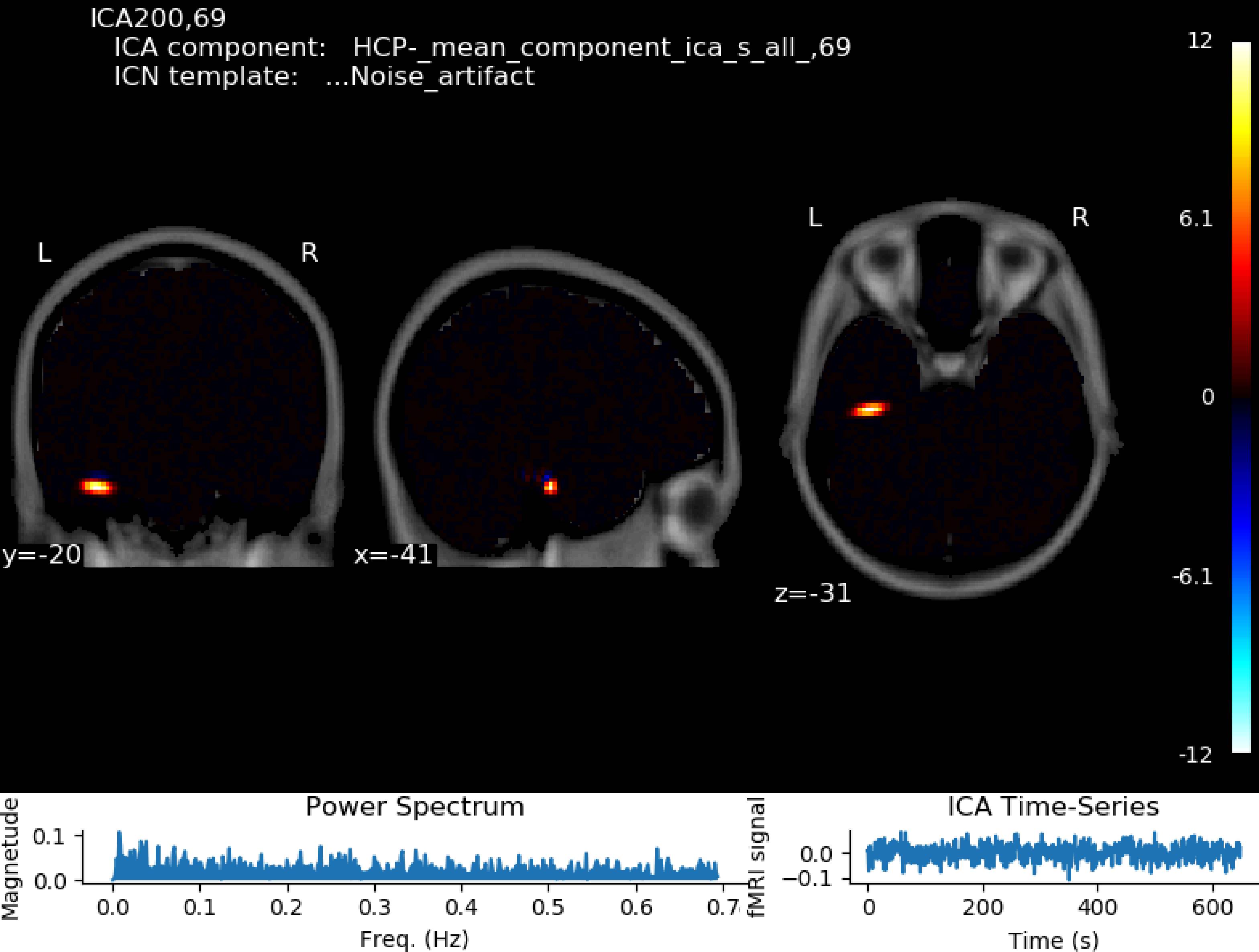
ICA200,69
ICA component: HCP-_mean_component_ica_s_all_,69
ICN template: ...Noise_artifact
L
R
L
R
y=-20
x=-41
z=-31
12
6.1
0
-6.1
-12
Power Spectrum
Magnetude
0.1
0.0
0.0
0.1
0.2
0.3
0.4
0.5
0.6
Freq. (Hz)
ICA Time-Series
fMRI signal
0.0
−0.1
0
200
400
600
Time (s)

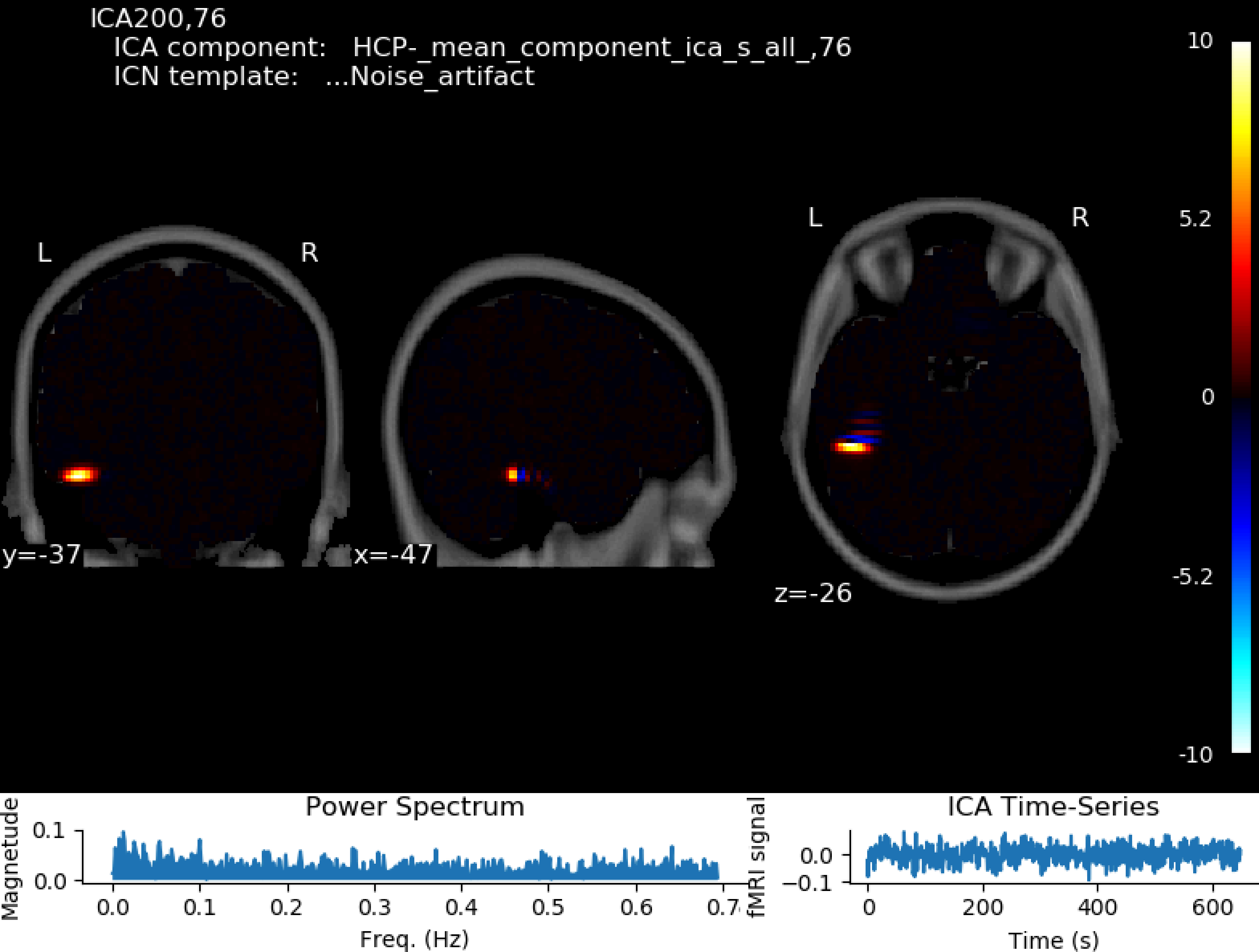
ICA200,76
ICA component: HCP-_mean_component_ica_s_all_,76
ICN template: ...Noise_artifact
L
R
L
R
y=-37
x=-47
z=-26
10
5.2
0
-5.2
-10
Power Spectrum
Magnetude
0.1
0.0
0.0
0.1
0.2
0.3
0.4
0.5
0.6
Freq. (Hz)
ICA Time-Series
fMRI signal
0.0
−0.1
0
200
400
600
Time (s)

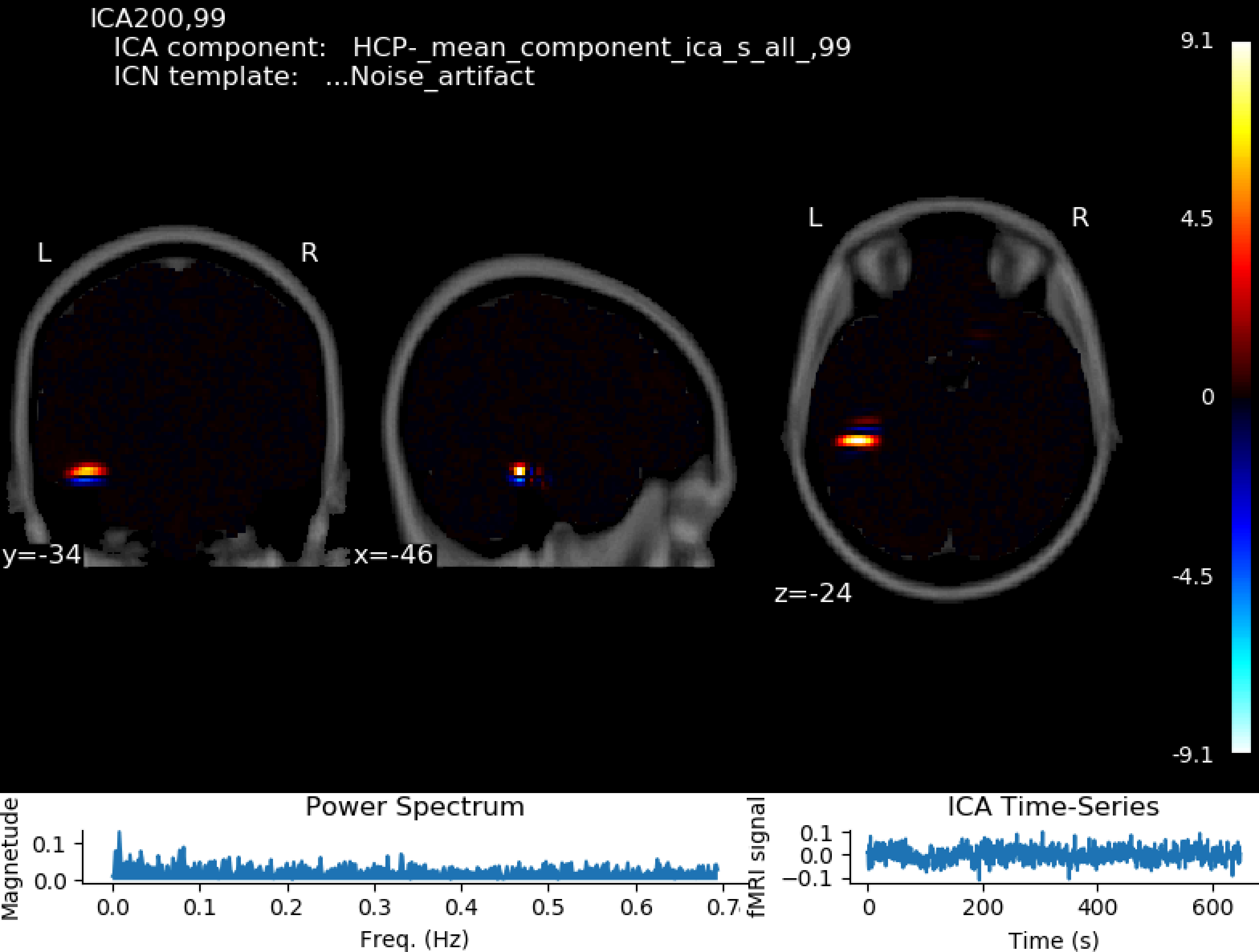

ICA200,99
ICA component: HCP-_mean_component_ica_s_all_,99
ICN template: ...Noise_artifact
L
R
L
R
y=-34
x=-46
z=-24
9.1
4.5
0
-4.5
-9.1
Power Spectrum
Magnetude
Freq. (Hz)
ICA Time-Series
fMRI signal
Time (s)

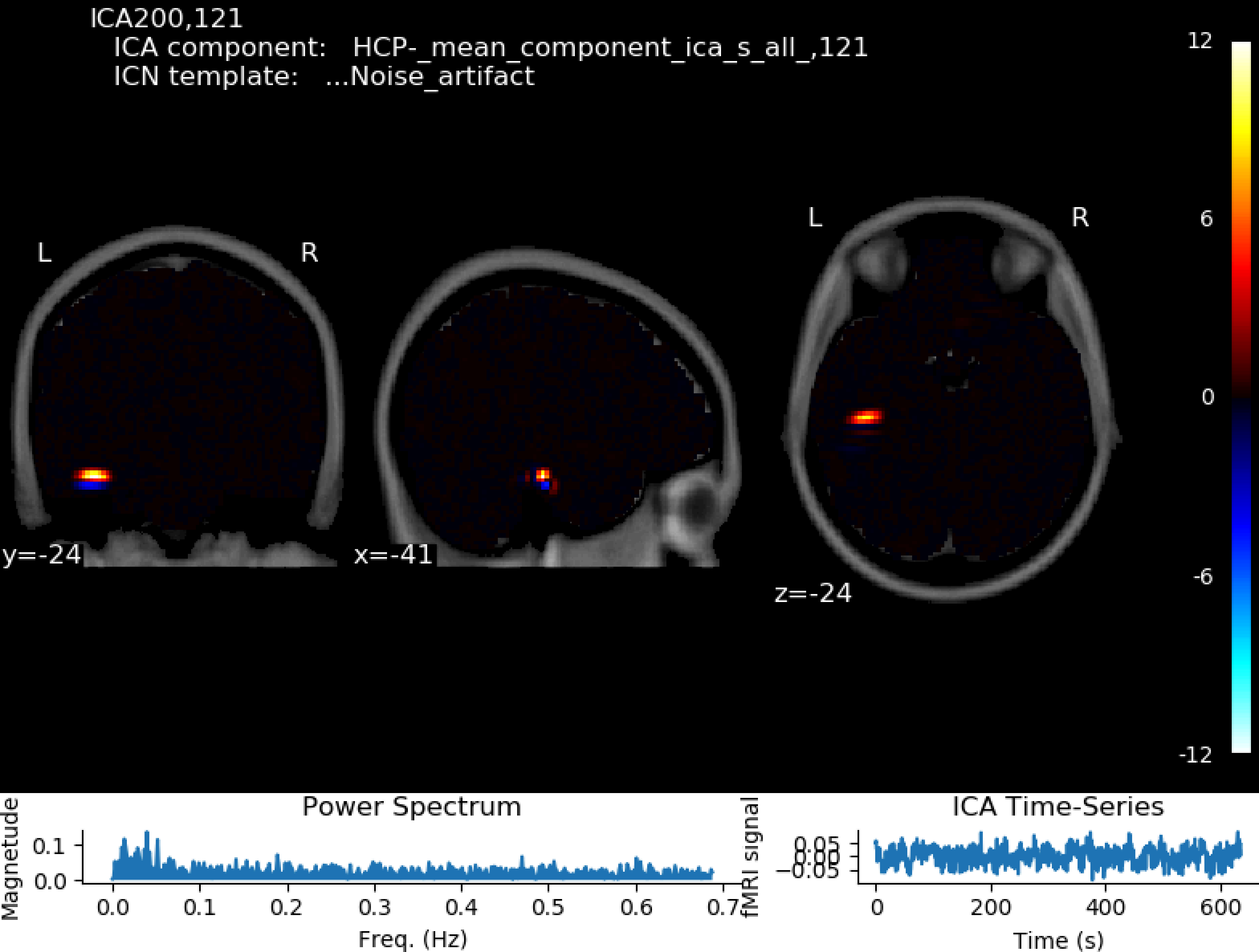
ICA200,121
ICA component: HCP-_mean_component_ica_s_all_,121
ICN template: ...Noise_artifact
L
R
y=-24
x=-41
z=-24
12
6
0
-6
-12
Power Spectrum
Magnetude
0.1
0.0
0.0
0.1
0.2
0.3
0.4
0.5
0.6
0.7
Freq. (Hz)
ICA Time-Series
fMRI signal
0.05
0.00
−0.05
0
200
400
600
Time (s)

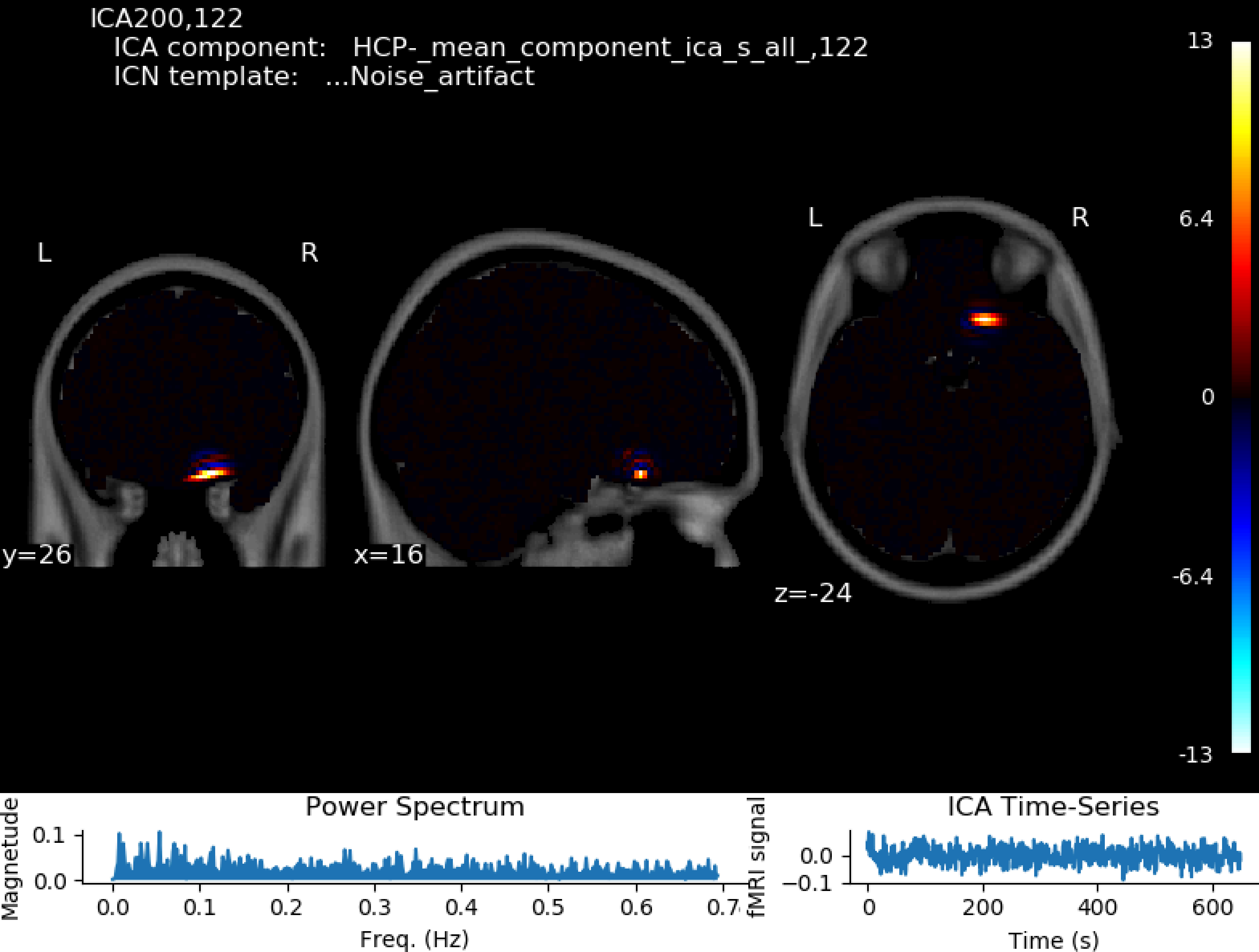
ICA200,122
ICA component: HCP-_mean_component_ica_s_all_,122
ICN template: ...Noise_artifact
L
R
y=26
x=16
L
R
z=-24
13
6.4
0
-6.4
-13
Power Spectrum
Magnetude
0.1
0.0
0.0
0.1
0.2
0.3
0.4
0.5
0.6
Freq. (Hz)
ICA Time-Series
fMRI signal
0.0
−0.1
0
200
400
600
Time (s)

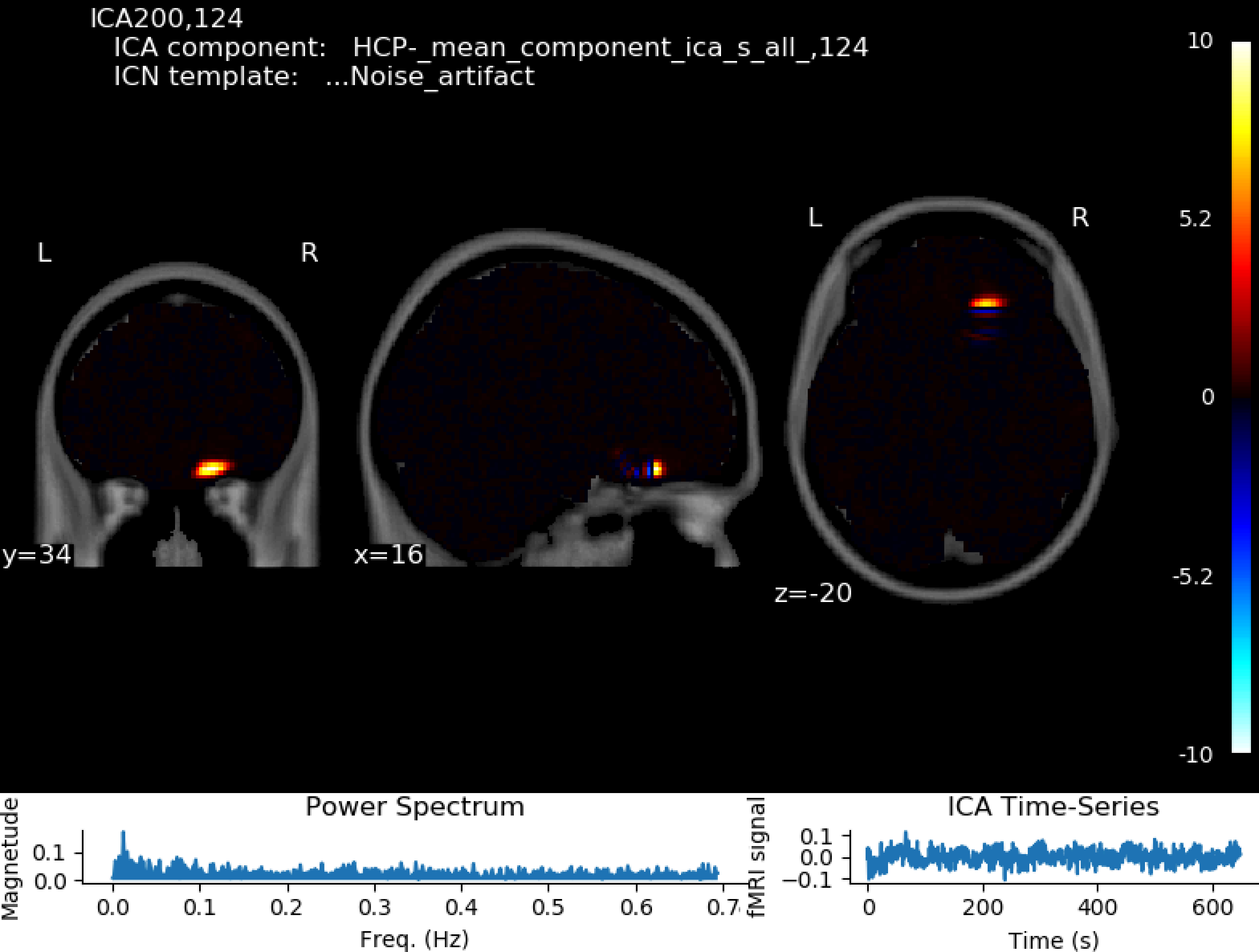
ICA200,124
ICA component: HCP-_mean_component_ica_s_all_,124
ICN template: ...Noise_artifact
L
R
L
R
y=34
x=16
z=-20
10
5.2
0
-5.2
-10
Power Spectrum
Magnetude
0.1
0.0
0.0
0.1
0.2
0.3
0.4
0.5
0.6
0.7
Freq. (Hz)
ICA Time-Series
fMRI signal
0.1
0.0
−0.1
0
200
400
600
Time (s)

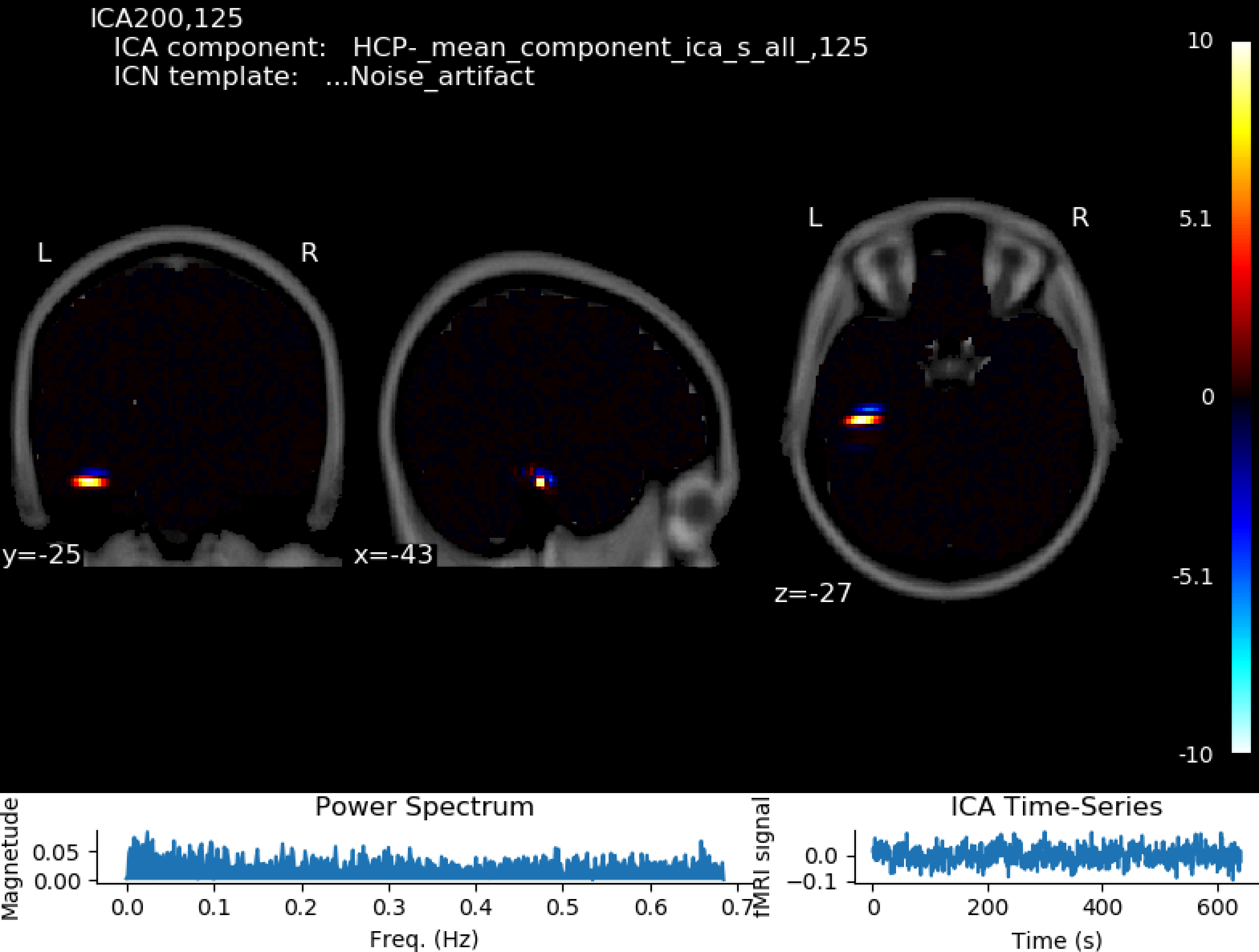
ICA200,125
ICA component: HCP-_mean_component_ica_s_all_,125
ICN template: ...Noise_artifact
L
R
y=-25
x=-43
L
R
z=-27
10
5.1
0
-5.1
-10
Power Spectrum
Magnetude
0.05
0.00
0.0
0.1
0.2
0.3
0.4
0.5
0.6
0.7
Freq. (Hz)
ICA Time-Series
fMRI signal
0.0
−0.1
0
200
400
600
Time (s)

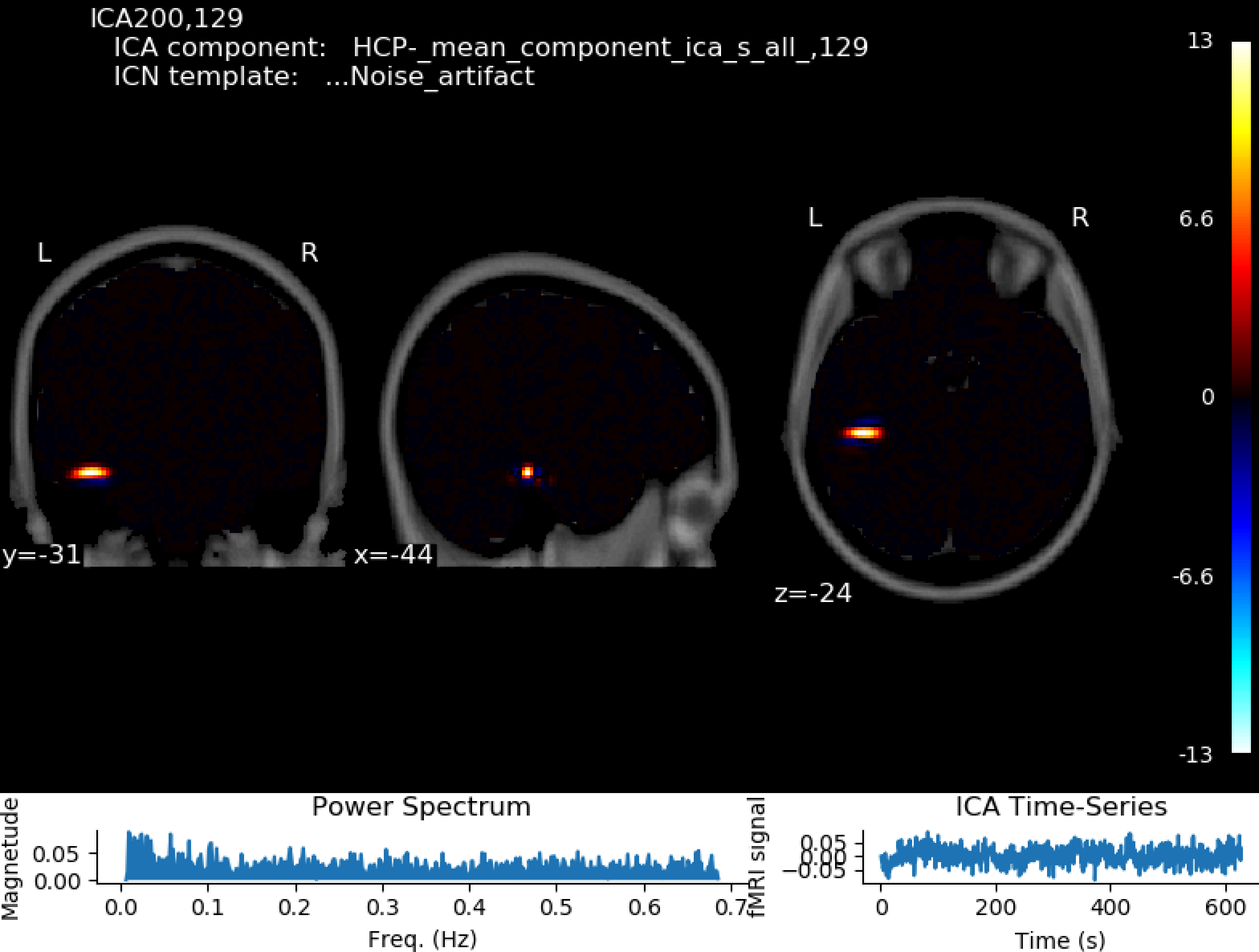
ICA200,129
ICA component: HCP-_mean_component_ica_s_all_,129
ICN template: ...Noise_artifact
L
R
y=-31
x=-44
L
R
z=-24
13
6.6
0
-6.6
-13
Power Spectrum
Magnetude
0.05
0.00
0.0
0.1
0.2
0.3
0.4
0.5
0.6
0.7
Freq. (Hz)
ICA Time-Series
fMRI signal
0.05
0.00
−0.05
0
200
400
600
Time (s)

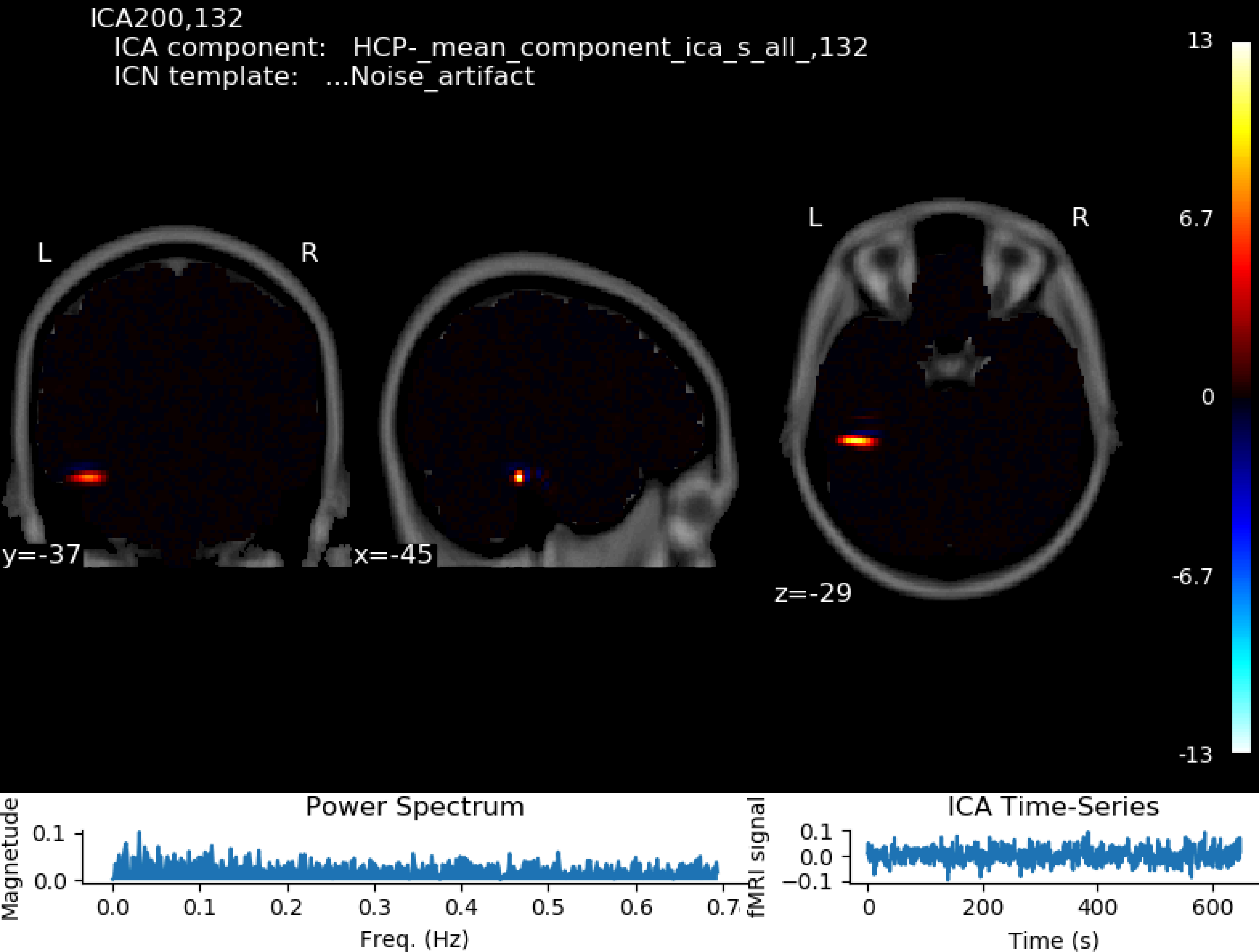
ICA200,132
ICA component: HCP-_mean_component_ica_s_all_,132
ICN template: ...Noise_artifact
L
R
y=-37
x=-45
L
R
z=-29
13
6.7
0
-6.7
-13
Power Spectrum
Magnetude
0.1
0.0
0.0
0.1
0.2
0.3
0.4
0.5
0.6
0.7
Freq. (Hz)
ICA Time-Series
fMRI signal
0.1
0.0
−0.1
0
200
400
600
Time (s)

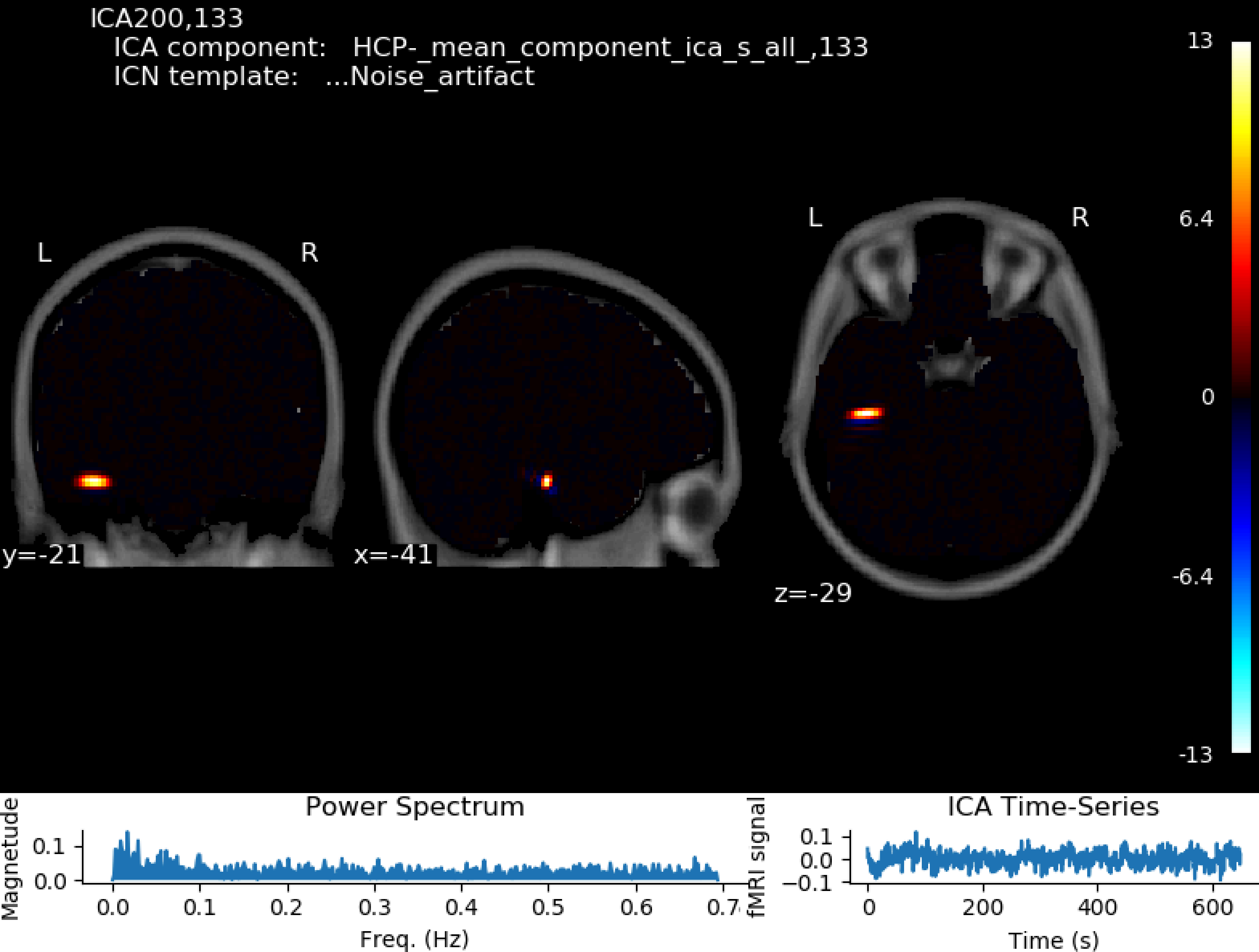

ICA200,133
ICA component: HCP-_mean_component_ica_s_all_,133
ICN template: ...Noise_artifact
L
R
L
R
y=-21
x=-41
z=-29
13
6.4
0
-6.4
-13
Power Spectrum
Magnetude
0.1
0.0
0.0
0.1
0.2
0.3
0.4
0.5
0.6
Freq. (Hz)
ICA Time-Series
fMRI signal
0.1
0.0
−0.1
0
200
400
600
Time (s)

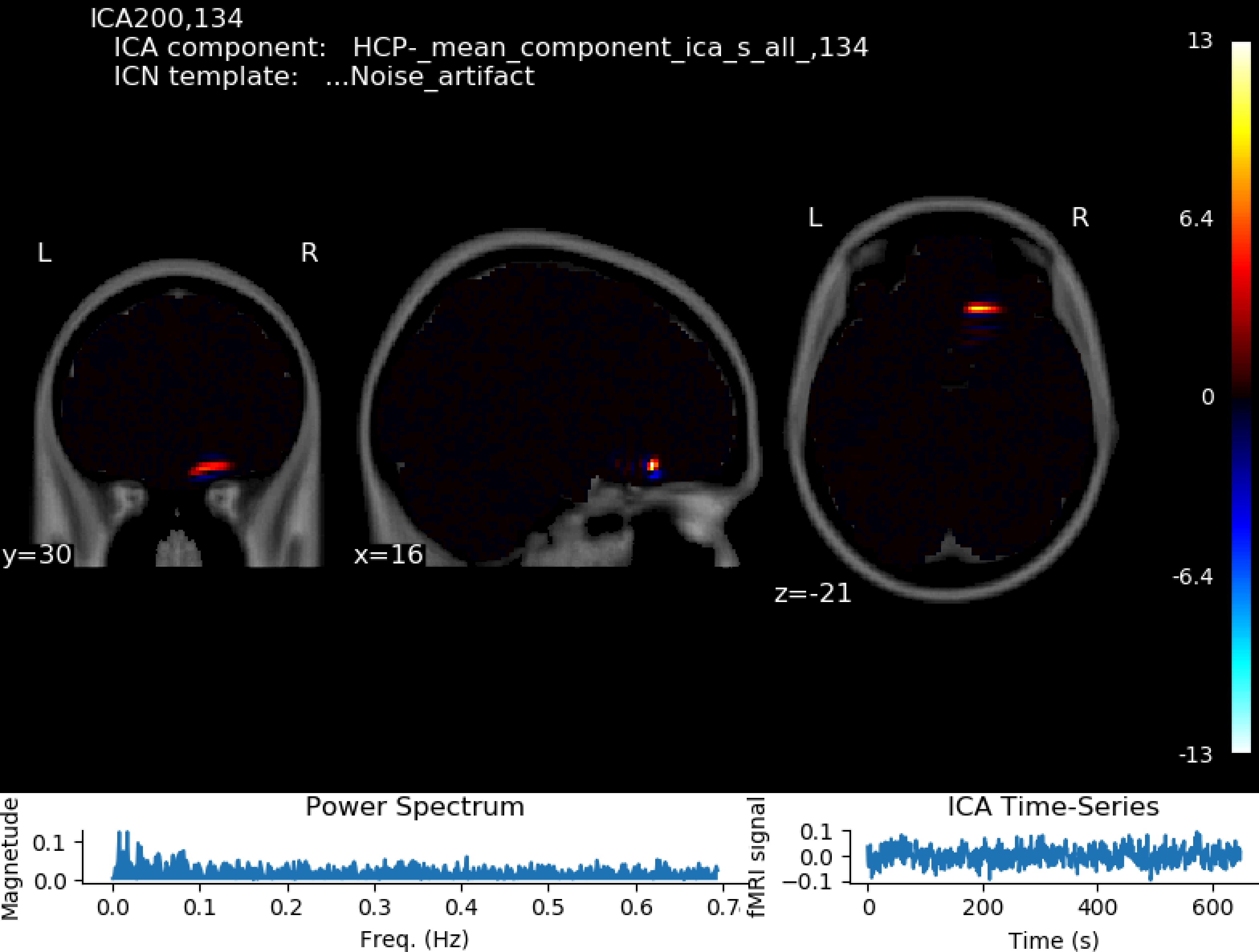

ICA200,134
ICA component: HCP-_mean_component_ica_s_all_,134
ICN template: ...Noise_artifact
L
R
y=30
x=16
z=-21
13
6.4
0
-6.4
-13
Power Spectrum
Magnetude
0.1
0.0
0.0
0.1
0.2
0.3
0.4
0.5
0.6
0.7
Freq. (Hz)
ICA Time-Series
fMRI signal
0.1
0.0
−0.1
0
200
400
600
Time (s)

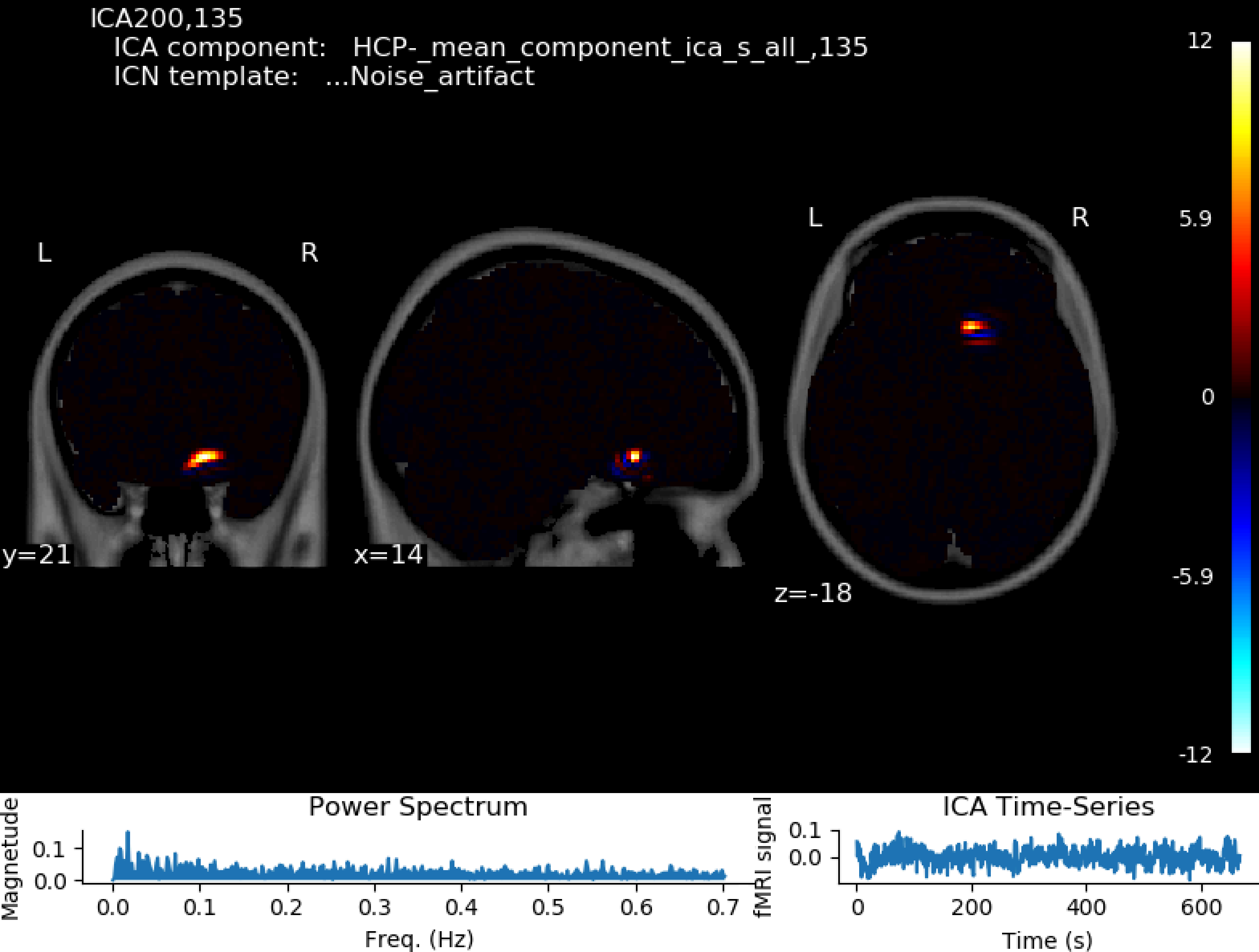

ICA200,135
ICA component: HCP-_mean_component_ica_s_all_,135
ICN template: ...Noise_artifact
L
R
y=21
x=14
L
R
z=-18
12
5.9
0
-5.9
-12
Power Spectrum
Magnetude
0.1
0.0
0.0
0.1
0.2
0.3
0.4
0.5
0.6
0.7
Freq. (Hz)
ICA Time-Series
fMRI signal
0.1
0.0
0
200
400
600
Time (s)

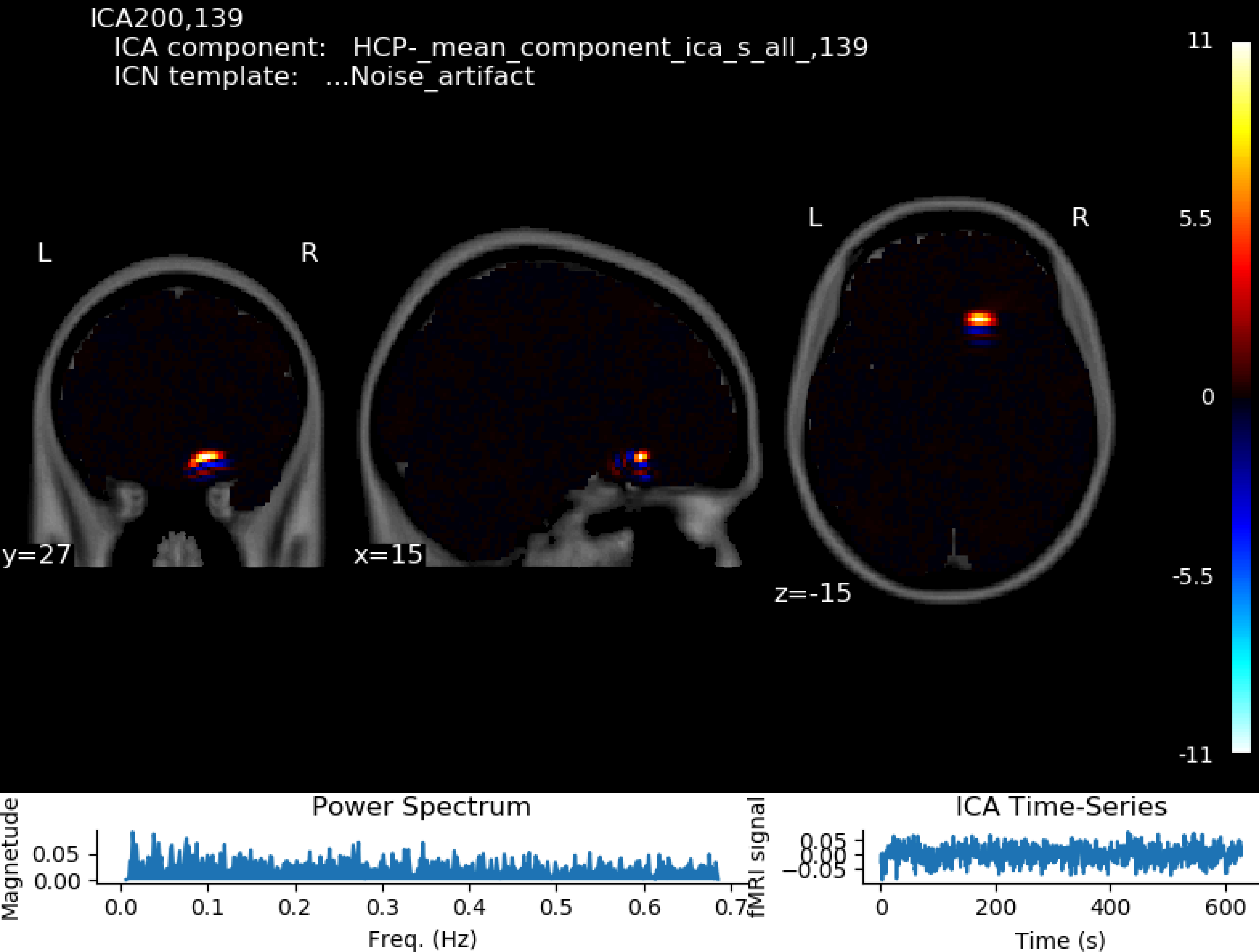
ICA200,139
ICA component: HCP-_mean_component_ica_s_all_,139
ICN template: ...Noise_artifact
L
R
y=27
x=15
z=-15
11
5.5
0
-5.5
-11
Power Spectrum
Magnetude
Freq. (Hz)
ICA Time-Series
fMRI signal
Time (s)

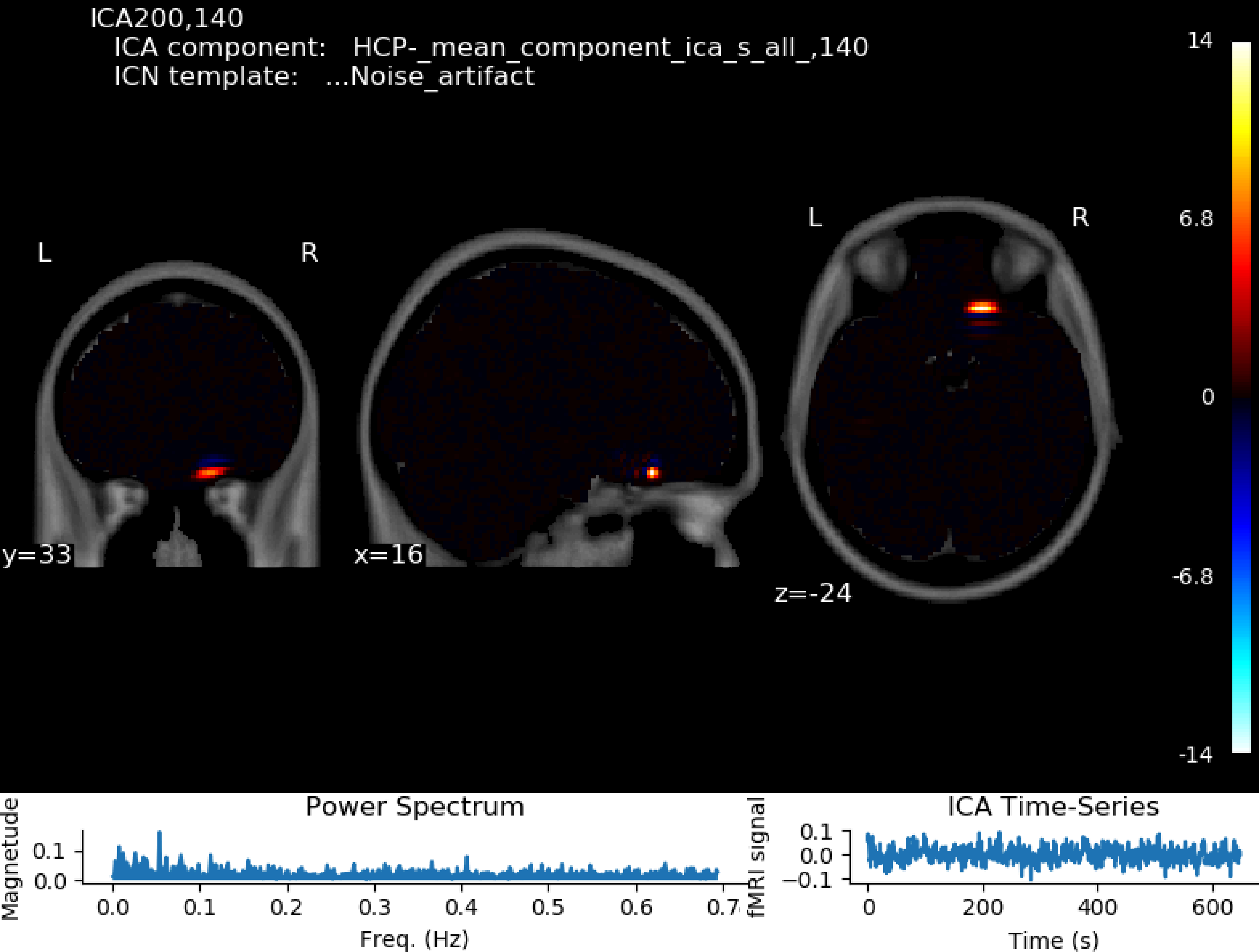
ICA200,140
ICA component: HCP-_mean_component_ica_s_all_,140
ICN template: ...Noise_artifact
L
R
y=33
x=16
L
R
z=-24
14
6.8
0
-6.8
-14
Power Spectrum
Magnetude
0.1
0.0
0.0
0.1
0.2
0.3
0.4
0.5
0.6
0.7
Freq. (Hz)
ICA Time-Series
fMRI signal
0.1
0.0
−0.1
0
200
400
600
Time (s)

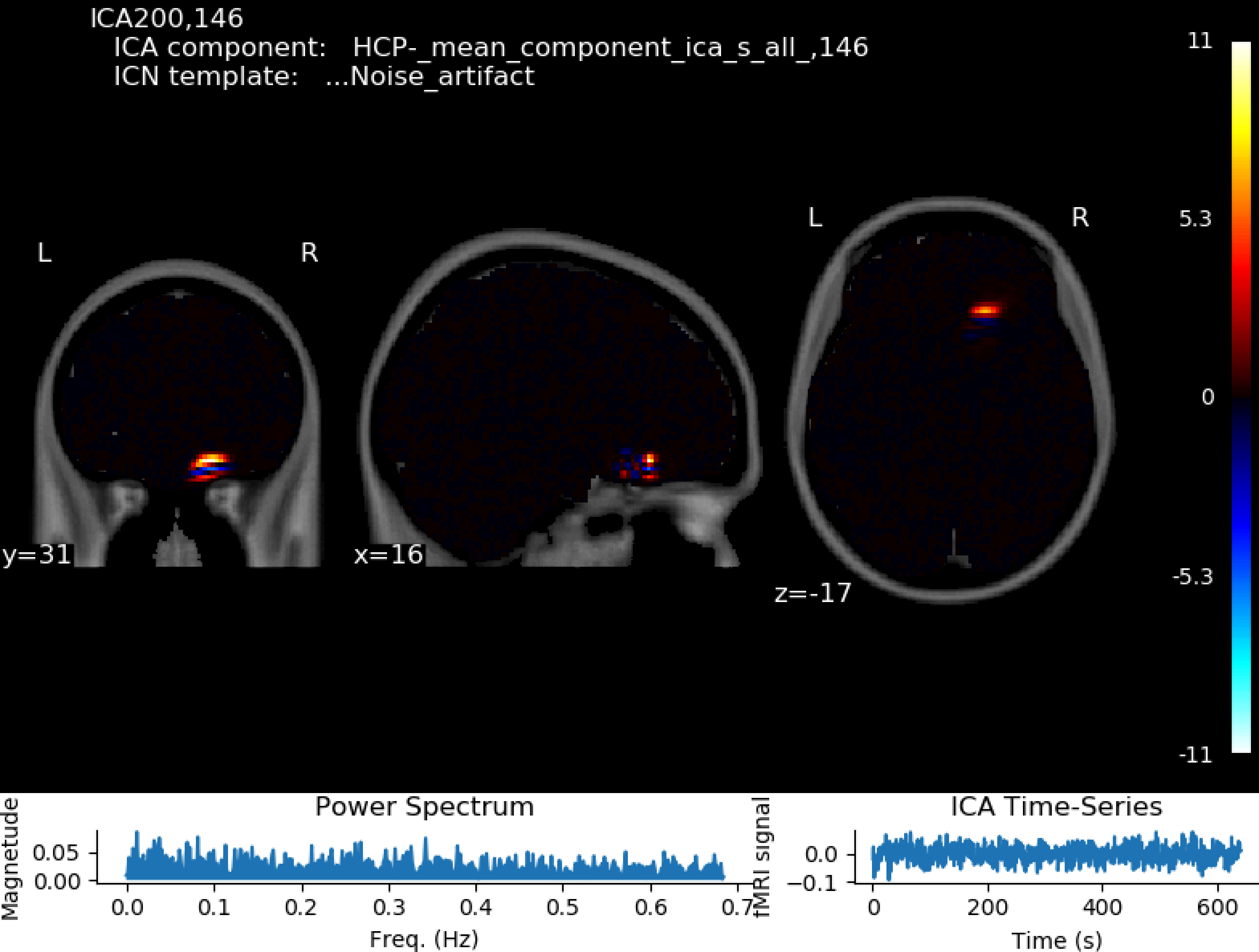
ICA200,146
ICA component: HCP-_mean_component_ica_s_all_,146
ICN template: ...Noise_artifact
L
R
L
R
y=31
x=16
z=-17
11
5.3
0
-5.3
-11
Power Spectrum
Magnetude
0.05
0.00
0.0
0.1
0.2
0.3
0.4
0.5
0.6
0.7
Freq. (Hz)
ICA Time-Series
fMRI signal
0.0
−0.1
0
200
400
600
Time (s)

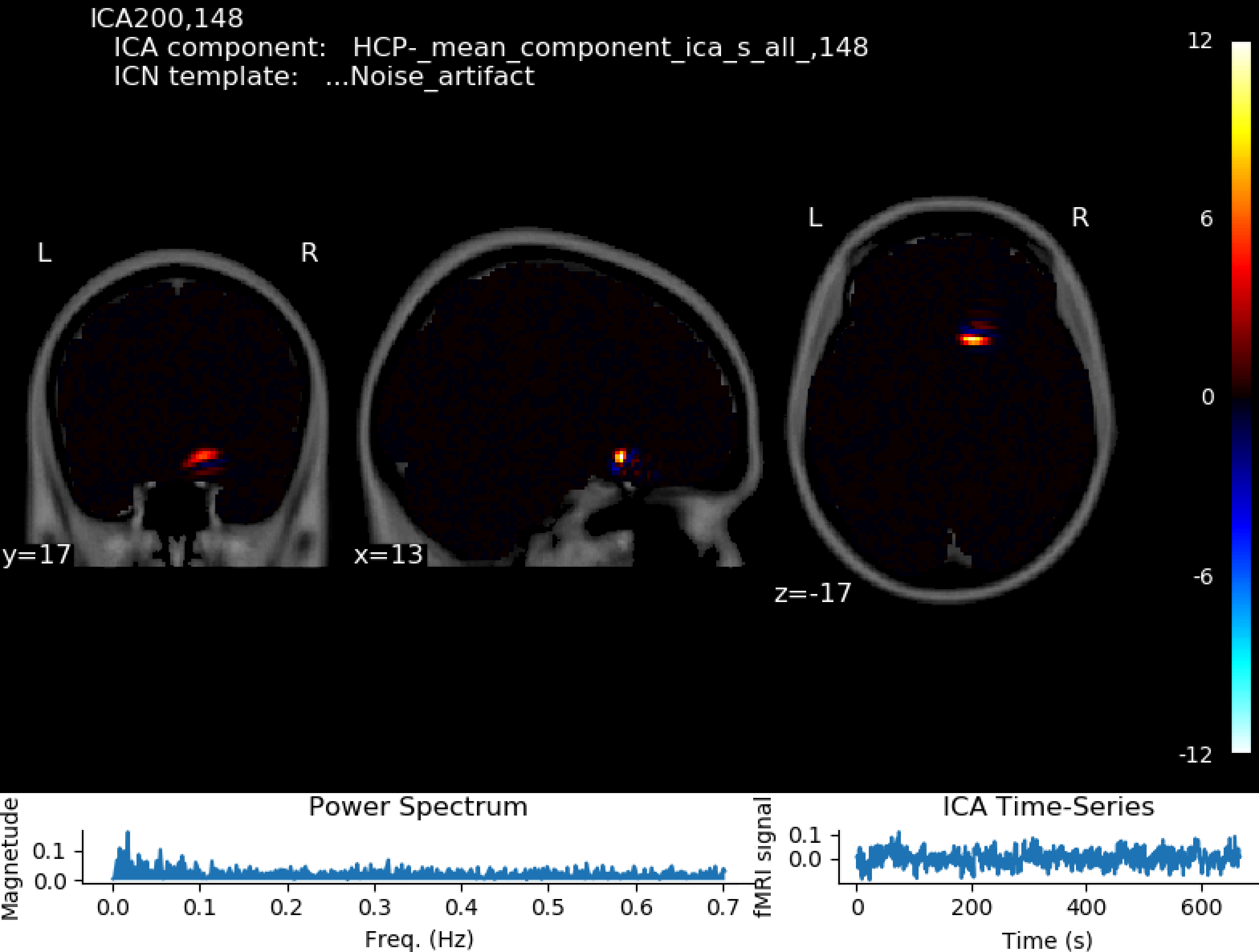
ICA200,148
ICA component: HCP-_mean_component_ica_s_all_,148
ICN template: ...Noise_artifact
L
R
y=17
x=13
z=-17
12
6
0
-6
-12
Power Spectrum
Magnetude
Freq. (Hz)
0.0
0.1
0.2
0.3
0.4
0.5
0.6
0.7
ICA Time-Series
fMRI signal
Time (s)
0
200
400
600

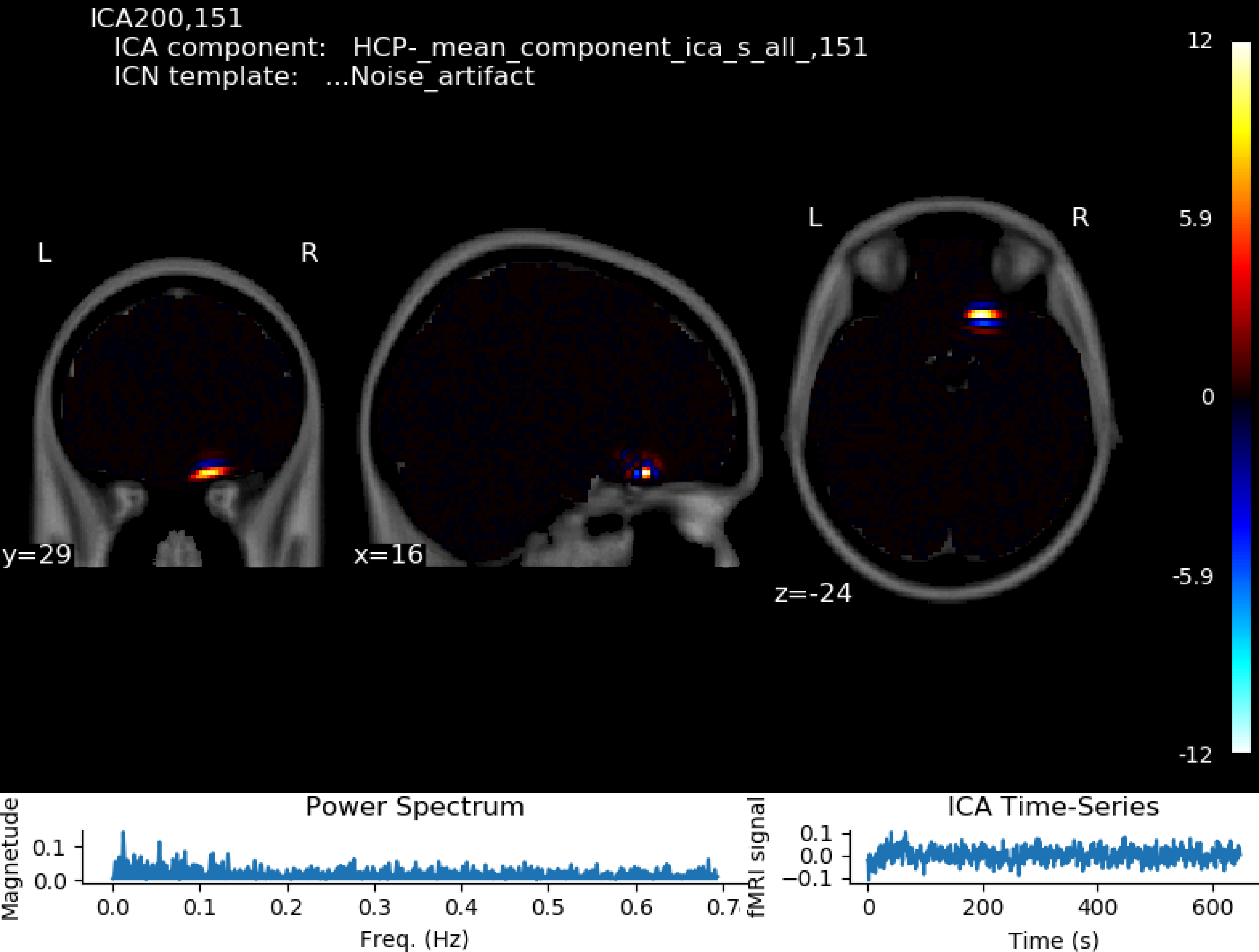
ICA200,151
ICA component: HCP-_mean_component_ica_s_all_,151
ICN template: ...Noise_artifact
L
R
y=29
x=16
z=-24
12
5.9
0
-5.9
-12
Power Spectrum
Magnetude
Freq. (Hz)
ICA Time-Series
fMRI signal
Time (s)

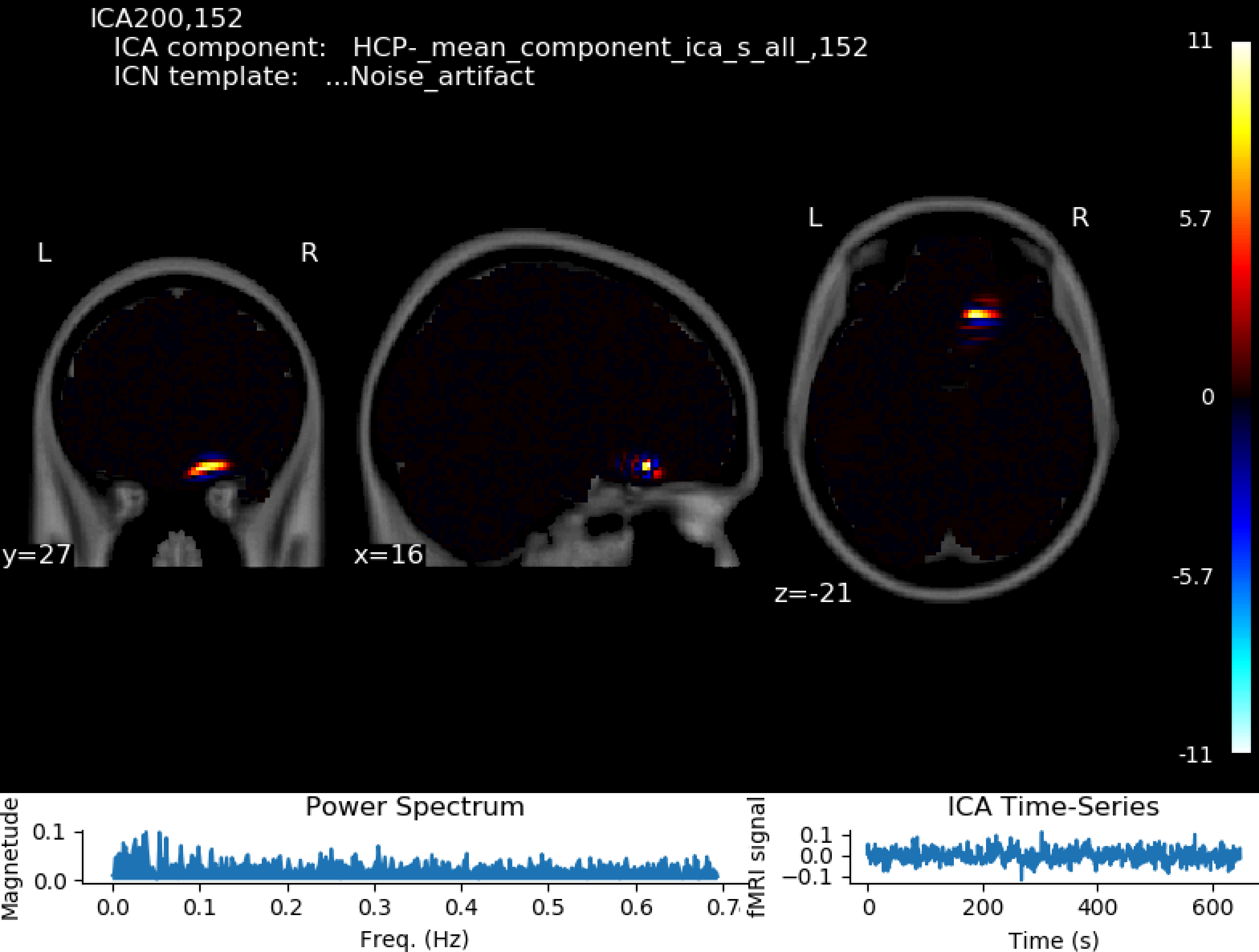
ICA200,152
ICA component: HCP-_mean_component_ica_s_all_,152
ICN template: ...Noise_artifact
L
R
y=27
x=16
L
R
z=-21
11
5.7
0
-5.7
-11
Power Spectrum
Magnetude
Freq. (Hz)
ICA Time-Series
fMRI signal
Time (s)

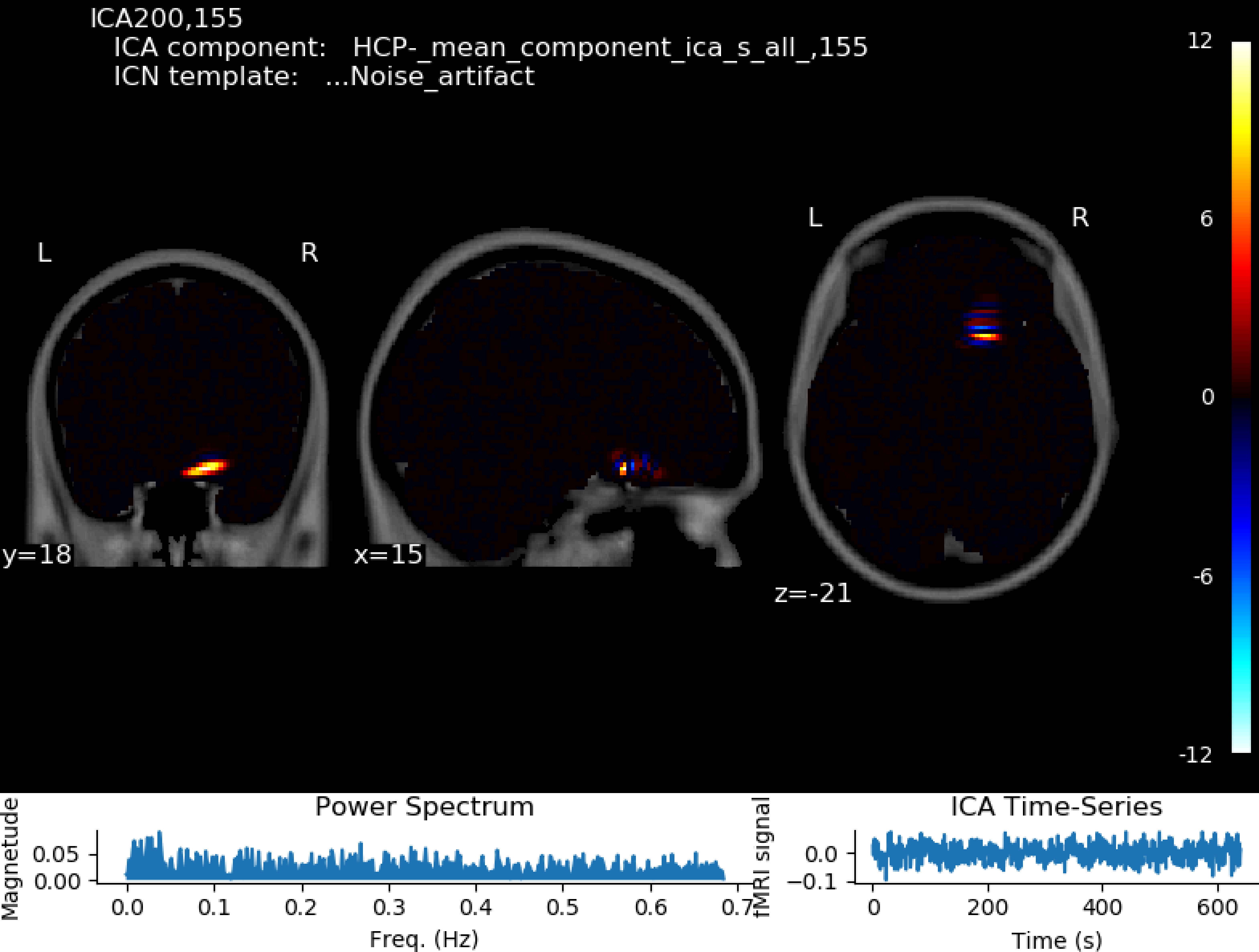
ICA200,155
ICA component: HCP-_mean_component_ica_s_all_,155
ICN template: ...Noise_artifact
L
R
y=18
x=15
z=-21
12
6
0
-6
-12
Power Spectrum
Magnetude
0.05
0.00
0.0
0.1
0.2
0.3
0.4
0.5
0.6
0.7
Freq. (Hz)
ICA Time-Series
fMRI signal
0.0
−0.1
0
200
400
600
Time (s)

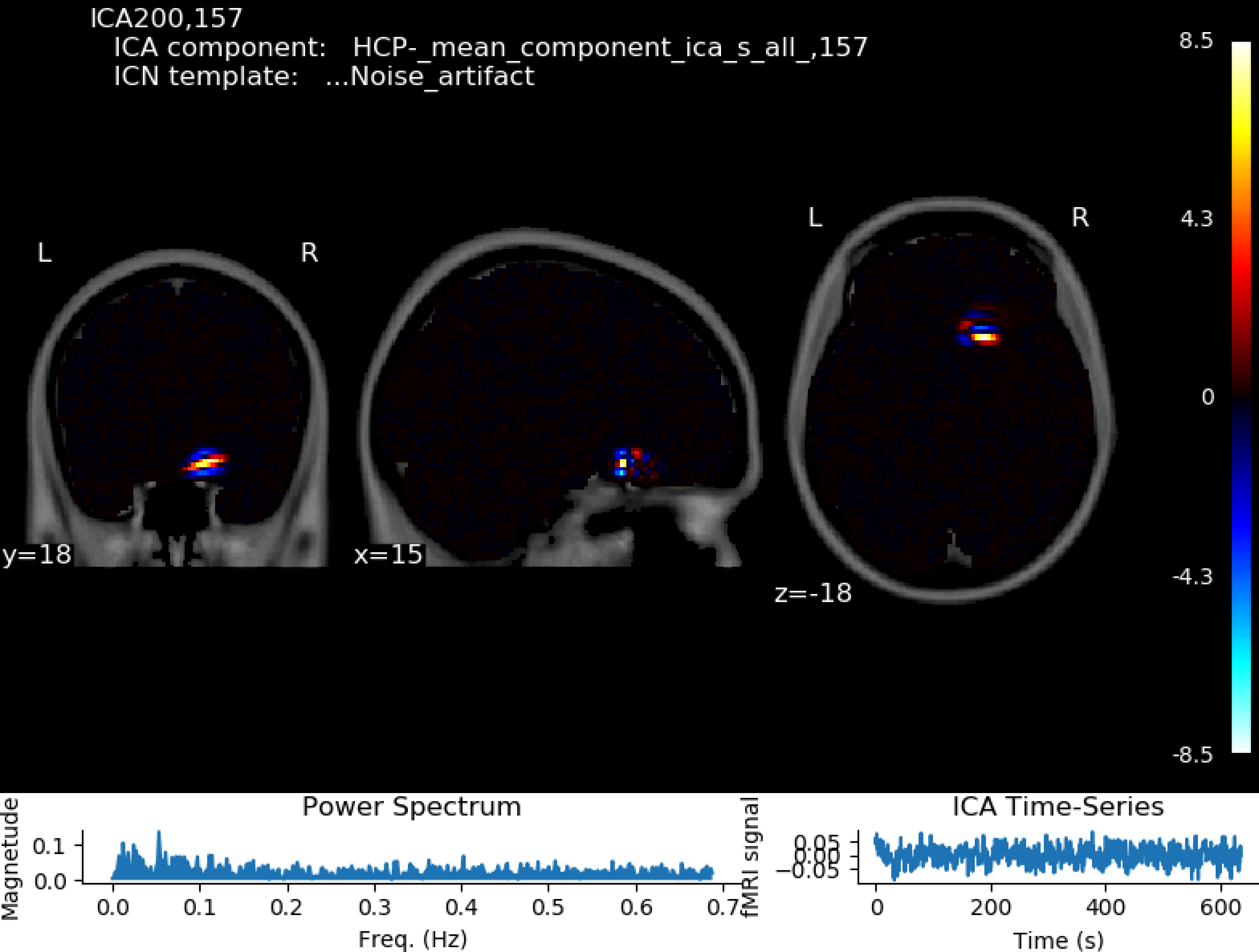

ICA200,157
ICA component: HCP-_mean_component_ica_s_all_,157
ICN template: ...Noise_artifact
L
R
y=18
x=15
z=-18
8.5
4.3
0
-4.3
-8.5
Power Spectrum
Magnetude
0.1
0.0
0.0
0.1
0.2
0.3
0.4
0.5
0.6
0.7
Freq. (Hz)
ICA Time-Series
fMRI signal
0.05
0.00
−0.05
0
200
400
600
Time (s)

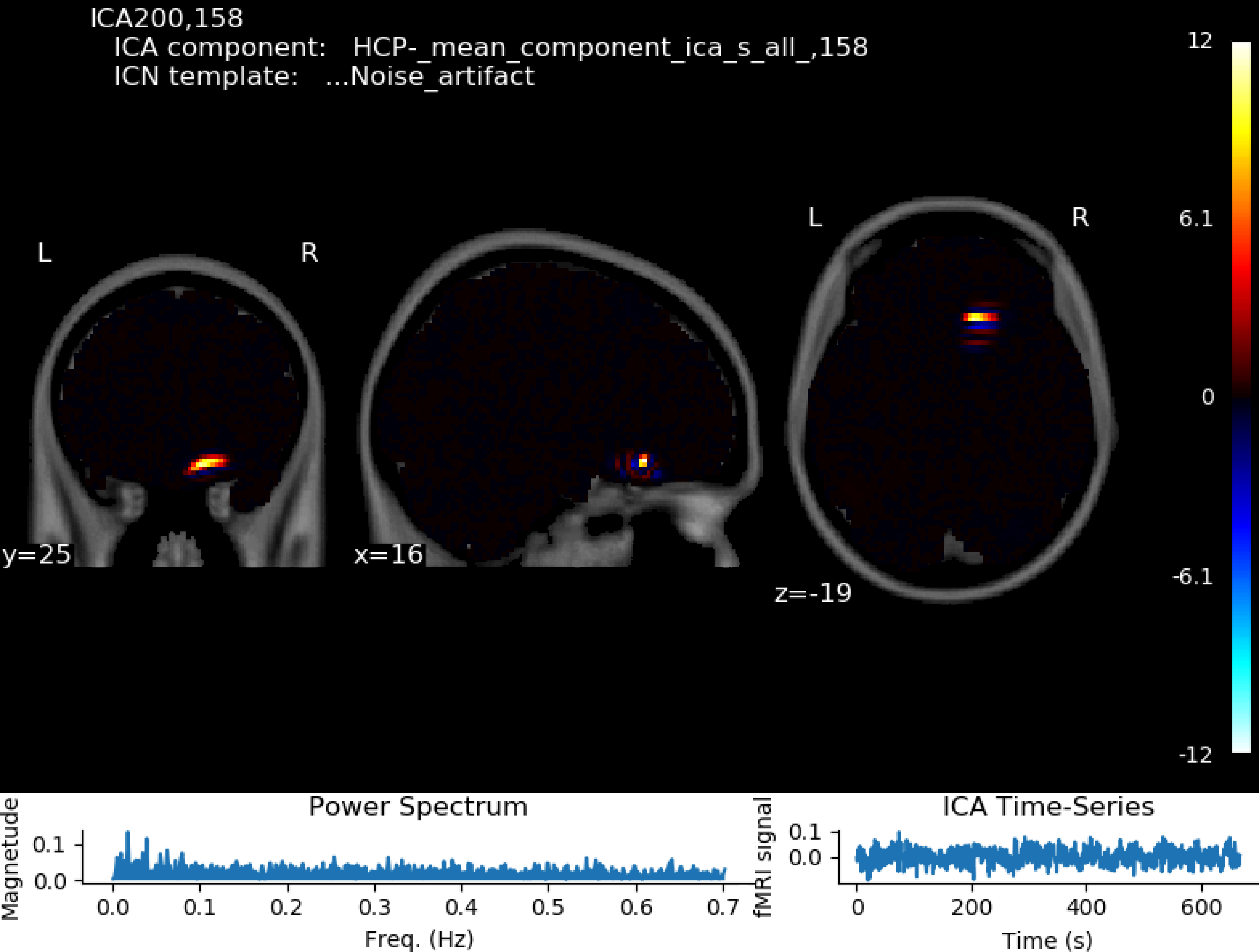

ICA200,158
ICA component: HCP-_mean_component_ica_s_all_,158
ICN template: ...Noise_artifact
L
R
L
R
y=25
x=16
z=-19
12
6.1
0
-6.1
-12
Power Spectrum
Magnetude
0.1
0.0
0.0
0.1
0.2
0.3
0.4
0.5
0.6
0.7
Freq. (Hz)
ICA Time-Series
fMRI signal
0.1
0.0
0
200
400
600
Time (s)

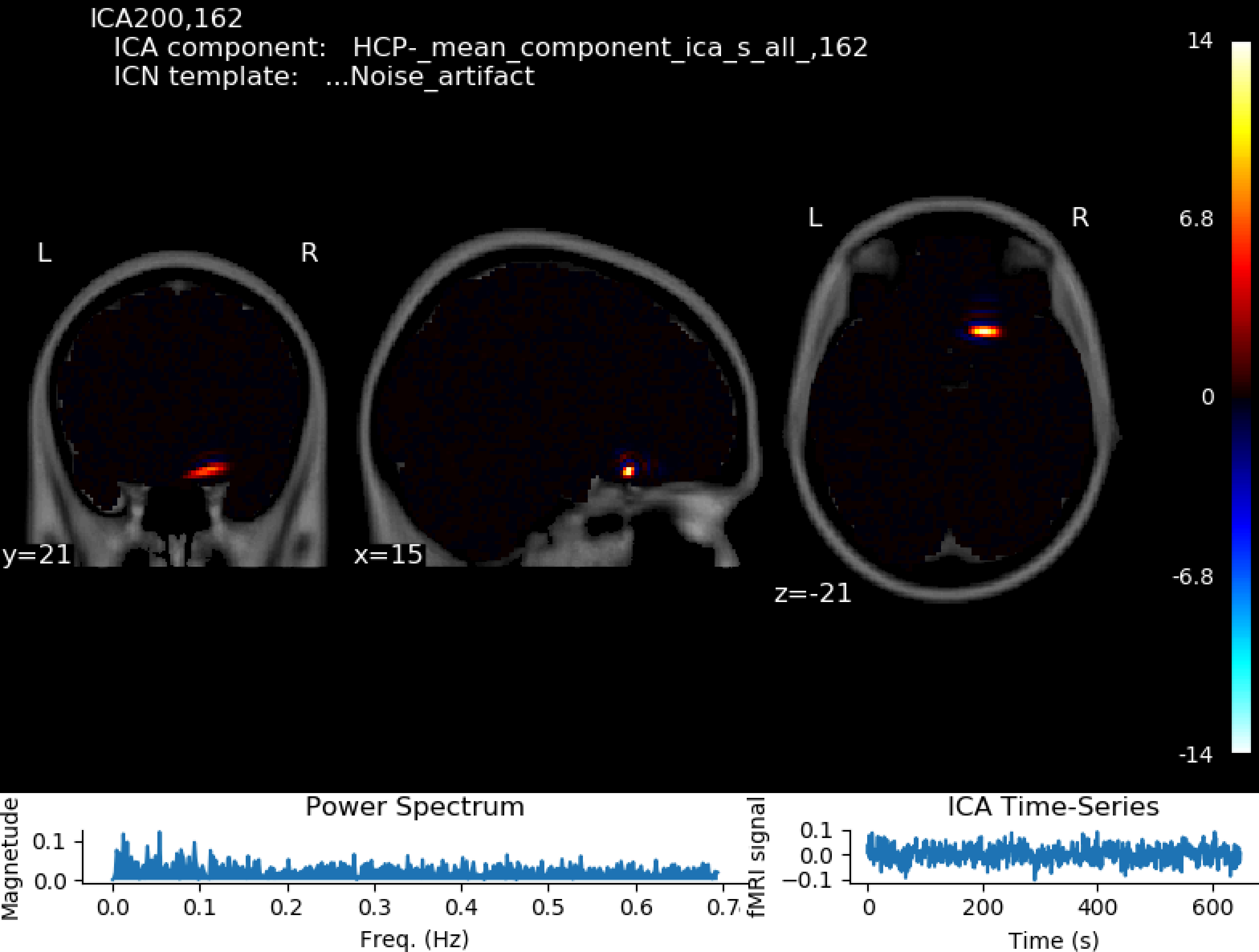
ICA200,162
ICA component: HCP-_mean_component_ica_s_all_,162
ICN template: ...Noise_artifact
L
R
y=21
x=15
z=-21
14
6.8
0
-6.8
-14
Power Spectrum
Magnetude
Freq. (Hz)
ICA Time-Series
fMRI signal
Time (s)

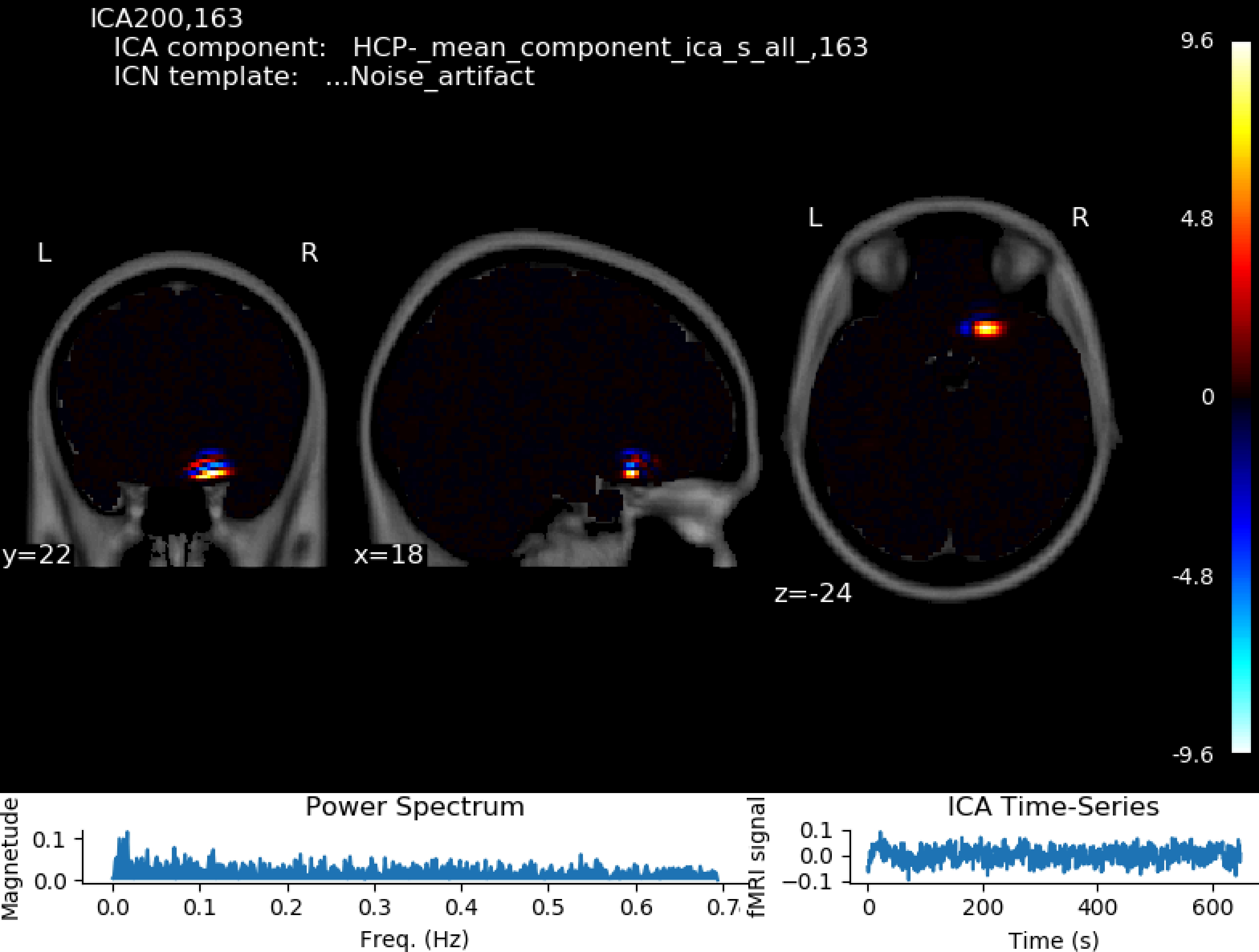

ICA200,163
ICA component: HCP-_mean_component_ica_s_all_,163
ICN template: ...Noise_artifact
L
R
L
R
y=22
x=18
z=-24
9.6
4.8
0
-4.8
-9.6
Power Spectrum
Magnetude
0.1
0.0
0.0
0.1
0.2
0.3
0.4
0.5
0.6
0.7
Freq. (Hz)
ICA Time-Series
fMRI signal
0.1
0.0
−0.1
0
200
400
600
Time (s)

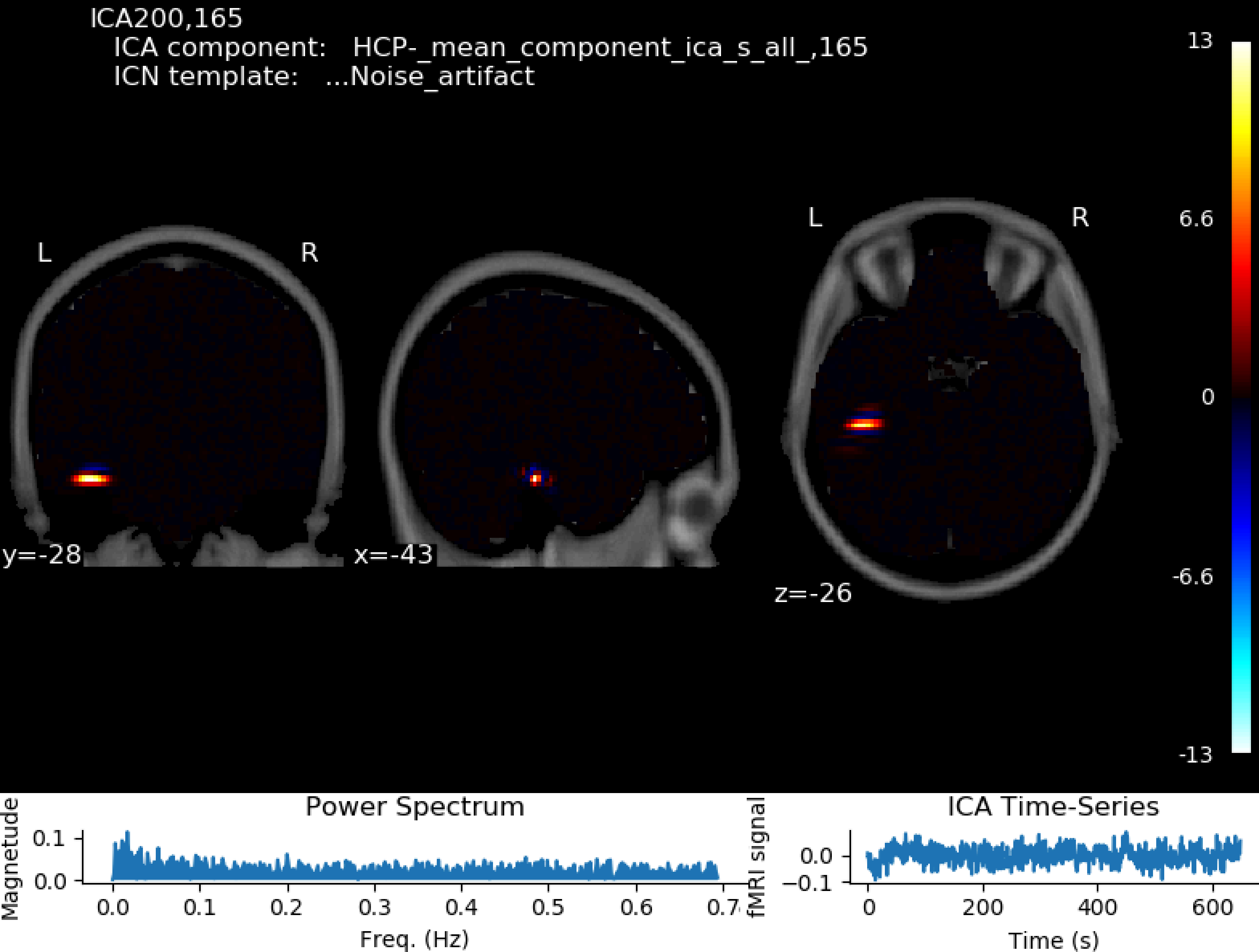
ICA200,165
ICA component: HCP-_mean_component_ica_s_all_,165
ICN template: ...Noise_artifact
L
R
L
R
y=-28
x=-43
z=-26
13
6.6
0
-6.6
-13
Power Spectrum
Magnetude
Freq. (Hz)
ICA Time-Series
fMRI signal
Time (s)

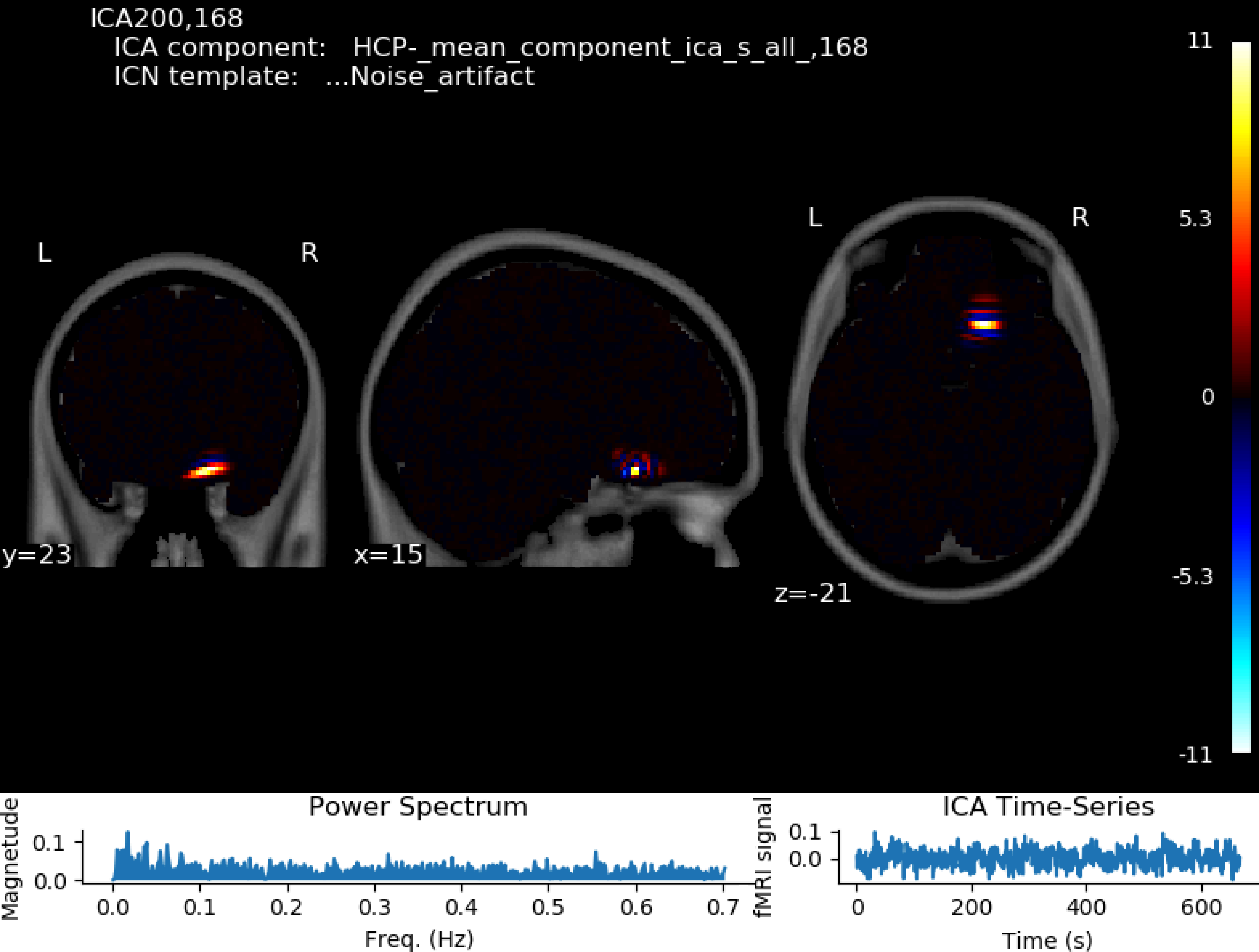
ICA200,168
ICA component: HCP-_mean_component_ica_s_all_,168
ICN template: ...Noise_artifact
L
R
y=23
x=15
L
R
z=-21
11
5.3
0
-5.3
-11
Power Spectrum
Magnetude
0.1
0.0
0.0
0.1
0.2
0.3
0.4
0.5
0.6
0.7
Freq. (Hz)
ICA Time-Series
fMRI signal
0.1
0.0
0
200
400
600
Time (s)

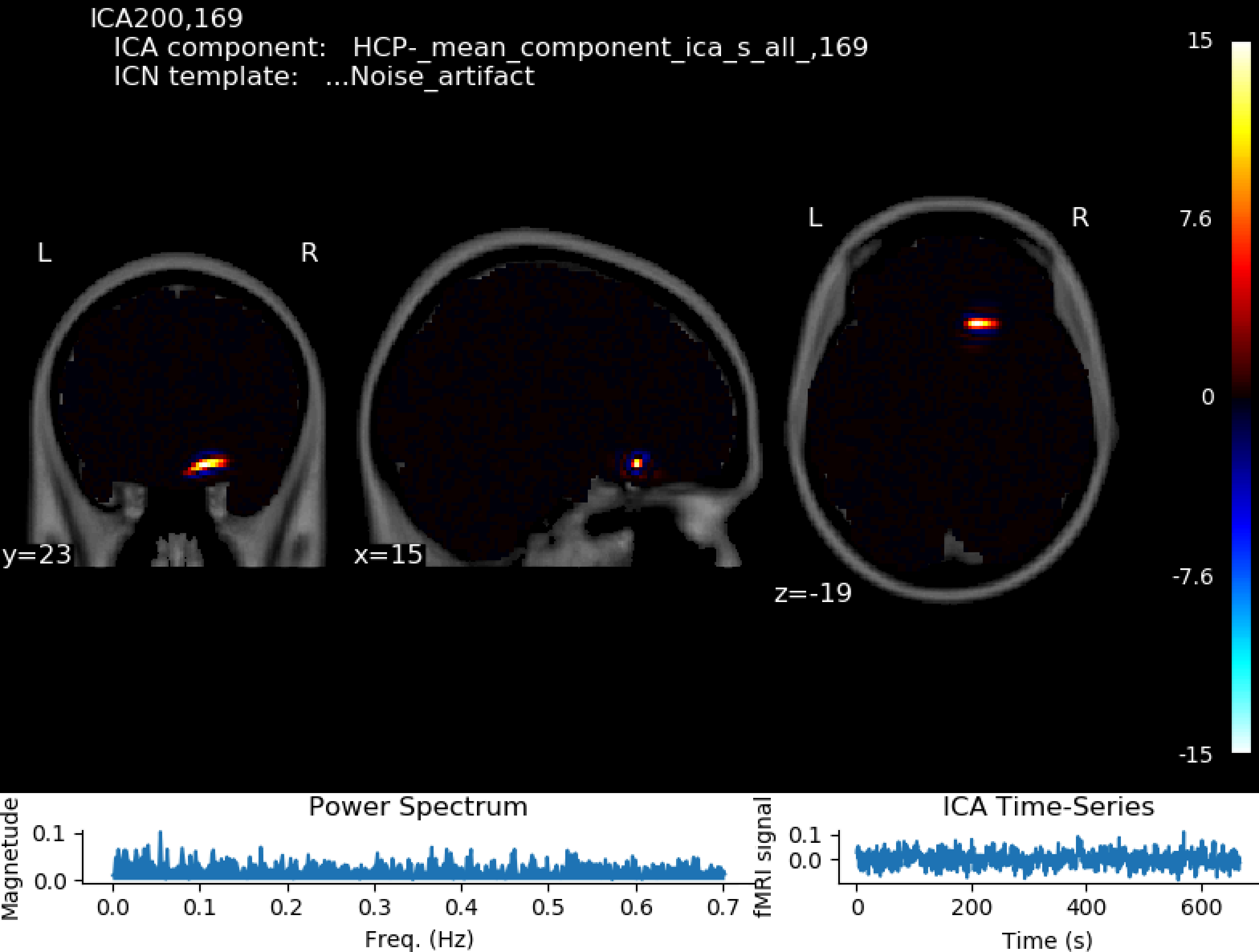

ICA200,169
ICA component: HCP-_mean_component_ica_s_all_,169
ICN template: ...Noise_artifact
L
R
L
R
y=23
x=15
z=-19
15
7.6
0
-7.6
-15
Power Spectrum
Magnetude
0.1
0.0
0.0
0.1
0.2
0.3
0.4
0.5
0.6
0.7
Freq. (Hz)
ICA Time-Series
fMRI signal
0.1
0.0
0
200
400
600
Time (s)

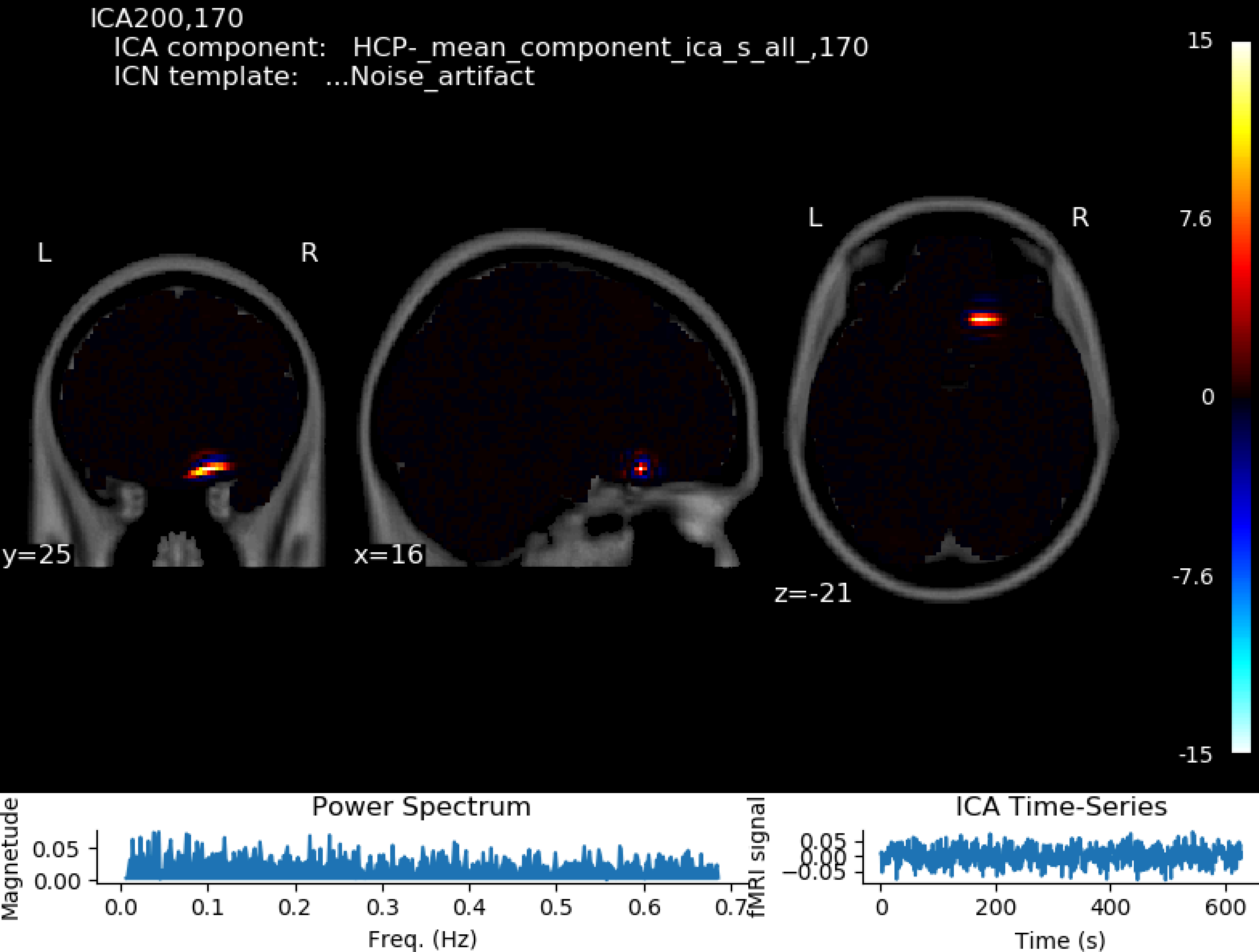
ICA200,170
ICA component: HCP-_mean_component_ica_s_all_,170
ICN template: ...Noise_artifact
L
R
y=25
x=16
L
R
z=-21
15
7.6
0
-7.6
-15
Power Spectrum
Magnetude
0.05
0.00
0.0
0.1
0.2
0.3
0.4
0.5
0.6
0.7
Freq. (Hz)
ICA Time-Series
fMRI signal
0.05
0.00
−0.05
0
200
400
600
Time (s)

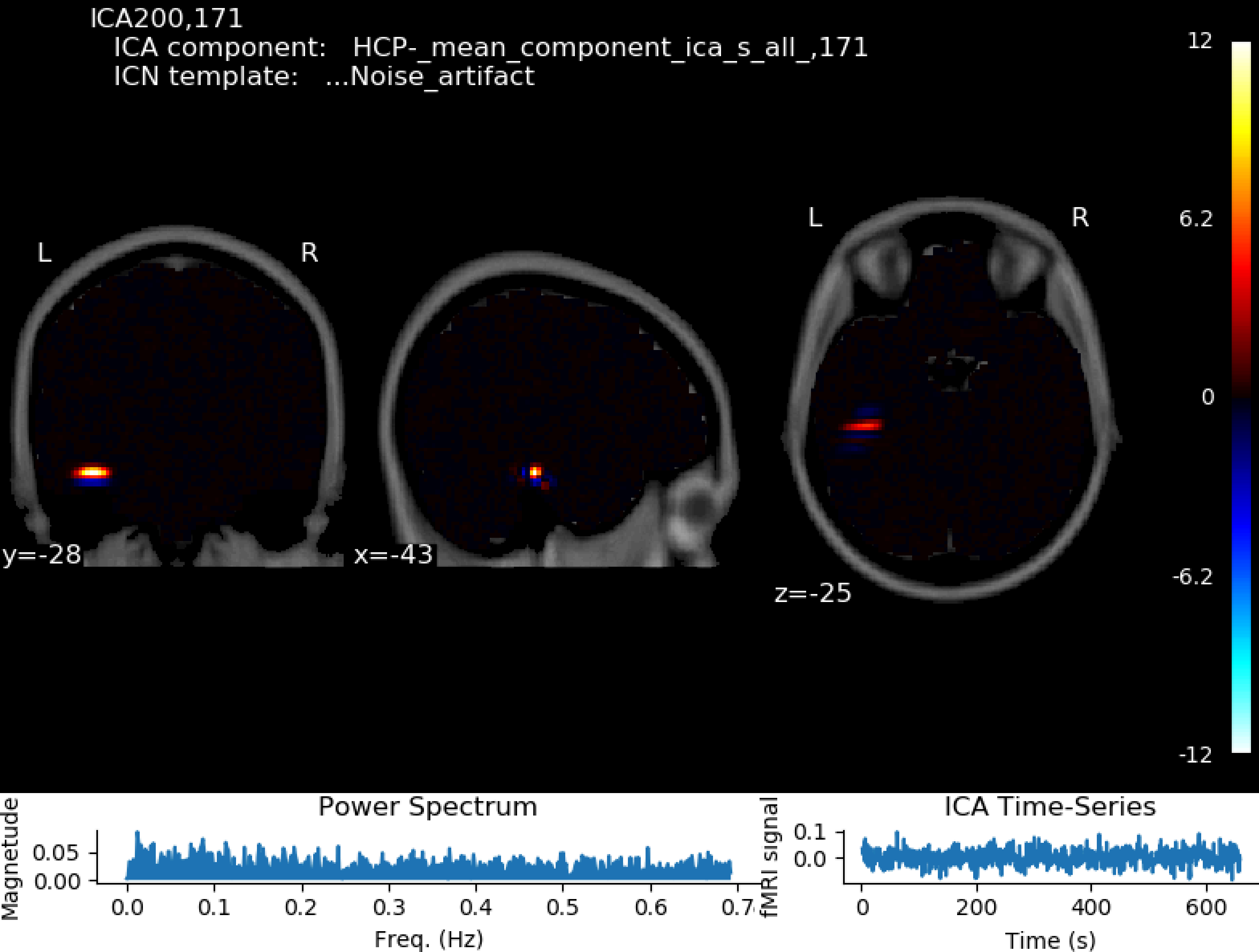
ICA200,171
ICA component: HCP-_mean_component_ica_s_all_,171
ICN template: ...Noise_artifact
L
R
y=-28
x=-43
L
R
z=-25
12
6.2
0
-6.2
-12
Power Spectrum
Magnetude
0.05
0.00
0.0
0.1
0.2
0.3
0.4
0.5
0.6
0.7
Freq. (Hz)
ICA Time-Series
fMRI signal
0.1
0.0
0
200
400
600
Time (s)

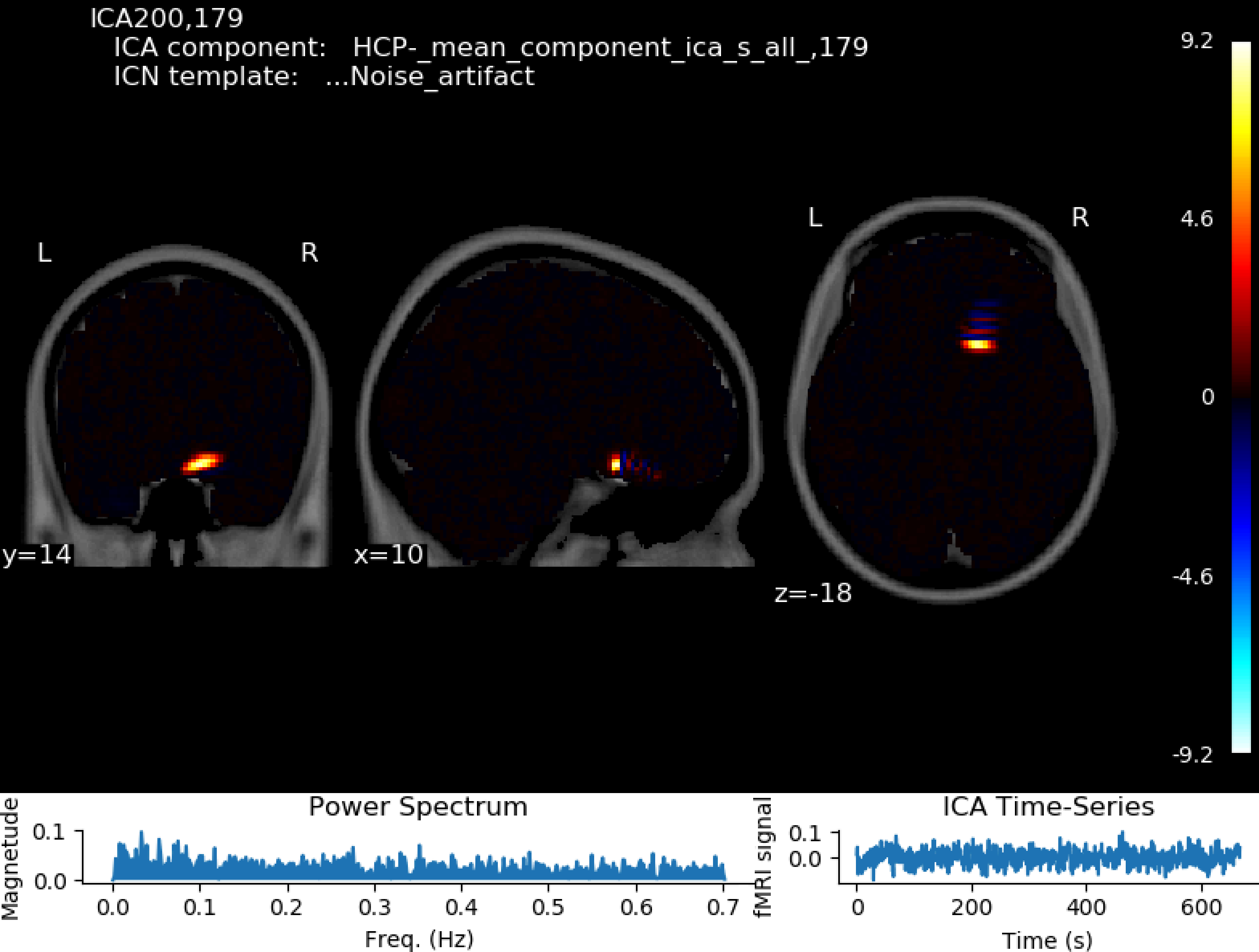

ICA200,179
ICA component: HCP-_mean_component_ica_s_all_,179
ICN template: ...Noise_artifact
L
R
y=14
x=10
z=-18
9.2
4.6
0
-4.6
-9.2
Power Spectrum
Magnetude
0.1
0.0
0.0 0.1 0.2 0.3 0.4 0.5 0.6 0.7
Freq. (Hz)
ICA Time-Series
fMRI signal
0.1
0.0
0 200 400 600
Time (s)

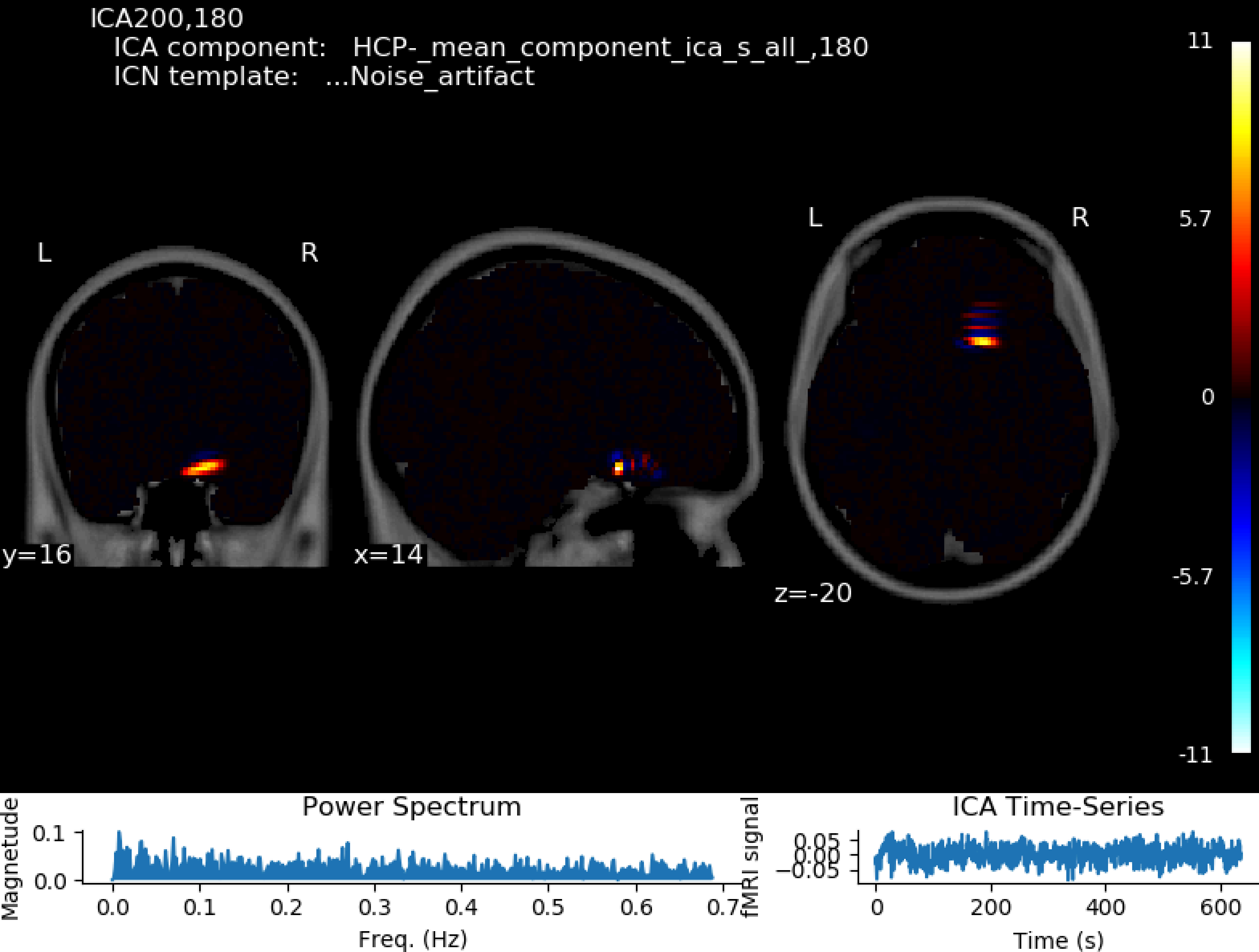
ICA200,180
ICA component: HCP-_mean_component_ica_s_all_,180
ICN template: ...Noise_artifact
L
R
y=16
x=14
z=-20
11
5.7
0
-5.7
-11
Power Spectrum
Magnetude
0.1
0.0
0.0
0.1
0.2
0.3
0.4
0.5
0.6
0.7
Freq. (Hz)
ICA Time-Series
fMRI signal
0.05
0.00
−0.05
0
200
400
600
Time (s)

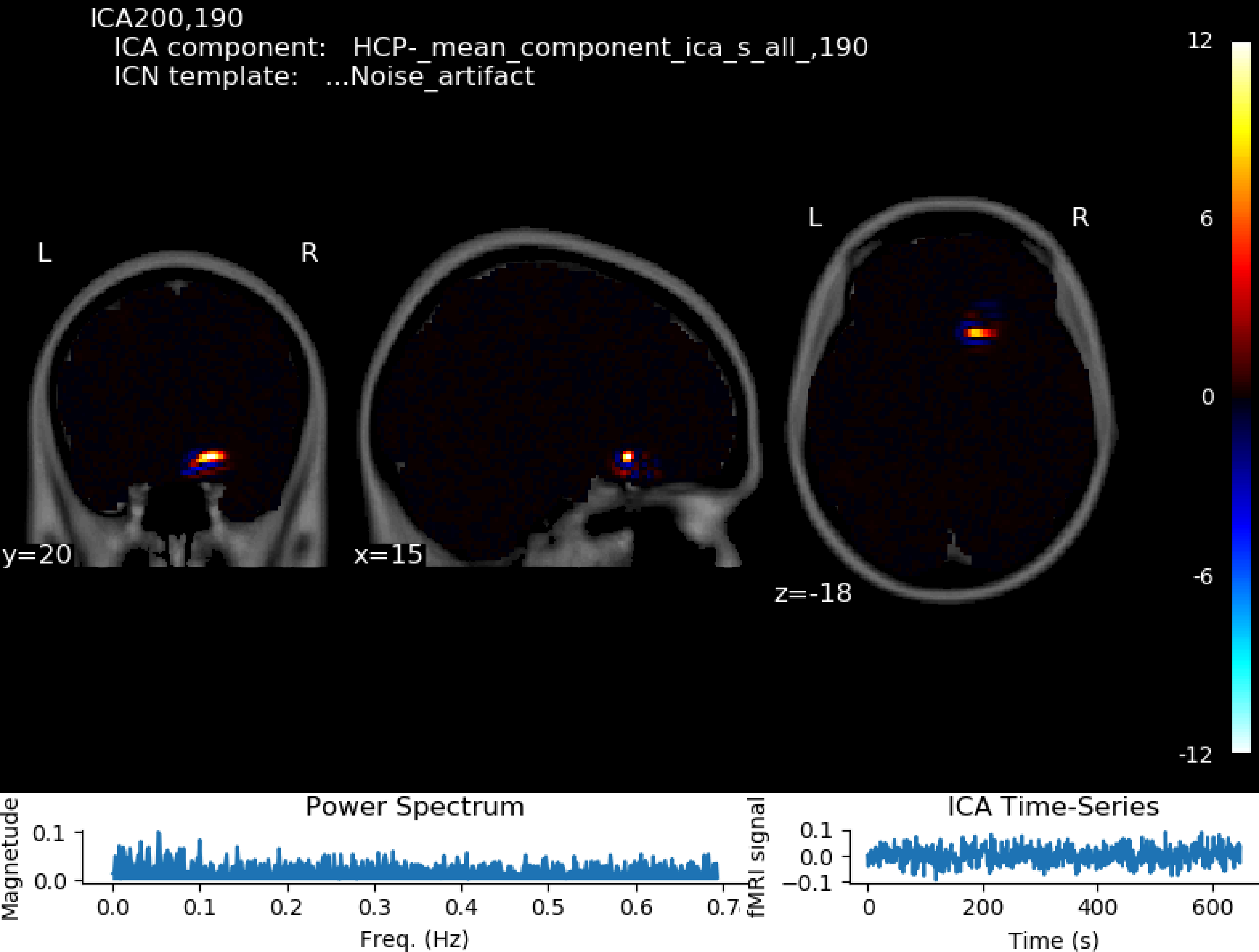
ICA200,190
ICA component: HCP-_mean_component_ica_s_all_,190
ICN template: ...Noise_artifact
L
R
y=20
x=15
z=-18
12
6
0
-6
-12
Power Spectrum
Magnetude
Freq. (Hz)
ICA Time-Series
fMRI signal
Time (s)

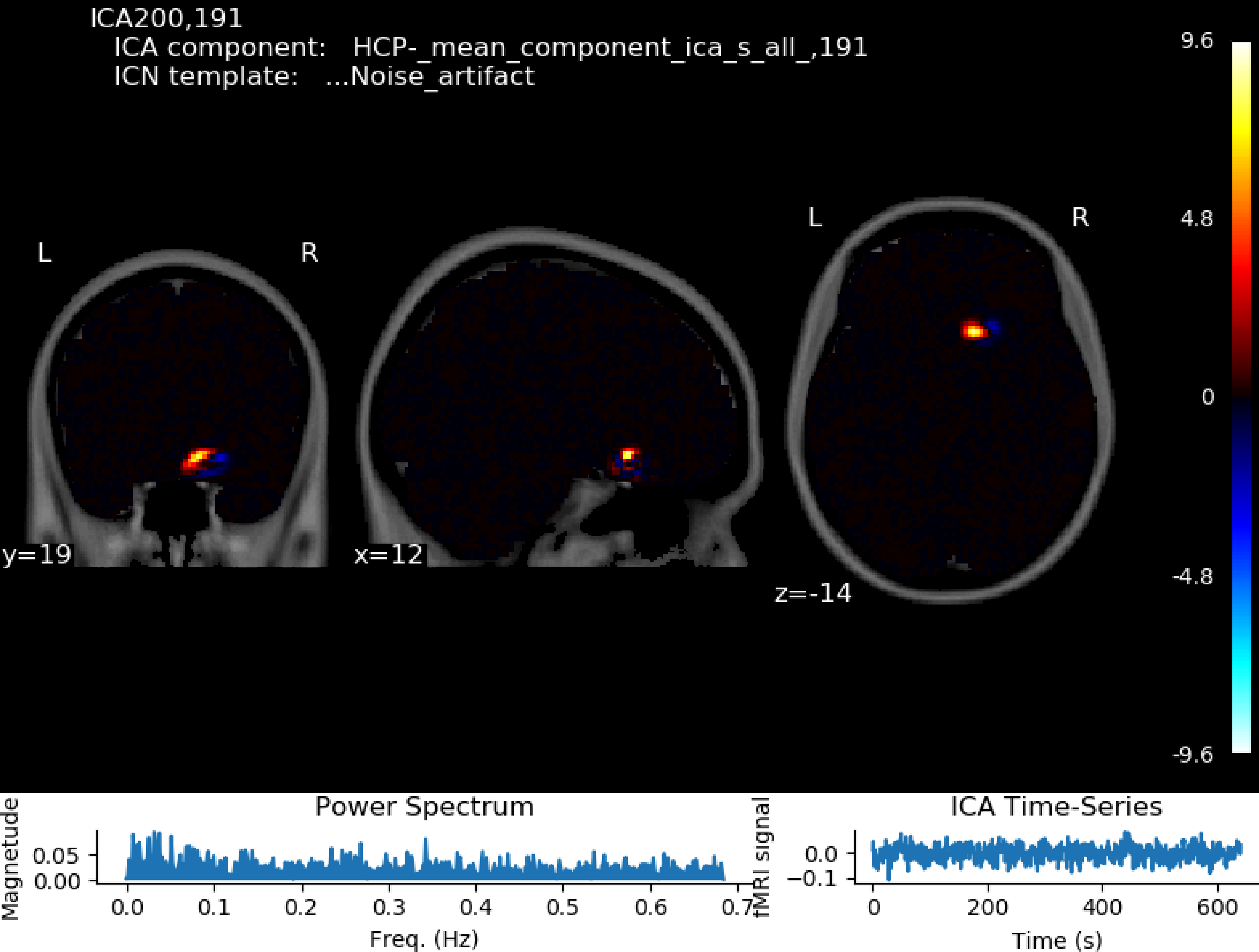
ICA200,191
ICA component: HCP-_mean_component_ica_s_all_,191
ICN template: ...Noise_artifact
L
R
L
R
y=19
x=12
z=-14
9.6
4.8
0
-4.8
-9.6
Power Spectrum
Magnetude
0.05
0.00
0.0
0.1
0.2
0.3
0.4
0.5
0.6
0.7
Freq. (Hz)
ICA Time-Series
fMRI signal
0.0
−0.1
0
200
400
600
Time (s)

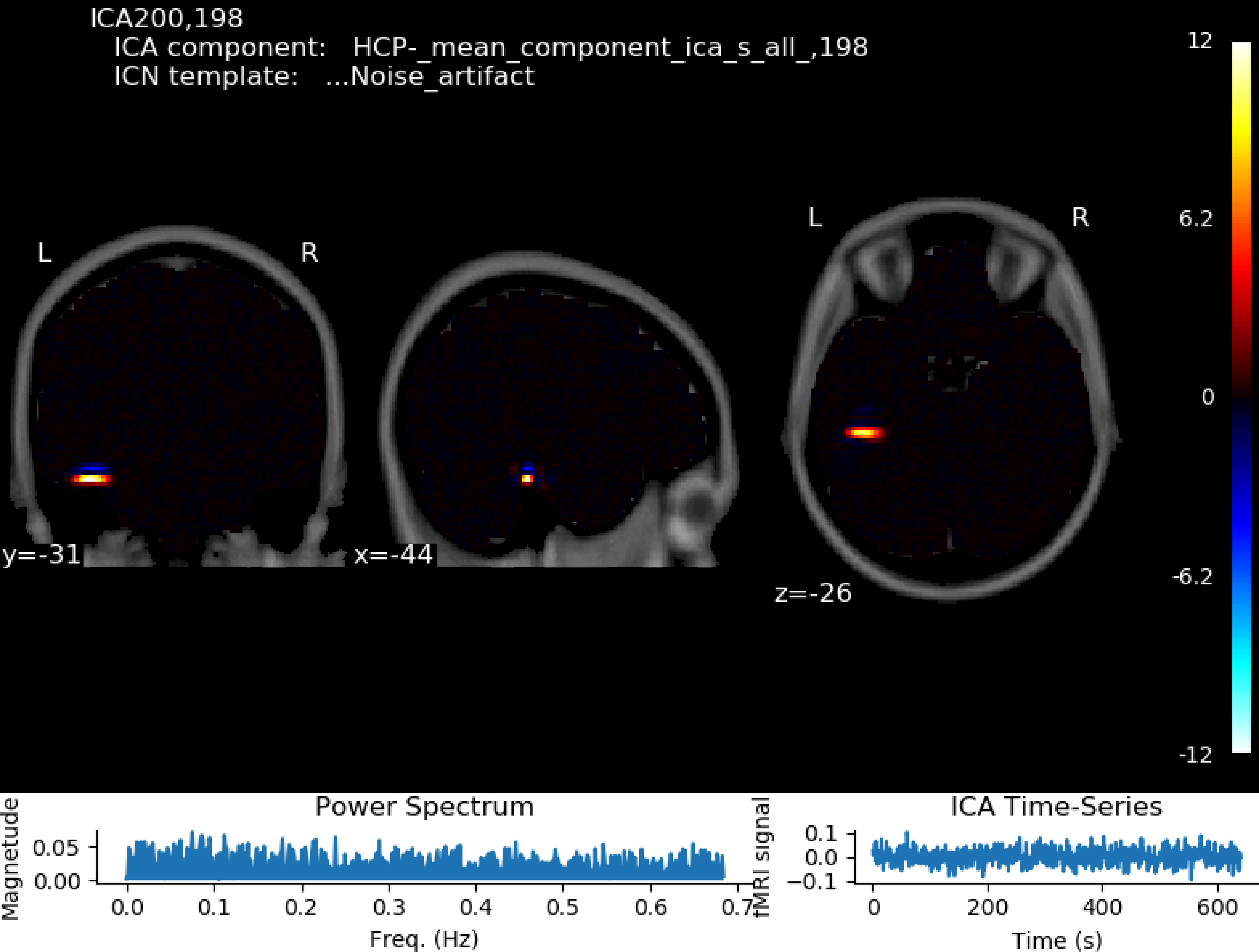
ICA200,198
ICA component: HCP-_mean_component_ica_s_all_,198
ICN template: ...Noise_artifact
L
R
y=-31
x=-44
L
R
z=-26
12
6.2
0
-6.2
-12
Power Spectrum
Magnetude
0.05
0.00
0.0
0.1
0.2
0.3
0.4
0.5
0.6
0.7
Freq. (Hz)
ICA Time-Series
fMRI signal
0.1
0.0
−0.1
0
200
400
600
Time (s)

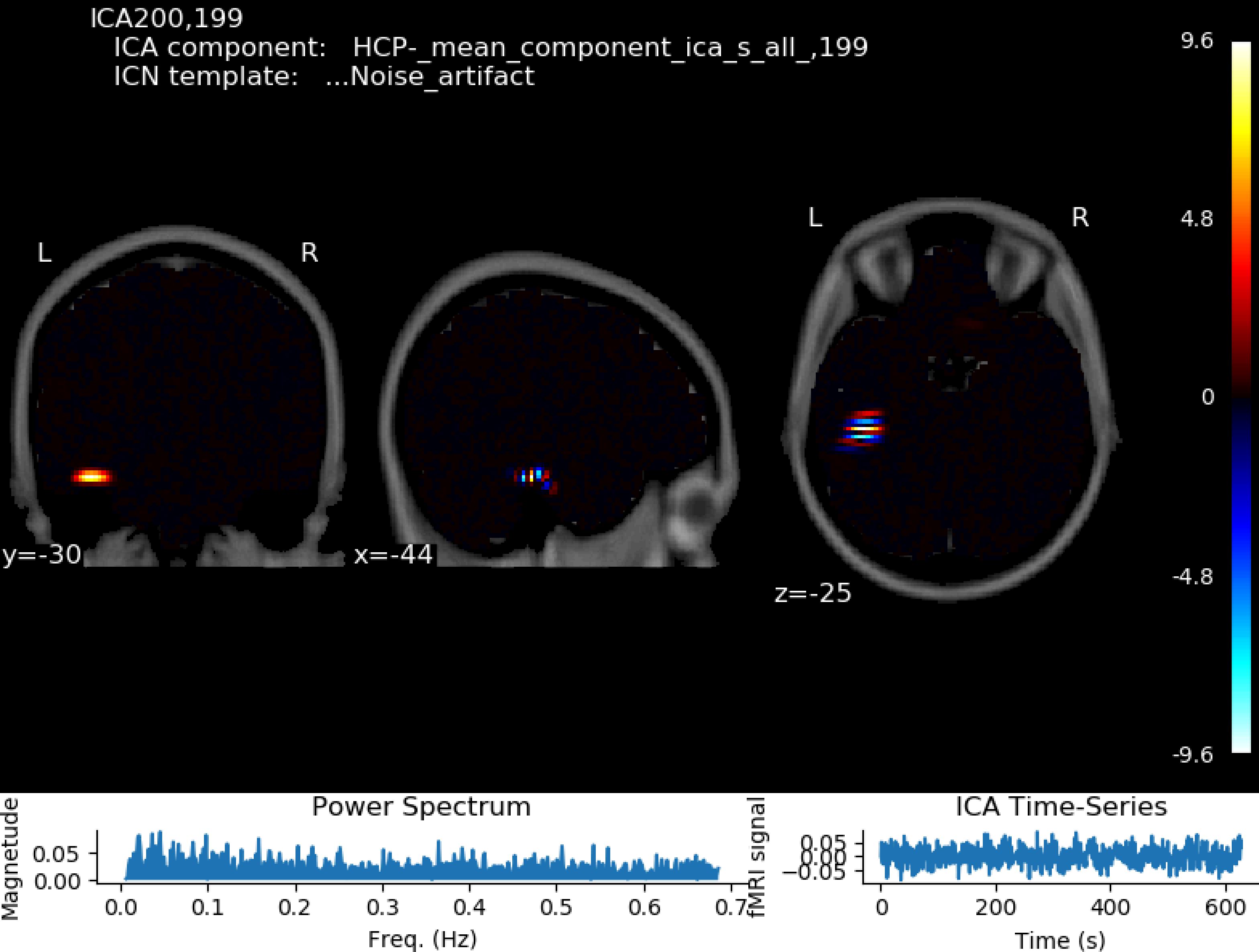
ICA200,199
ICA component: HCP-_mean_component_ica_s_all_,199
ICN template: ...Noise_artifact
L
R
y=-30
x=-44
z=-25
9.6
4.8
0
-4.8
-9.6
Power Spectrum
Magnetude
Freq. (Hz)
ICA Time-Series
fMRI signal
Time (s)
