# Supplementary material for: Stable Meta-Networks, Noise, and Artifacts in the Human Connectome: Low- to High-Dimensional Independent Components Analysis as a Hierarchy of Intrinsic Connectivity Networks
Source: Front Neurosci. 2021 May 6;15:625737. doi: 10.3389/fnins.2021.625737 (PMC8134552; doi:10.3389/fnins.2021.625737)
Supplement: Supplementary file 8 [file Data_Sheet_1.DOCX]

Supplementary Material

# Supplementary Figures and Tables

## Supplementary Figures

**Supplementary Figure 1.** Bootstrap convergence of IC stability indices by network (*I_q_*) for sample percentiles, at model orders ICA_20_ and ICA_70_. With the exception of minimum *I_q_*, all sample percentiles converged in both ICA models, with as few as 10 bootstrap replicates.

**Supplementary Figure 2.** Group PCA dimensionality and the Somatomotor Network (SMN) from snowball ICA. The SMN was absent from the lowest ICA model orders, ICA_2_ and ICA_3_. This may be due to either the span of the group PCA space, or the limitation of the ICA algorithm. **a.** ICA was repeated, with increased group PCA dimensionality to 30. Resulting components, at ICA model orders 2 (left) and 3 (right), encompassed cerebrospinal fluid and parenchyma. Likely artifacts or nuisance sources. Compare with main Figure 3 for non-artifactual components at same ICA model order, group PCA dimensionality of 2. **b.** To show that the Somatomotor Network (SMN) was contained with the group PCA space with a dimensionality 2, but not the main results, a snowball ICA was performed (Hu et al. 2020). The most stable component was subtracted from results, and an ICA with group PCA space dimensionality of 2 was repeated. Resulting component encompassed bilateral somatomotor cortices. The SMN is thus contained within the span of the low-dimensional group PCA space. Its absence from ICA2 likely results (see main Figure 3) results from the limitations of ICA. **c.** SMN template, from Yeo et al. (2011).

**Supplementary Figure 3.** All spatial maps in ICA_4_ through ICA_9_. Shown are spatial maps overlayed with top-matching ICN templates (Yeo et al. 2011). In the top two rows, the coexistence of ICs matching both the main Visual Network and the Central Visual Network can be seen in ICA_5_. In ICA_8_, in contrast to the previous ICA model order ICA_7_, a bilateral Frontoparietal Control Network (FPCN) is absent from all spatial maps. Instead, a right-lateralized component (bottom row of ICA_8_) encompasses dorsolateral prefrontal and superior parietal cortices, consistent with the Right Executive Control Network (RECN). No spatial map encompasses the left homologue of this network, the Left Executive Control Network (LECN). At ICA_8_, the FPCN appears to be subdivided into subnetworks, with only one subnetwork, the RECN, present in the ICA model. At ICA_9_, spatial maps match the right- and left-lateralized partitions, respectively, of the bilateral FPCN template (bottom two rows).

**Supplementary Figure 4.** All spatial maps in ICA_20_ with template matches. Shown are spatial maps from ICA_20_ overlayed with top-matching ICN templates from either Yeo et al. (2011) or Shirer et al. (2012). Yeo-7 network template matches included the Dorsal and Ventral Attention networks (ICA_20,11_ and ICA_20,19_, respectively). Yeo-17 network template matches included the Ventral and Dorsal Somatomotor networks (ICA_20,1_ and ICA_20,5_, respectively), as well as the Central and Peripheral Primary Visual networks (ICA_20,9_ and ICA_20,12_, respectively). Shirer-14 network template matches included the High Visual (ICA_20,2_), Precuneus (ICA_20,3_), Auditory (ICA_20,6_), Anterior and Posterior Salience (ICA_20,7_ and ICA_20,20_, respectively), Right and Left Executive Control (RECN and LECN; ICA_20,13_ and ICA_20,17_, respectively), and Language (ICA_20,16_) networks. The Default Mode Network was subdivided into anterior (ICA_20,15_), posterior (ICA_20,14_) subnetworks. A non-template IC encompassed the cerebellum (ICA_20,4_). In addition to the frequently-observed template sensory ICNs, an IC encompassed lateral visual association areas (ICA_20,8_), while another IC encompassed left-lateralized middle somatosensory cortex (ICA_20,10_). Finally, in addition to the RECN and LECN template matches, an additional IC encompassed bilateral dorsolateral prefrontal cortices (ICA_20,18_). Correlations with named templates listed in Supplementary Table 1.

**Supplementary Figure 5.** Examples of “spike and bump” ICA overfitting artifacts from ICA_200_. All spatial maps flagged by criteria in section 3.3 and main Figure 5 consisted of single unilateral, narrow, elongated hyperfocal spikes. Foci were rarely more than three voxels in width, always extending for approximately ten voxels in the lateral direction of slice acquisition. Very similar or identical spatial maps were flagged at other ICA model orders.

**Supplementary Figure 6.** Specificity of cutoff guidelines for non-nuisance, non-artifactual components, displayed as a Voxel Probability Inclusion volume (see equation (3)). After excluding components identified as nuisance sources (see main Figure 3) or likely overfitting artifacts (main Figure 4), Voxel Inclusion Probabilities for the remaining non-nuisance, non-artifact spatial maps were calculated (equation (3) with event *B* defined as complement of criteria used in main Figures 3 and 4). For each individual voxel, associated Voxel Inclusion Probabilities represent the probability of inclusion in the top 95th percentile of non-nuisance (cerebrospinal fluid or white matter) and non-artifactual overfitting source signals. Resulting spatial maps are strongly located within the gray matter, without extending into the cerebrospinal fluid or white matter, indicating a high degree of specificity for neuronal source signals.

## Supplementary Table

**Supplementary Table 1.** Template matches and correlations for all components in ICA_20_. Top matches, ordered by IC, and correlations with ICN templates from either Yeo et al. (2011) or Shirer et al. (2012). ICN templates from Yeo et al. 2011 matching components include Somatomotor, Visual, Dorsal and Ventral Attention, Frontoparietal, and Default Mode networks. ICN templates from Shirer et al. (2012) include High Visual, Precuneus, Auditory, Anterior and Posterior Salience, Left and Right Executive Control (LECN and RECN, respectively), and Language networks. Matches were assigned based upon highest correlation with ICN template.

*p < 10^-5^ FWE-corrected; NA: not applicable
